# On the variability of generative artificial intelligence methods in conceptual modeling: an experimental evaluation on combining causal maps

**DOI:** 10.1371/journal.pone.0361281

**Published:** 2026-10-27

**Authors:** Noé Y. Flandre, Cédric Daumas, Philippe J. Giabbanelli

**Affiliations:** 1 Old Dominion University (ODU) Virginia Modeling, Analysis, and Simulation Center (VMASC), Suffolk, Virginia, United States of America; 2 School of Mines in Alès (IMT Mines Alès), Alès, France; Oxford Brookes University, UNITED KINGDOM OF GREAT BRITAIN AND NORTHERN IRELAND

## Abstract

Large Language Models (LLMs) increasingly support time-consuming conceptual modeling tasks such as building or combining models from text. In this context, causal maps are labeled, directed graphs that represent causal relationships (edges) between selected factors (nodes) in a problem domain. Modelers control the output through the LLM’s prompt and decoding parameters such as temperature, and recent studies either use a default temperature or minimize it by expecting that it supports replicability. However, evidence about this expectation remains limited, as does our understanding of how different LLMs and decoding or prompting strategies shape results. We assess the extent to which findings are an artifact of the LLM or its variability rather than a reflection of the modeler’s controls. We focus on the common conceptual modeling task of combining models, exemplified through three algorithms that reflect a variety of prompting techniques. We use five LLMs (DeepSeek, Gemini, GPT, Qwen, Ministral) and different versions to account for the potential impact of the architectures. Using the Design of Experiments approach of factorial analyses (supplemented by paired tests and confidence interval checks), our results show that outcomes differ significantly across LLMs and versions. Depending on the algorithm, randomness from the LLM can be the dominating factor, responsible for up to 96% of the variation in results. This happens even if we directly control the decoding, as evidenced by our comparison of greedy, beam-search, and contrastive decoding. We find that prompt effects are systematic but metric-dependent, while decoding affects performance but there is no universally best strategy. Since our findings suggest that replicability of conceptual modeling studies using LLMs may be limited, future studies may report the *distribution* of performance measures alongside exact model, decoding, replication, and baseline-normalization settings.

## Introduction

Large Language Models (LLMs) are increasingly used to support conceptual modeling tasks, including model generation, model assessment, model matching, and model-to-text or text-to-model transformations (Table 1) in both academic and industrial applications [1,2]. These uses reflect a broader interest in reducing the effort required to create, use, and maintain conceptual models. However, the quality and reproducibility of LLM-assisted modeling remain uncertain because the outputs depend on prompt design, decoding settings, model choice, and residual variability in LLM execution.

**Table 1:** Studies that use LLMs for several tasks in conceptual modeling. None has analyzed the interactions of effects between prompt elements using a complete (i.e., factorial design); see background section on Factorial Design of Experiments. Temperature is either left to its default value or set to zero with the intent of supporting replicability. Values of N/A indicate that a study with a fixed prompt did not analyze interactions between prompt elements.

Ref	Prompt elements	Task	Models	Temp.	Design	Runs
[10]	Zero-shot vs few-shot (pos./neg. examples), task description, single vs multiple answers	Model matching	LLaMA-2 (six versions), airoboros-l2	0 (main model) or default (other models)	2^2^ DoE + alternatives	1*
[17]	Few-shot (when using GPT-4)		GPT-4, SpaCy, BERT	default	N/A	
[18]	Zero-shot vs few-shot, Chain-of-thought		GPT-4	default*	One at a time	
[19]	Zero-shot		LLaMA-2, GPT-3.5, Mistral, Vicuna, MPT, Falcon, Mamba		N/A	≈50
[20]	Zero-shot vs meta-properties documentation, flat vs hierarchical word representations	Model assessment	GPT-3.5, GPT-4		2^2^ DoE	30
[21]	Zero-shot	Model matching	Flan-T5, GPT-3.5, BERT		N/A	1*
[1]	Zero-shot	Model matching	ChatGPT-4o	default*	N/A	20
[22]	Definitions and examples from articles	Concept associations	GPT-4	0	N/A	1*
[2]	Zero-shot, multiple patterns	Model assessment	ChatGPT-4	default*	One at a time	1*
[15]	Zero-shot, Prompt decomposition (five steps), Chain of Thoughts, Graph of Thoughts	Model generation	GPT-3.5, GPT-4, Bard, LLaMA, LlaMA2, Alpaca, Falcon, WizardLM	0 (main) or default (for plan generation)	One at a time	3
[23]	Zero-shot		GPT-3, GPT-4	default*	Multiple combinations	1*
[24]	Fine-tuning (task, expected format, sample user prompt and expected output), conversion to abstract syntax trees and use of external algorithms for processing		Full process on GPT-3.5 compared with single prompt on Gemini 4 and GPT-4-turbo	default*	N/A	1*
[16]	Zero-shot		ChatGPT-4	default*	N/A	3

*Assumed in the absence of specific information in the articles.

A well-known source of variation in outputs is the prompt [3,4], which specifies the task, input representation, constraints, and optional guidance provided to the LLM (Fig 1). Prompt engineering studies show that small changes in wording or structure can substantially affect outputs, noting the *surprising sensitivity of LLMs to even trivial prompt variations* [5]. While constraining LLMs through more detailed prompts is intuitive, adding elements to a prompt is not necessarily better [6] because these elements may interact in unexpected ways. For example, a human may use examples and counter-examples because they are clearly seen as two different categories, but LLMs may be confused by providing both. This motivates an experimental design that studies prompt elements jointly rather than varying one factor at a time.

**Fig 1:**
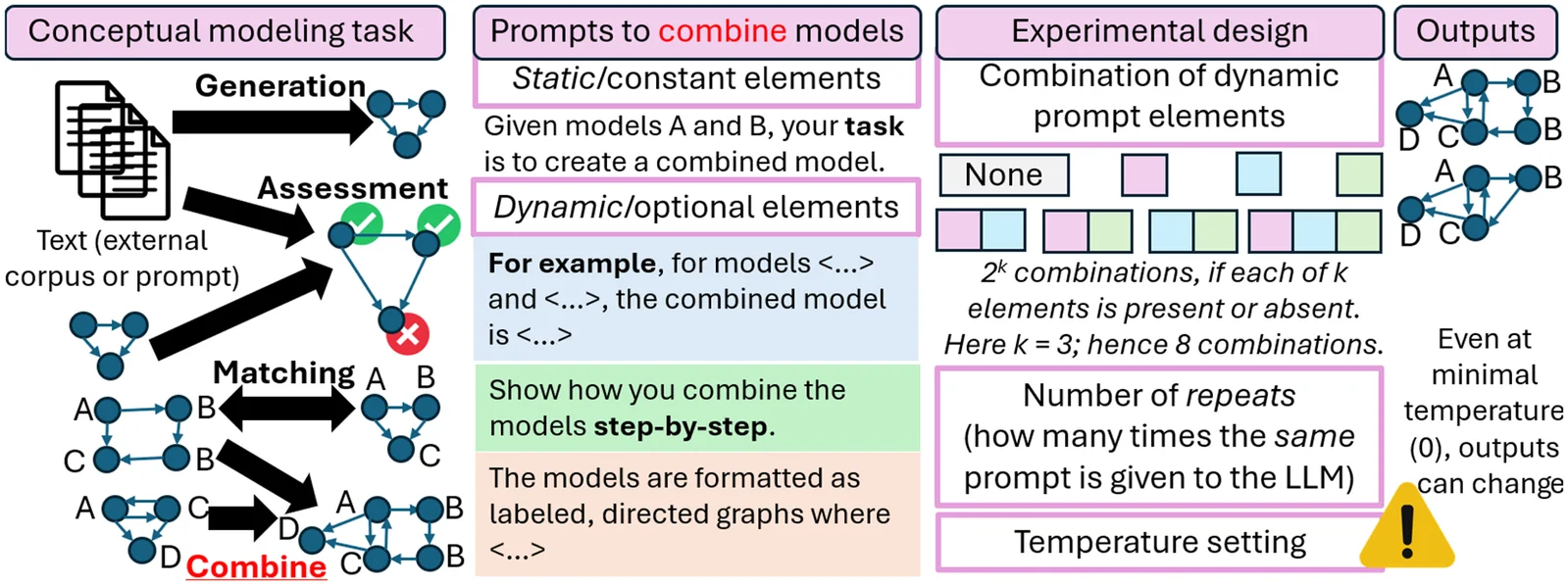
LLMs can support several conceptual modeling tasks. We focus on combining conceptual models. The result depends on the prompt (where several elements are optional) and the temperature. Repeats are needed, particularly as even a minimal temperature can produce different models.

A second source of variation is the generation process. A study on LLMs for conceptual modeling, noted “that there is plenty of variability when ChatGPT generates responses to same prompt” [7] – a point that was echoed in other conceptual modeling studies via LLMs [8]. For example, users are aware that a higher temperature would lead to more different outcomes even when provided with the same prompt [9]. Studies often use a default temperature (Table 1) or set temperature to zero with the expectation that a *negligible variability* in outputs (i.e., a quasi-deterministic output) would make the results reproducible [10,11]. However, repeated calls can still produce different outputs. Moreover, LLM family, version [12], architecture [13], and deployment conditions can all affect performance and variability. Conceptual modeling studies therefore need to assess whether observed results reflect the modeler’s prompt and input, or whether they are artifacts of the LLM and its execution.

Empirical approaches that were established when working with earlier architectures (e.g., GPT-3) may need to be revisited as we migrate towards more recent models. For example, GPT-4 is a sparse *Mixture-of-Experts model* and such models are not fully deterministic since input “tokens compete for available spots in expert buffers” [14]. In other words, only a subset of experts is activated for a given input and this sparse activation can introduce variability (even at temperature 0) since the stochastic routing mechanism may not always select the same experts for identical inputs. In addition, non-determinism can occur at the level of the implementation (e.g., when users place API calls to access an LLM), either through parallelism used to speed up GPU computations or in compounding effects stemming from small differences in floating point operations. As a result, scholars employing LLMs in conceptual modeling have reported that “despite setting the temperature and penalty parameters to zero [...] we observed inconsistencies in the outcomes when using identical prompts” [15]. This has led other researchers on conceptual modeling to call for more research and investigation on reproducibility [16]. Despite the variability in outputs, studies using LLMs for conceptual modeling either do not justify that enough replications are performed (e.g., using statistical methods such as confidence intervals) or they do not perform replications (Table 1), even when employing GPT-4. This habit inherited from prior architectures may be problematic for empirical studies, as reported results may constitute a single sample drawn from a distribution. There is thus *a pressing need to examine the extent to which the inherent variability of the LLMs may shape findings in empirical studies on conceptual modeling*. In addition, the interactions between prompt elements call for robust statistical frameworks that can adequately decompose the variance in results (e.g., accuracy in creating a model) as a function of individual prompting techniques or *combinations* thereof.

Building on these considerations, the task of *combining conceptual models* is used as a representative case to examine interactions between prompt elements and variability in LLM outputs. Specifically, we employ three algorithms to combine causal maps, which are conceptual models that consist of nodes with clearly interpretable labels (e.g., healthy diet, physical activity) connected by edges (e.g., physical activity relates to obesity). The three algorithms seek to bridge the existing causal maps by either suggesting direct connections (i.e., adding edges) or intermediary ones (i.e., adding labeled nodes and edges). These algorithms only serve to exemplify the breadth of strategies available in prior work, such as identifying related pairs of concepts and/or generating new concepts closely related to existing content (e.g., via text embedding). Our empirical study on conceptual modeling makes three contributions:

- We demonstrate that the prompting elements or the decoding approach controlled by the user may matter very little in situations where the inherent variability of the LLM is the dominating factor (even at a minimal temperature of zero), while the choice of LLMs and versions can significantly affect the importance of prompting elements. This suggests that empirical studies would need to repeat experiments and/or impose additional constraints on prompts to pro-actively reduce variability.
- We show that *prompting elements exhibit significant interactions*, thus the dominant practice in ablation studies of varying one-factor-at-a-time may need to embrace the practice of factorial Design of Experiments, which is well-established in the Modeling & Simulation community.
- Our results are achieved based on three algorithms that demonstrate a variety of techniques to combine models, across LLMs from five well-known providers (DeepSeek, Google’s Gemini, OpenAI’s GPT, Alibaba’s Qwen, Mistral’s Ministral), thus providing a comprehensive range of experimental settings.

The remainder of this paper is structured as follows. Our Background section keeps this manuscript self-contained by providing a succinct overview of prompt engineering as it pertains to conceptual modeling, along with a brief introduction to the factorial Design of Experiments (DoE) employed in our study (a longer overview is provided in Supplementary Document S4 Guide), and an overview of decoding methods for LLMs. Next, we present the three LLM-based algorithms used to combine conceptual models and two non-LLM baselines for comparison. We explain how we decompose performances (e.g., precision, accuracy, recall) as a function of variability, individual prompting elements (e.g., examples, explanations), decoding approach, and their interactions. Our empirical results on several maps pertaining to obesity and suicide prevention are then reported and subsequently discussed. To support replicability, our dataset and scripts are openly accessible on a third-party permanent repository at https://doi.org/10.17605/OSF.IO/KGDRU.

## Background

### Combining conceptual models

Surveys and case studies indicate that practitioners often work with multiple models [25] that provide insight into a system from different perspectives, for different tasks, or at different levels of abstraction [26]. In *participatory modeling*, each participant (e.g., a subject matter expert) may produce a model with a facilitator, then individual models are combined to arrive at a group-level representation [27]. In such settings, individual maps should not be seen as ‘noisy attempts’ to recover a universal ground-truth. Rather, the participants’ maps represent their mental models of the socio-environmental system, which may not be a fully and directly observable system. An aggregate model in this context should be understood as a group-level representation, or as a basis for comparing groups of participants, but not as a model that any single participant already holds in full [28]. In this paper, we use a group-level model for obesity and individual models for suicide prevention, thus showcasing that models represent various facets of the same domain.

Since individual models capture some facets of a system based on the expertise and/or lived experiences of individuals [25], aggregating them into a group-level model allows to capture a larger system. However, combining models is not a straightforward task: they are not necessarily at the same level of granularity and may use different terminology (Fig 2). A ‘patient’ in one perspective (e.g., a clinician’s model) becomes a ‘beneficiary’ for payments, a ‘clinical record’ is called a ‘case file’ for the administration, and even clinicians may refer to a ‘visit’ as a ‘patient encounter’ or ‘consultation’. Freixa and Fernández-Silva have suggested that “the number of terminological variants used to refer to a concept seem endless” [29], thus stressing one of the main challenges in combining conceptual models. This aspect of combining models is known as *perceptual integration*, as it deals with establishing interdependence (or a *match*) between system elements across models [26]. Many algorithms have focused on identifying semantically related concepts across models by using structural information (e.g., two concepts are more related if they have a shared target) and lexical matching from WordNet [30,31], domain-specific thesauri [31,32], or word embeddings [33,34].

**Fig 2:**
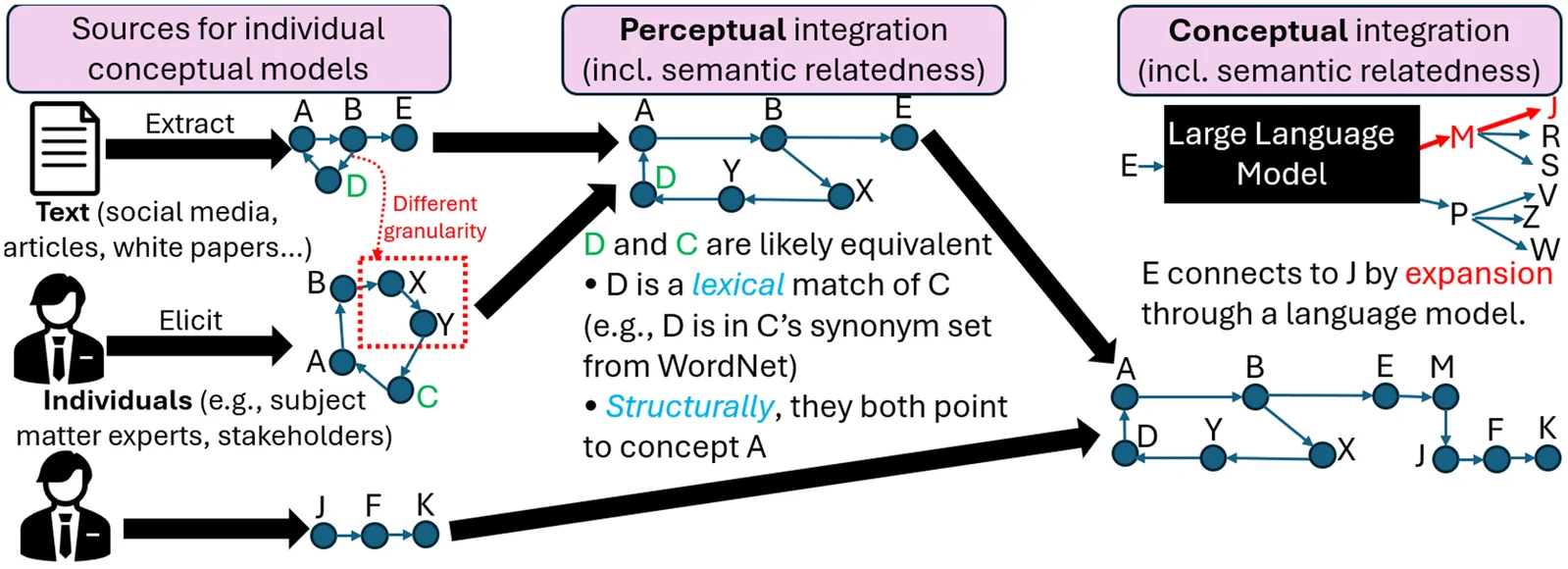
Individual models may originate from diverse sources, use various representation standards, and depict different (sub)parts of a system. Their integration involves a perceptual and conceptual aspect.

The other challenge in combining models is the *conceptual integration*, whereby hypotheses about the system of interest may need to be *inferred* from the various models. Even if the models used the same terminology so that their constructs could immediately be linked, simply aggregating them may lead to errors due to interactions between the models – these issues are captured in the portmanteau of ‘integronsters’ [35] coined to evoke integrated monsters. That is, the combined model may suggest dynamics about a system that were not produced or endorsed by any of the individual models [28]. Alternatively, it may seem impossible to connect the models solely based on their information: a psychological model may cover depression and stress, while a physiological model may include biomarkers such as cortisol and depression. Cortisol is not a synonym for stress, so the models would be seen as completely disjoint. But if we can infer that stress releases cortisol into the body, then we can begin to link the models. A strategy is thus to use an LLM to *expand* the concepts that we seek to connect until it goes on a tangent or we find a shared construct [36]. In this case, the LLM serves as additional knowledge source to bridge models or ensure their integration. Combining models through the inference capabilities of an LLM differs from some definitions of merging and integrating models, whereby a merger only ‘adds’ a model to another without changes, and an integration alters one of the models so that it fits within the other [37]. In contrast, involving an LLM is an opportunity to identify a variety of relations (e.g., part-of) and even intermediates to relate constructs across models.

### Prompt engineering

LLMs such as GPT-4o do not provide direct access to the model or to the training data, thus users need to employ prompts to express their goals. The construction of a prompt depends on the required output, the task, and the type of input. For details, we refer readers to [38–40]. We provide two illustrations for the types of prompts involved in combining models. First, consider the SpaCy/GPT-4 method by Snijder and colleagues [17] to match two models describing occupations. The LLM is presented with a list of candidate matches, established by picking the top 5 based on cosine distance between the embeddings of potential matches and the target concept. The authors used the following prompt:

**Prompt from [17]**

From this list of matched occupations, decide which of the pairs is the most correct. Pick at most one pair. Only output the pair without any extra words, characters or symbols. The candidate pairs are: \ n {formatted string}

In another approach, the authors gave GPT-4 a candidate pair and provided lexical as well as structural information (incoming and outgoing concepts):

**Prompt from [21]. Line breaks are omitted for brevity.**

Given the lists of names and hierarchical relationships associated with two concepts, your task is to determine whether these concepts are identical or not. Consider the following: Source Concept Names: list of concept names Parent Concepts of the Source Concept: list of concept names Child Concepts of the Source Concept: list of concept names *...same for the target concept...* Analyze the names and the hierarchical information provided for each concept and provide a conclusion on whether these two concepts are identical or different (‘Yes’ or ‘No’) based on their associated names and hierarchical relationships.

The structure of the prompt has a significant effect on the ability of the LLM to perform the task. Macilenti *et al.* showed that seven variations of the prompt resulted in vastly different performances, with recall (on correctly matching two concepts) ranging from 0.52 to 0.92 [41]. For example, the objective could be worded differently, thus forcing the LLM to accomplish the entire task (“provide the most accurate matching”) or allowing it to break down the problem into smaller steps (for an object of the first model which object of the second model is the best match).

In all three studies, the prompts use the common *zero-shot* approach, where no example is provided. The LLM can be guided through examples and definitions either through the prompt, such as by providing a single (*one-shot*) or a handful of examples (*few-shot*) [42], or by add-on modules that provide new embeddings [43]. For instance, when using the LLM to ideate potential conceptual models given a set of constraints, it is common to fine-tune the LLM by providing a curated set of models that convey design expectations [44]. In addition to examples, a prompt may include *counterexamples* to prevent undesirable behaviors (e.g., by starting with “Do not provide this type of answer”) [45]. However, empirical studies reveal that LLMs may struggle with such ‘negated prompts’ [46]. Collectively, examples and counter-examples constitute ‘in-context demonstrations’, also known as in-context learning. Studies have also shown that it is important to align the *data representation* with the task at hand, particularly when employing graphs, which underpin many types of conceptual models. For example, an LLM may prefer a list of relationships when asked to count the number of relationships in a model, but it is more accurate at reporting the number of relationships involving a specific concept when given a list of neighbors for each concept [47].

### Factorial Design of Experiments (DoE)

The previous subsection showed that a prompt used in conceptual modeling may involve several optional parts, such as whether to include examples or counter-examples. The output of the LLM thus depends on the specific combination of prompt elements. A simple analysis is to vary One Factor At a Time (OFAT): for example, we *always* provide examples along with counter-examples, and we only check whether to request a step-by-step approach. Alternatively, we can use Design of Experiments (DoE) techniques to precisely identify how the variation in the LLM output is driven by individual parts of the prompt and their combinations. A DoE is superior to varying one factor at a time because it identifies interaction effects between factors, provides a more comprehensive understanding of what drives the LLM’s output, and is more efficient, requiring fewer runs to explore multiple factors. In particular, a DoE supports robust statistical analyses to quantify the significance of the prompt elements and their interactions, which enables a better optimization. Supplementary material S4 provides a longer overview of DoE and tools that support modelers in performing such analyses.

While DoE is a well-known branch of applied statistics when it comes to simulation studies [48,49], it is less commonly employed in the conceptual modeling literature thus we briefly explain its core principles. The goal of DoE is to decompose variation in an output (e.g., accuracy at combining conceptual models) as a function of individual conditions (e.g., whether to include examples in a prompt) and their interactions. Each condition is called a *factor* and the number of options available is known as *levels*. For example, if a prompt may consist of 1, 2, or 3 examples and whether to convey a graph as a matrix or a list, then we have two factors (examples, representation) with 3 and 2 values respectively. There are thus 3×2 combinations of experimental settings. However, the simple OFAT approach may only cover four of these combinations: (i) using two examples and varying matrix vs. list representations, (ii) varying the number of examples while using a matrix representation. The difference between varying one factor at a time and considering all combinations grows exponentially: if there are *k* factors and each one is reduced to a binary choice, there are $2^{k}$ potential experiments and only *k* + 1 of them would be performed with the OFAT approach. In addition, when there is stochasticity in the response, each experiment is performed *r* times, leading to a $2^{k}r$ design known as *full factorial design with replication*.

Fig 3 exemplifies a DoE with 3 binary factors, that is, a 2^3^ full factorial design. The three factors correspond to whether the prompt includes a task description or not (*A*), whether the model should be a list of linked concepts or a matrix (*B*), and whether we provide examples or not (*C*). Fig 3 shows that we consider both the individual factors (*A*, *B*, *C*) and all possible interactions (*AB*, *AC*, *BC*, *ABC*). The accuracy of the LLM in combining conceptual models is recorded as the response variable *y*. The analysis consists of decomposing the variance in the response *y* as a function of all factors and their interactions. In this example, 70.92% of the results are related to the examples (*C*). As we consider larger groups of factors, their contributions to the variance decreases. In this example, we note that the task description and the model format have a small interactive effect that drives 4.43% of the variance.

**Fig 3:**
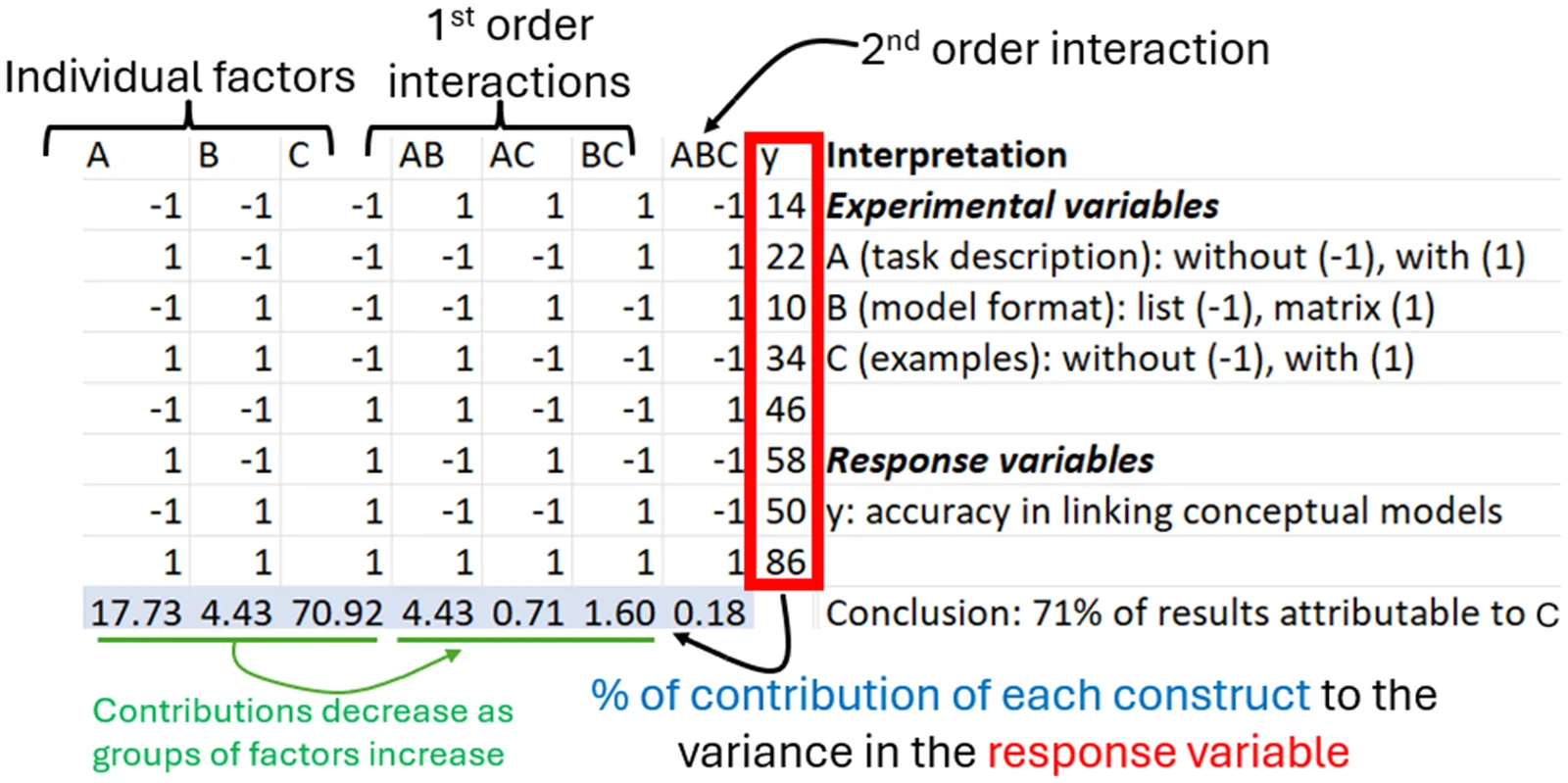
Design and analysis of an empirical study using a 2^3^ full factorial design. In a binary factorial design, values are encoded as −1 and 1 to facilitate cross-products computations in the ensuing analysis. For simplicity, this version does not include replications. When replications are present (as in our study), the response variable is recorded several times and the error becomes one of the constructs in the analysis.

In sum, a *full factorial design with replication is a systematic and transparent statistical approach to assess how each potential part of a prompt may shape our ability at combining conceptual models*. The use of factorial designs as a method for studying the effects of multiple factors simultaneously, as opposed to varying one factor at a time, was introduced in the 1930s [50] and is well-known in simulation studies [48,49]. However, to the best of our knowledge, *this method has not yet been used to investigate the design of LLM prompts in conceptual modeling studies* (Table 1). As a result, these studies could not report how much of their results were driven by randomness rather than prompt elements.

### Potential sources of variability in LLM outputs

In a recent article, we detailed how non-determinism is observed in and produced by LLMs [51]. Even when we repeatedly use the same prompt and set hyperparameters such as the temperature to 0, it is *possible* that the output produced by the LLM will be very different, as reported in several studies [52,53]. Early studies popularized the metaphor of LLMs as ‘stochastic parrots’ [54] (a characterization that is debated in more recent views [55]), which may be misinterpreted to suggest that LLMs simply produce ‘noise’. Several clarifications are important in this context.

First, *residual variability should not automatically be interpreted as meaningless noise*. Some variability may reflect an engineering issue, such as when different versions of an LLM are assigned at runtime (i.e., instability in model identity as detailed below), so some of the prompts are handled by models with different levels of precision. This possibility can sometimes be diagnosed when raw outputs can form clusters showing distinct levels of specificity, formatting consistency, or numerical exactness, suggesting that different backend implementations or model variants were used. Variability may also be informative when it reveals that the prompt and the input representation do not sufficiently constrain the task. For example, in conceptual modeling, the LLM may identify several plausible bridges between two models, several candidate intermediate concepts, or several acceptable ways to express a relation. In such cases, repeated runs may not fluctuate around one stable answer; rather, they may explore different parts of the space of plausible conceptual inferences. When outputs vary while remaining within a consistent distribution of solutions (i.e., similar patterns of nodes, edges, or relations appear across runs), we have *distributional reproducibility* [56]. When outputs differ at the surface level while preserving the same underlying conceptual structure (e.g., there may be slight differences in wording but the overall decision is the same), then we have *semantic stability* [56]. In short, a large residual component can have multiple causes (e.g., unstable execution, task with low constraints), and these interpretations should not be conflated.

Second, to appreciate what ‘variability in execution’ means and how it can be (partly) controlled, recent papers distinguish between sources of variability [56]. Three different sources of variability are often conflated: inference non-determinism concerns *how* a fixed model is executed, model identity is about *which* model is actually being provided to a user, and distributional bias concerns *what kinds of continuations* the model has learned to prefer (i.e., predicting the next token).

More precisely, *inference non-determinism* refers to variability that arises because the *execution* of a model is not perfectly stable. Even when we use the same prompt and the same nominal model, the software and hardware environment used to run the model may not carry out the computations in exactly the same way. For example, requests may be grouped differently (*batching*), work may be divided differently across several graphics processors (e.g., *tensor parallelism*), or numerical operations may be performed in a different order. In some settings, these differences are enough to change the token probabilities and eventually the final output [51,57]. Second, the *identity of the model being served* may also be unstable. That is, when users rely on a service such as OpenRouter and ask for a model by name (e.g., DeepSeek R1), it does not guarantee access to the same weights and behaviors. A routing platform may send requests to different providers, who can use different numerical precisions or inference optimizations. In addition, a model can be ‘silently forwarded’ after deprecation (i.e., a routing platform would automatically redirect queries to a newer version), or a model name can function as an alias that points to the provider’s current version rather than to one immutable system. These are not issues of how a model is executed, but rather which model is being served [51], which is a deployment matter beyond the scope of this article. Third, *distributional bias* is about what the model has learned to prefer: since LLMs are trained to model conditional token distributions, they assign higher probability to some continuations than to others. As a result, they may favor outputs that are common, fluent, or statistically likely even when the task itself is deterministic and those outputs are not the desired ones [51].

Since there are different sources of variability, analyzing and controlling them requires different approaches. The factorial analysis explained in the previous subsection decomposes how much of the variation in performance is attributable to specific aspects (individually and in combination) such as whether to include one prompt element or not, or which parameter value to use (e.g., temperature set to 0 or 1). A factorial analysis operates on observed inputs and outputs at the experimental level: it can tell us that variation remains after *accounting for the factors that we manipulated*, but it does not observe the internal computations through which token probabilities are produced, propagated, and decoded, nor the system-level details of how those computations are distributed across hardware. Other approaches are thus needed to observe or control such aspects. For example, in conceptual modeling, it is important to examine the *structure of the outputs returned by the LLM*, not only their aggregate performance. Repeated runs may vary only marginally around the same candidate nodes and edges, which would suggest a relatively stable underlying solution, or they may instead produce substantially different sets of concepts and links, which would indicate that the LLM is exploring distinct plausible solutions.

Two forms of control are of particular interest in this paper. First, the most classic approach is to *control through prompts* since they shape which cues are made salient to the LLM, how the task is framed, what constraints are emphasized, and what output format is expected. Although prompts do not change the model’s weights, they constrain the *space of admissible interpretations* that the LLM may explore. These constraints can be strong in some settings and weak in others: LLMs may reproduce context-sensitive patterns when the relevant cues are clearly exposed, yet fail on closely related higher-order judgments when a zero-shot prompt does not sufficiently scaffold the intended reasoning [58,59]. In the case of conceptual modeling, using various prompt elements (e.g., examples, counter-examples, explanations) can thus constrain how conceptual bridges between models should be inferred. Second, we can control how the LLM converts the probability distribution over possible next tokens into text (i.e., the *decoding mechanism*). Modern LLM APIs such as OpenAI’s GPT predominantly involve some *sampling-based decoding*: we draw from the distribution based on a *temperature* parameter, where low values concentrate on the most likely continuations and higher values allow more diversity. By contrast, access to alternative decoding strategies usually requires *open-weight* models that the user can run in a local or otherwise fully controlled inference environment, because these strategies must be chosen at inference time by the software that executes the model (e.g., Hugging Face Transformers), but LLMs accessible only via APIs typically restrict the set of generation controls. *Greedy decoding* is deterministic and simple by design: there is no randomness and no parameters; it always selects the most probable next token. Note that it reduces variability but does not necessarily eliminate it, since other events during execution can produce variability [56]. *Beam search* does not keep only the single most likely continuation at each step. Instead, it expands each partial continuation by considering all possible next tokens, then retains only a fixed number of the highest-scoring candidates. In this way, it performs a pruned search over multiple possible sequences, approximating the highest-probability output under the model [60]. Its main parameter is the *beam width*, that is, the number of candidate sequences kept at each step. A larger beam width explores more alternatives and often yields more stable, high-probability outputs, but it also increases computational cost. Finally, *contrastive decoding* tries to avoid repetitive or generic text by choosing a token that is likely according to the model, while avoiding one that is too similar to what has already been generated [61]. In practice, this strategy usually involves two parameters: one determines how many high-probability candidate tokens are considered at each step, and the other determines how strongly similarity or degeneration is penalized. Increasing the candidate set allows more alternatives to be examined, whereas increasing the penalty emphasizes diversity.

While the analyses and aforementioned decoding mechanisms are well-defined and have been used in empirical studies in several domains [62–64], they are not routinely employed when using LLMs for Modeling & Simulation. Although factorial designs have occasionally been used when translating simulations into text (e.g., to generate the journey of a single simulated agent [65] or to summarize outputs from an agent-based model [66]), there remains a paucity of studies that apply factorial analyses at the *conceptual modeling stage*. In particular, prior work has largely focused on evaluating outputs (e.g., readability, coherence, or alignment with reference texts) rather than systematically decomposing how prompt elements shape the construction of conceptual models themselves (Table 1). Moreover, to the best of our knowledge, no study has used decoding strategies to control variability in conceptual modeling tasks. The combination of factorial analysis for prompt design and different decoding mechanisms is thus a unique aspect of our methodology to enable a fine-grained understanding and control of how LLMs construct and vary conceptual representations.

## Materials and methods

### Three typical algorithms to combine causal maps

A causal map consists of nodes with labels that can be measured with ‘more’ or ‘less’. For example, (more/less) ‘healthy eating’ or (more/less) ‘self esteem’ would be appropriate labels, whereas ‘weather’ or ‘nutrition’ would not. Nodes are connected by edges. While edges are normally typed to encode causation, our focus is to *guide LLMs in identifying potential new edges and labeled nodes* that could join two disjoint maps. Additional prompts could be used to check the direction and type of such proposed edges, which is beyond the scope of this paper. This task of *model matching* has been one of the most common uses of LLMs in the recent literature (Table 1), thus we selected it to exemplify the importance of a statistical analysis focused on interactions and variability. We perform this task using three algorithms presented in the next subsections (Fig 4). The implementations of the algorithms are provided in our repository along with the data used for our experiments. Short illustrated examples of their main steps are also provided in **Supplementary** **S1 Appendix**.

**Fig 4:**
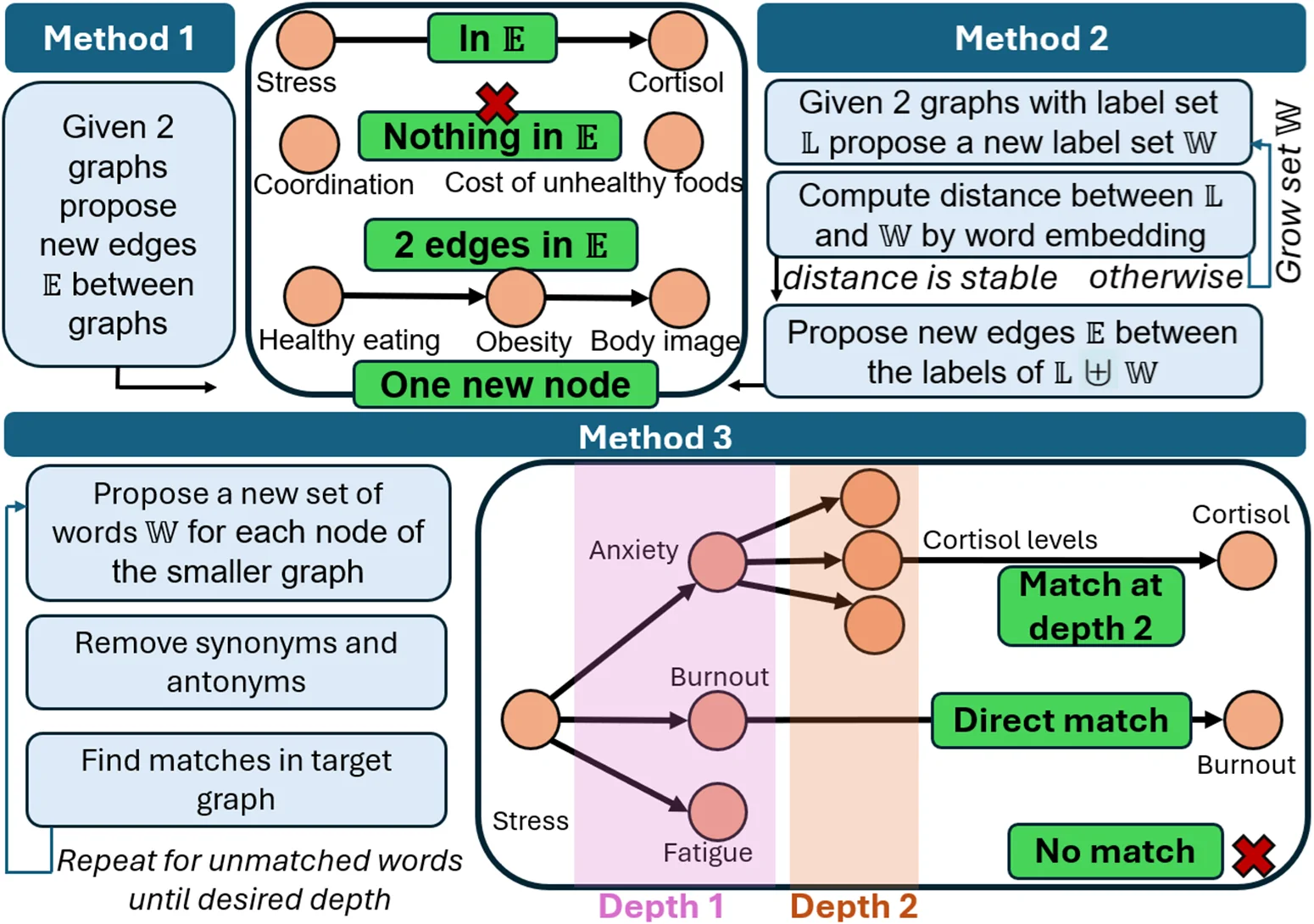
Our three algorithms to combine causal maps exemplify typical approaches in prompt engineering and Natural Language Generation, such as word embeddings. Nodes on the left-hand side come from one map and they must be matched with nodes on the right-hand side from another map. While edges are not displayed here, they are used by algorithms 1 and 2 to avoid proposing already existing connections.

### Algorithm 1: Direct combination

The overall goal of the first algorithm is to *directly* find new edges that link the nodes of two disjoint graphs. It captures a common approach whereby two conceptual models are given and the LLM is tasked with linking them. *The first prompt* provides the two models and asks for the links, and a follow-up prompt encourages the LLM to verify these links. The first prompt has a static part that Ⓐ gives a role, Ⓑ states the task, and Ⓒ imposes constraints to accomplish the task. The first prompt also has dynamic parts which can be present or absent, thus corresponding to a factorial design as exemplified in Fig 3. The dynamic parts are: whether the input is ① passed using an adjacency or list notation, and whether it is represented ② using tags or arrays (Fig 5; top-left); whether we provide ③ a task explanation, ④ an example, or ⑤ a counter-example. The prompt below illustrates the case of an adjacency notation, stored using arrays, with explanation, with an example, and with a counter-example. Note that the circles 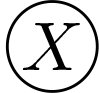 are not part of the prompt: they are only provided for the convenience of the reader to identify each of the static and dynamic elements. A circle 
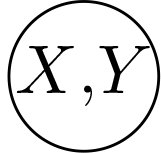
 indicates that the content reflects *both* options *X* and *Y*; for instance, 
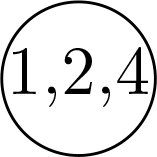
 reflects that the example is provided using a specific notation and representation.

**Fig 5:**
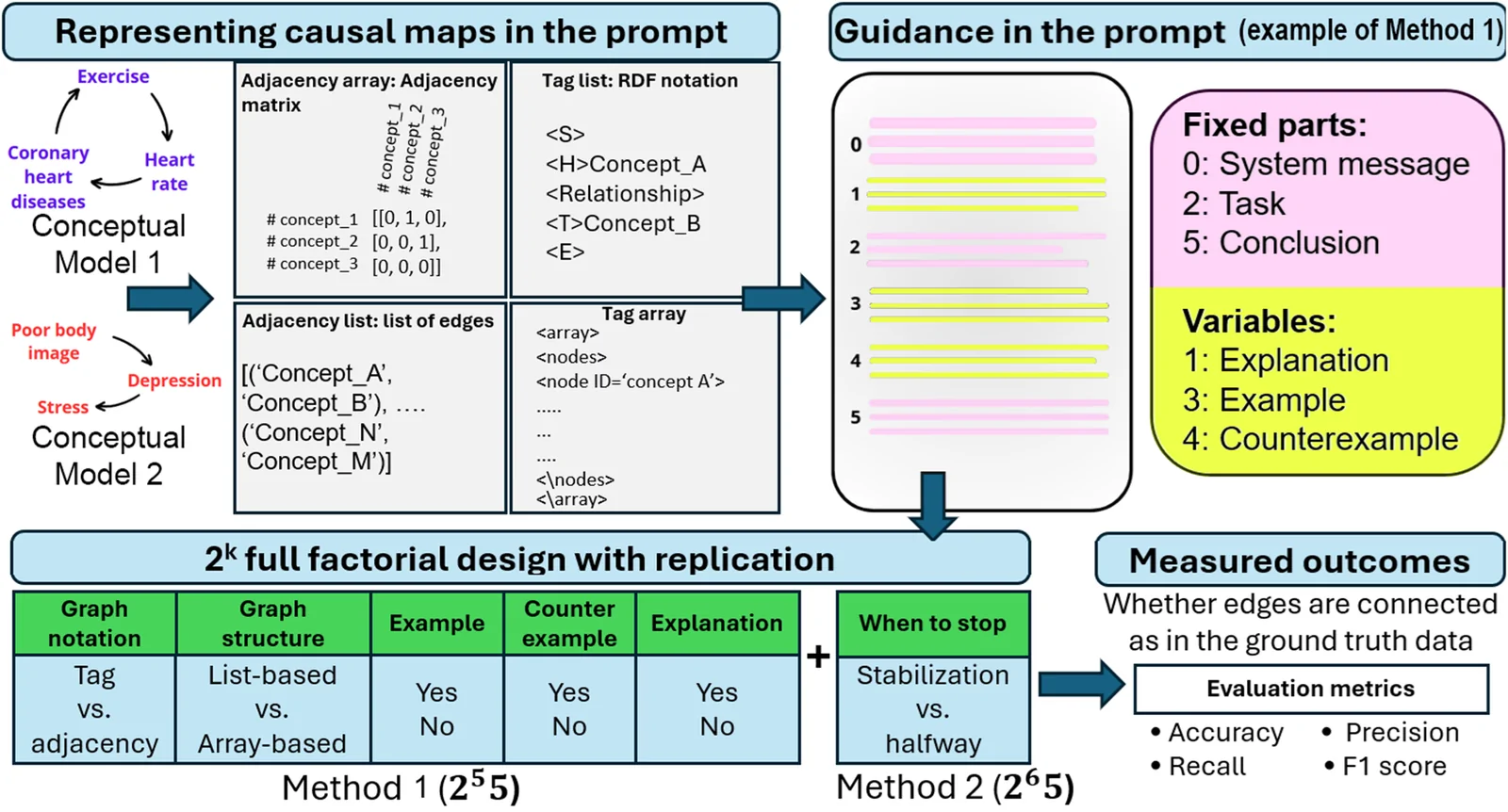
Our comprehensive factorial design accounts for changes in the textual representation of the conceptual models along with the guidance provided to the LLM. *This high-resolution figure can be zoomed in for details*.

**Algorithm 1. Sample combination for the first prompt. Dynamic elements are numbered 1–5.**

Ⓐ You are a helpful assistant who understands Knowledge Maps.

③ A knowledge map is a network consisting of nodes and edges. Nodes must have a clear meaning, such that we can interpret having ‘more’ or ‘’less’ of a node. Edges represent the existence of a direct relation between two nodes.

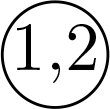
The knowledge map is encoded using a list of nodes and an associated adjacency matrix. The adjacency matrix is an n*n square matrix that represents whether each edge exists. In the matrix, each row and each column corresponds to a node. Rows and columns come in the same order as the list of nodes. A relation between node A and node B is represented as a 1 in the row corresponding to A and the column corresponding to B.

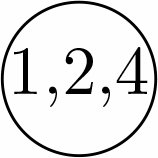
Here is an example of a desired output for your task. In knowledge map 1, we have the list of nodes [’capacity to hire’,’bad employees’,’good reputation’] and the associated adjacency matrix [[0,1,0],[0,0,1],[1,0,0]]. In knowledge map 2, we have the list of nodes [’work motivation’,’productivity’,’financial growth’] and the associated adjacency matrix [[0,1,0],[0,0,1],[0,0,0]]. In this example, you could recommend 3 new links:’quality of managers’ with’work motivation’,’productivity’ with’good reputation’ and’bad employees’ with’quality of managers’. These links implicitly create 1 new node:’quality of managers’. Therefore, this is the expected output: [(’quality of managers’,’work motivation’), (’productivity’,’good reputation’), (’bad employees’,’quality of managers’)].

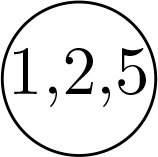
Here is an example of a bad output that we do not want to see. In knowledge map 1, we have the list of nodes [’capacity to hire’,’bad employees’,’good reputation’] and the associated adjacency matrix [[0,1,0],[0,0,1],[1,0,0]]. In knowledge map 2, we have the list of nodes [’work motivation’,’productivity’,’financial growth’] and the associated adjacency matrix [[0,1,0],[0,0,1],[0,0,0]]. A bad output would be: [(’moon’,’bad employees’)]. The error is the recommended link between’moon’ and’bad employees’. Adding the node’moon’ would be incorrect since it has no relationship with the other nodes. The proposed link does not represent a true causal relationship.

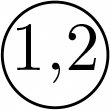
You will get two inputs: Knowledge map 1: <<data goes here>>

Knowledge map 2: <<data goes here>>

Ⓑ Your task is to recommend more links between the two maps. These links can use new nodes. Do not suggest links that are already in the maps. Do not suggest links between nodes of the same map. Return the recommended links as a list of edges in the format [(A, Z), ..., (X, D)].

Ⓒ Your output must only be the list of proposed edges. Do not repeat any instructions I have given you and do not add unnecessary words or phrases.

As a result of this prompt, we have a list of *potential* edges, which may also include new nodes. The *second prompt* (next page) validates these edges by asking the LLM whether it really believes that there is such a relation between concepts. Chain-of-Verification (CoVE) is a technique in prompt engineering where the reasoning process involves verifying the claims made in an initial response [67]. That is, the first prompt asks for a solution and the second prompt reduces the likelihood of errors by verifying the solution. This approach illustrates the use of ‘self-criticism’ in LLMs [68].

**Algorithm 1. The second prompt uses Chain-of-Verification.**

Return whether a causal relationship exists between the source and target concepts for each pair in a list. For example, given [(‘smoking’, ‘cancer’), (‘ice cream sales’, ‘shark attacks’)], return [‘Y’, ‘N’] with no other text.

Since the algorithm involves five binary factors and we run it five times to capture the effect of randomness, this algorithm is executed through a $2^{5}\times 5$ full factorial design with replication (see section).

### Algorithm 2: Grow candidate links through creative thinking and word embeddings

Algorithm 1 directly recommends (and then validates) edges, which *may* involve new nodes. However, combining conceptual models often calls for the identification of bridging concepts (conceptual integration) or the realization that concepts using different terms are equivalent (perceptual integration). Algorithm 2 operates in *two phases*: first we iteratively derive a set of related concepts (Algorithm 2 lines 1–8), and then we look for edges (Algorithm 2 lines 9–12). The first phase combines the labels of the two input graphs and asks for a list 𝕃 of five new related concepts. This leverages the ‘creative’ ability of LLMs to expand a causal model, as demonstrated by tools such as CausalMapper [69]. As discussed in [70], the LLM produces two types of creativity: creative *form* in using a different language (thus supporting perceptual integration) and creative *substance* when proposing novel ideas (thus helping with conceptual integration). We compute the distance between the suggested concepts 𝕃 and the original ones through embeddings (specified in the subsequent section on case studies) and computing cosine distance. Initially, the distance is maximal as only some of the words in 𝕃 align with the content of the graphs. We gradually add new concepts, five at a time, until the distance is stable. This indicates that we have exhausted the LLM’s ability to think creatively within our problem space. Then, we move onto the second phase by identifying all possible edges (in a similar manner to Algorithm 1). Some of these edges may already be present in the conceptual models, so they do not need to be considered. In line with Algorithm 1, we finalize the edges by asking for confirmation through chain-of-verification. Overall, the design of this algorithm emphasizes the identification of bridging concepts through creative thinking.

**Algorithm 2 Label Expansion and Edge Suggestion Algorithm**

**Require:** Two conceptual models with a set of labels 𝕃

**Ensure:** A set of new edges linking concepts of the two models

1:  Initialize a set 𝕎 of labels to be empty.

2:  Initialize distance *d*_previous_ to 0.

3:  Initialize distance *d*_current_ to 0.

4:  **repeat**

5:   Set *d*_previous_ to *d*_current_

6:   ***Prompt*** the LLM to generate 5 new labels related to 𝕃 and add them to 𝕎.

7:   Using text embeddings, compute the cosine distance *d*_current_ between 𝕎 and 𝕃.

8:  **until** $|d_{\text{current }}-d_{\text{previous}}|\leq 0.01$

9:  ***Prompt*** the LLM to suggest edges 𝔼 that directly link concepts in 𝕎⊎𝕃.

10:  Delete from 𝔼 all edges that already existed in either conceptual model

11:  Use a domain-specific thesaurus to convert synonyms and antonyms within 𝔼 into a base form

12:  ***Prompt*** the LLM using chain-of-verification to only keep edges in 𝔼 that pass validation

This algorithm uses the same binary factors as the first prompt (although the actual *prompt* is different since we generate word lists rather than edges) on notation (adjacency/list), representation (array/tag), and whether to have an explanation/example/counter-example. In addition, this algorithm has a parameter to decide when to stop growing the list of candidate words (Algorithm 2 line 8) thus we introduce a sixth binary factor to quantify the effect of this parameter, between stopping upon stabilization based on a 1% consecutive difference in cosine distance or stopping half as early. Overall, this produces a $2^{6}\times 5$ design.

### Algorithm 3: Tree-based approach for indirect matches

The second algorithm creates a ‘bag’ of new words that can be used to bridge the two conceptual models. The LLM may run out of creativity when it cannot find an idea that *directly* relates to a concept in the models. The third algorithm goes further into looking for bridges by deriving a *tree* of candidate ideas from one model (the ‘source’), thus aiming to reach the other model (the ‘target’) through *multiple* intermediates. Each concept of the source model is expanded into multiple candidate words. If a word is matched with the target graph, then we stop generating candidates; otherwise, unmatched words are further expanded until a maximum depth is reached. We vary four factors in the prompt, which are set to binary values to keep the design of experiments consistent: ① whether to include an example, ② whether to use a counter-example, ③ the number of candidate words per source label (3 vs. 5), and ④ the maximum depth (1 vs. 2). One of these combinations is shown in the prompt below; note that the depth is handled by *repeatedly* applying the prompt to build a tree rather than within a single prompt. This yields a $2^{4}\times 5$ design given the 5 replications. Unlike the first two algorithms, this tree-based procedure intentionally favors broad exploration. Because unmatched concepts are repeatedly expanded, a plausible but incorrect suggestion at one depth can generate many additional candidates at the next, leading to a combinatorial growth of false positives. Accordingly, we interpret algorithm 3 primarily through recall, i.e., its ability to recover true cross-graph links, rather than through precision- and accuracy-based summaries that would be dominated by this exploratory behavior.

**Algorithm 3. Sample prompt for the tree-based approach. Dynamic elements are numbered 1–4.**

You are a helpful assistant who can creatively suggest relevant ideas.

Your input is a set of concept names. All concept names must have a clear meaning, such that we can interpret having ‘more’ or ‘less’ of a concept.

Your input is the following list of concept names: <<data goes here>>

③ Your task is to recommend 3 related concept names for each of the names in the input. Do not suggest names that are in the input. Your output must include the list of the 3 proposed names for each of the input names. Do not include any other text. Return your proposed names in a dictionary format {{‘A’: [‘B’, ‘C’, ‘D’], ‘E’: [‘F’, ‘G’, ‘H’], ..., ‘U’: [‘V’, ‘W’, ‘X’]}}.

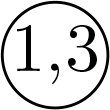
Here is an example of a desired output for your task. We have the list of concepts [‘capacity to hire’, ‘bad employees’, ‘good reputation’]. In this example, you could recommend these 9 new concepts: ‘employment potential’, ‘hiring capability’, ‘staffing ability’, ‘underperformers’, ‘inefficient staff’, ‘problematic workers’, ‘positive image’, ‘favorable standing’, ‘high regard’. Therefore, this is the expected output: {{“capacity to hire”: [‘employment potential’, ‘hiring capability’, ‘staffing ability’], “bad employees”: [‘underperformers’, ‘inefficient staff’, ‘problematic workers’], “good reputation”: [‘positive image’, ‘favorable standing’, ‘high regard’]}}.

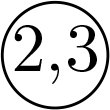
Here is an example of a bad output that we do not want to see. We have the list of nodes [‘capacity to hire’, ‘bad employees’, ‘good reputation’]. A bad output would be: {{“capacity to hire”: [‘moon’, ‘dog’, ‘thermodynamics’], “bad employees”: [‘swimming’, ‘red’, ‘happiness’], “good reputation”: [‘judo’, ‘canada’, ‘light’]}}. Adding the proposed concepts would be incorrect since they have no relationship with the concepts in the input.

Your output must only be the list of proposed concepts. Do not repeat any instructions I have given you and do not add unnecessary words or phrases.

### Baseline methods: WordNet-*k* Lexical matching and random-*k*

Although there is ample evidence that modelers are using LLMs for conceptual tasks (Table 1), it is useful to situate the performances of the algorithms defined in the previous section against simpler non-LLM alternatives. Such baselines can situate the extent to which LLMs perform beyond deterministic lexical matching or chance-level selection. To make this comparison fair, we provide two baseline methods (defined below) and match them to the number of cross-map connections (i.e., links between nodes from the first map and nodes from the second map) counted from the corresponding LLM output by the evaluator. That is, if an LLM output yields *k* scored cross-map connections for a given experimental condition, the baseline is also allowed to propose exactly *k* links. This prevents baselines from ‘gaming’ the metrics by proposing a large volume of potential links, thus focusing on the quality of their outputs. For example, because Algorithm 3 is evaluated through recall, a baseline that proposes many links could recover more valid links just by chance, even if most of its proposed links are incorrect.

Our first baseline is *WordNet-k*, which is formally described in Algorithm 4. It considers all possible links between nodes in the two input maps and ranks them using the Jaccard overlap in their labels. For each node, we create a set of words that includes the words already present in the label as well as related words obtained from WordNet. We keep the original words for the fairness of comparison (the LLM algorithms also receive the original node labels) and because many labels in conceptual models consist of several words that may not appear as a single entry in WordNet. For every possible link between the two input maps, we compare the two sets of words and compute the Jaccard overlap score, which is higher when they share more terms. The baseline then returns the *k* highest-scoring links, where *k* is the number of scored cross-map connections counted from the corresponding LLM output. The second baseline is *Random-k*, which is defined in Algorithm 5. It uses the same set of possible links between the two input models, but it does not use labels, WordNet, or any other information to rank them. Instead, it selects *k* links uniformly at random. Because random selection can vary from one draw to another, we repeat Random-*k* five times for each matched value of *k*. To avoid any confusion in the remainder of this article, we refer to the baseline algorithms as ‘the baselines’ and to the three LLM algorithms (the focus of this study) as ‘the algorithms’.

**Algorithm 4 WordNet-*k* Lexical Matching Baseline**

**Require:** Two conceptual models with label sets ${𝕃}_{1}$ and ${𝕃}_{2}$; a WordNet lexicon Ω; number *k* of scored cross-map connections counted from the corresponding LLM output

**Ensure:** A set ${𝔼}_{k}$ of *k* proposed links between concepts of the two models

1:  Initialize a scored list ℂ of candidate links to be empty. *// stores each possible link and its score*

2:  **for all** label $\ell \in {𝕃}_{1}⊎{𝕃}_{2}$ **do**

3:  ${𝕋}_{\ell }\leftarrow \text{Tokenize}(\ell )\cup \text{WordNetTerms}(\ell ,\Omega )$ *// keep original words in the label and add retrieved terms (e.g., synonyms, lexical variants) from the lexicon*

4:  **for all** label $u\in {𝕃}_{1}$ **do**

5:   **for all** label $v\in {𝕃}_{2}$ **do**

6:    **if** ${𝕋}_{u}\cup {𝕋}_{v}=\emptyset$ **then**

7:      s(u,v)←0 *// no available terms to compare*

8:    **else**

9:     $s(u,v)\leftarrow \frac{\left|{𝕋}_{u}\cap {𝕋}_{v}\right|}{\left|{𝕋}_{u}\cup {𝕋}_{v}\right|}$ *// Jaccard overlap between term sets*

10:   Add (s(u,v),(u,v)) to ℂ. *// (u,v) is a possible link between the two models*

11: Sort ℂ in decreasing order of *s*(*u*,*v*). *// highest lexical-semantic overlap first*

12: ${𝔼}_{k}\leftarrow$ the first min(k,|ℂ|) links in ℂ. *// match the number of scored cross-map connections counted from the LLM output*

13: **return** ${𝔼}_{k}$

**Algorithm 5 Random-*k* Baseline**

**Require:** Two conceptual models with label sets ${𝕃}_{1}$ and ${𝕃}_{2}$; number *k* of evaluated links counted from the corresponding LLM output

**Ensure:** A set ${𝔼}_{k}$ of *k* randomly proposed links between concepts of the two models

1:  Initialize a set ℂ of candidate links to be empty.    *// stores all possible links between the two models*

2:  **for all** label $u\in {𝕃}_{1}$ **do**

3:   **for all** label $v\in {𝕃}_{2}$ **do**

4:    **if** u≠v **then**

5:     Add (*u*,*v*) to ℂ.       *// (u,v) is an admissible link between the two models*

6: ${𝔼}_{k}\leftarrow$ a uniform random sample of min(k,|ℂ|) links from ℂ.   *// match the number of evaluated links counted from the corresponding LLM output*

7: **return** ${𝔼}_{k}$   *// This algorithm is run multiple times. Edge sets can differ each time.*

### Case study and analyses

#### Study data, software, and evaluation.

Our goal is to quantify the effect of prompt elements and randomness onto the ability of an LLM to combine conceptual models. That is, we focus on *decomposing* the performance through algorithms that represent various strategies used in the literature, rather than maximizing the performance by developing a groundbreaking new algorithm. To estimate the performance, we need a *ground truth* dataset that tells us whether different (small) conceptual models were correctly linked into a (larger) aggregate. Preferentially, each of the small constituent models should cover a *different facet* of the domain, thus illustrating the real-world challenge of combining the perspectives of experts originating from different fields. In addition, the domain of this dataset should be entirely *unrelated to the examples and counter-examples* used in our prompt, to avoid unintentionally biasing performances. Finally, to support replicability, the model should be *openly accessible*. For replicability, Table 2 lists the libraries that support our implementation along with the versions.

**Table 2:** Libraries used to generate our results and replicate our work.

Library	Version	Purpose
accelerate	1.13.0	Optimize execution and resource usage of local transformer models.
anthropic	0.86.0	Chat completions via the Anthropic API.
matplotlib	3.10.8	Data visualization and generation of analysis plots.
mistral-common	1.10.0	Tokenizers and utility functions for Mistral models.
mistralai	2.1.2	Client for the Mistral AI API.
networkx	3.6.1	Create the graphs used for evaluation.
numpy	2.4.3	Array operations supporting the factorial analysis.
openai	2.29.0	Embeddings and chat completions via OpenRouter.
pandas	3.0.1	Data manipulation and analysis.
pyyaml	6.0.3	Parsing and handling YAML configuration files.
scipy	1.17.1	Statistical analysis.
torch	2.11.0	PyTorch backend for executing local transformer models.
transformers	5.4.0	Executing local LLMs and computing text embeddings via Hugging Face.

### Data preparation and response variables

For Algorithms 1 and 2, the original graph supplied the ground-truth for subgraph connections. We combined the generated edges with the two input subgraphs and counted a connection as present when an undirected path existed between the corresponding nodes. Paths through newly generated intermediate nodes were allowed. We then counted true positives, false positives, true negatives, and false negatives over all possible cross-subgraph node pairs and computed accuracy, precision, recall, and F1 score. For Algorithm 3, we parsed the source, target, mother, and generated edge lists and computed recall as the proportion of source–target pairs connected in the mother graph that were also connected after adding the generated edges.

For Algorithm 2, generated terms were mapped through the fixed synonym–antonym thesaurus before duplicate filtering and evaluation. Binary prompt factors were represented by their low and high levels using −1 and + 1. F1 score, accuracy, precision, and recall were computed for Algorithms 1 and 2, and recall for Algorithm 3.

To evaluate performances, we frame the combination of models as a *binary classification* (Fig 6): the algorithm suggests whether to include certain edges and concepts, and the ground-truth tells us whether they should have been included. As a result, we can build a confusion matrix consisting of true positives, false positives, true negatives, and false negatives (Fig 6; bottom left). Since the LLM can propose *new nodes*, an edge is a true positive if there is a *path* (which may include new intermediate nodes) between two connected nodes in the ground truth map. Performance measures for this binary classification are derived from the matrix, thus we use precision, recall, and accuracy (Fig 6; bottom right).

**Fig 6:**
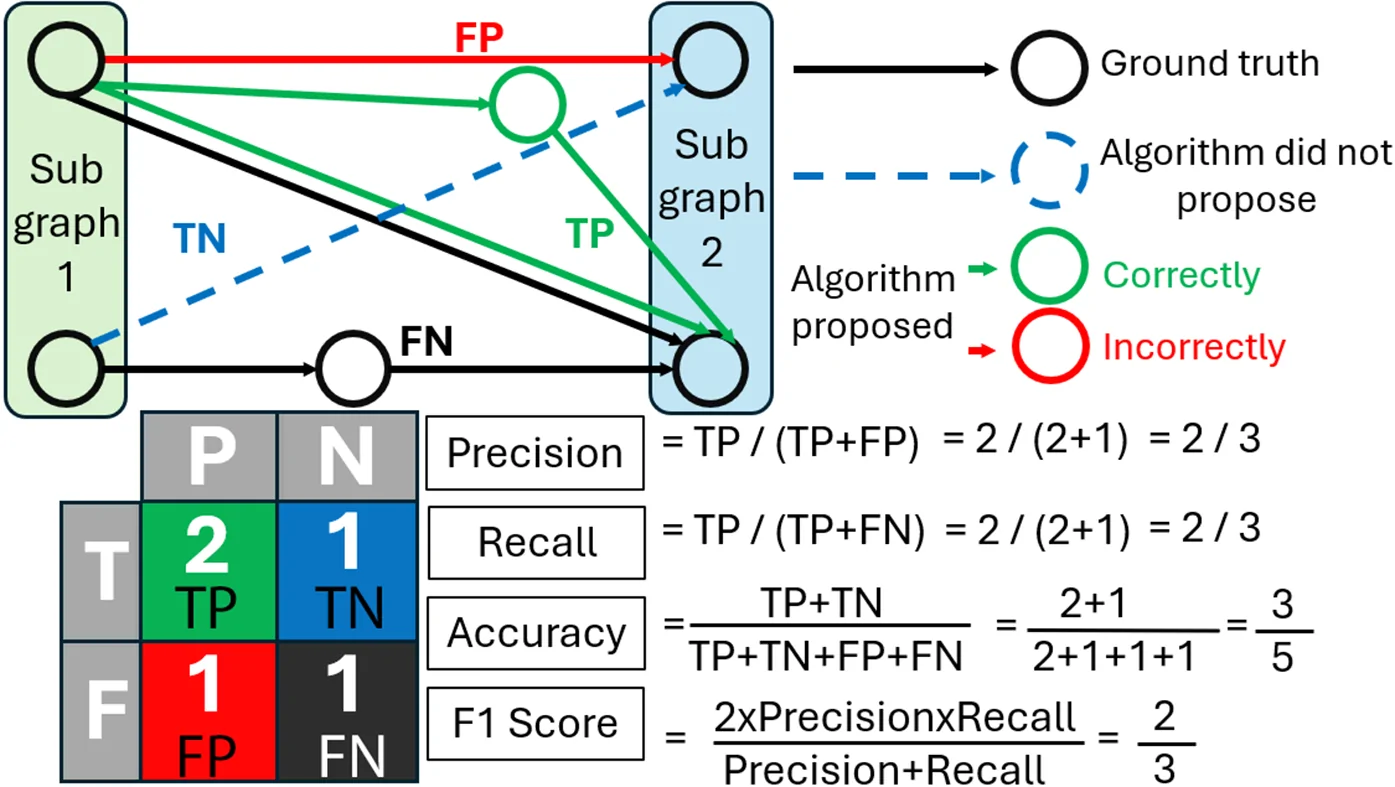
Each algorithm was used to combine a pair of sub-graphs originating from a larger causal map. The map provides the ground truth: it tells us which connections should have been made by the algorithm between the sub-graphs. The confusion matrix (bottom left) records whether an algorithm proposes a connection or not, and whether it existed in the ground truth or not. This yields four standard measures from binary classifications: precision, recall, accuracy, and F1 score.

We performed two series of experiments. First, we used three well-known LLMs (DeepSeek, Gemini, and GPT), each with two successive versions. This allows us to capture both architectural and generational effects on variability. The design of these experiments is detailed in the next section. Second, we used two open-weight LLMs (Qwen and Ministral) to directly control variability through the decoding strategy. These experiments are detailed in the subsequent section. Both sets of experiments used a causal map on obesity of 98 nodes and 176 edges developed in [71] and openly accessible at https://doi.org/10.17605/OSF.IO/KGDRU. We decomposed this model into three sub-models based on coherent themes reflecting areas of expertise: food consumption and production and the built environment (22 nodes, 20 edges); psychology, sociology, and well-being (14 nodes, 27 edges); and physiology, body weight, physical activity, and co-morbidities (43 nodes, 59 edges). Specifically for the second algorithm, we use a domain-specific thesaurus consisting of 12 entries for synonyms (e.g., ‘ability to engage in physical activity’ encompasses physical fitness, ‘mental well-being’ equates to ‘mental health’) and 8 entries for antonyms (e.g., ‘tiredness’ is the contrary of stamina and endurance). Each of our three algorithms was used to combine the three pairs of conceptual models. In addition, our second set of experiments examined several conceptual models for suicide prevention, thus broadening the application domains used in this study and reflecting the fact that several conceptual models can be produced on the same topic.

### Statistical analysis

We first quantify how variation is distributed across the experimental design using a factorial decomposition of variance. We analysed each metric separately. The reported factorial terms consisted of the main effects and all two-factor interactions; higher-order interactions were not included. This analysis measures the proportion of variation in performance metrics (accuracy, precision, recall) attributable to individual prompt elements, their interactions, and a residual term. It directly addresses whether performance differences can be explained by the factors we control, or whether a substantial share of variability remains unexplained.

Having identified which factors contribute to variability, we then assess whether these effects are systematic and interpretable. Specifically, we test whether prompt elements have consistent and *directional effects* on performance (i.e., whether including a given factor consistently increases or decreases a metric) by comparing paired settings (with/without a prompt element) across experimental conditions. To test directional effects, we compared the high and low levels of each binary factor while holding all other factorial settings and the repetition fixed. For each matched pair, we calculated the high-minus-low difference and applied a paired *t*-test. We report the mean difference, its 95% confidence interval, the paired *t* statistic, the raw *p*-value, and the Benjamini–Hochberg adjusted *q*-value. The 95% interval used $\bar{d}\pm 1.96s_{d}/\sqrt{n}$, where *d* is the vector of paired differences. Benjamini–Hochberg correction was applied within each factor’s test family across graph-pair files and metrics; a test was counted as significant when q≤0.05.

We next examine the residual variability that remains after accounting for experimental factors. The key question at this stage is whether this residual reflects unstructured noise or structured differences in the outputs produced by the LLM. For output-level stability, generated edge lists were parsed into sets of non-empty, trimmed edge pairs. We computed pairwise Jaccard similarity, exact-match pair rate, the sample standard deviation of edge counts, and the union of edges across repeated runs. The breadth-expansion ratio was calculated by dividing the union edge count by the mean edge count. For replication sufficiency, we used the observed mean $\bar{y}$ and sample standard deviation *s* for each condition–metric cell. With relative half-width target *h* = 0.05, the estimated number of runs was $n_{required}=\left\lceil (zs/(h|\bar{y}|){)}^{2}\right\rceil$, subject to the observed number of runs. We used *z* = 1.645 for 90% and *z* = 1.96 for 95%. For Algorithm 3, we kept each directed graph-pair condition separate and used the five-run mean and sample standard deviation to estimate the total number of repetitions needed for a 95% confidence interval with a 5% relative half-width. Zero-mean cells were treated as not estimable under this relative-error target.

### Variance across Prompts and Architectures: DeepSeek, Gemini, and GPT

The first set of experiments assesses whether variation in conceptual modeling performance can be explained by controllable factors (e.g., prompt design and LLM architecture) or whether it is dominated by residual variability intrinsic to LLMs. In particular, we seek to determine not only how much variation exists, but also whether it reflects meaningful, systematic effects or largely unexplained fluctuations. For the convenience of the reader, supplemental Fig S1 provides a detailed overview of the research questions, data, and analyzes that are detailed below. The first experiment series applies the statistical procedures described above to the replicated prompt designs.

To this end, we conduct a full factorial Design of Experiments with replication (Fig 5) for each of the three algorithms described above. Each algorithm is evaluated across combinations of binary prompt elements (e.g., inclusion of examples, counter-examples, explanations, and representation choices), yielding a $2^{k}$ design, and each experimental condition is repeated five times to capture variability in LLM outputs. These experiments are performed across three frontier LLM families (DeepSeek, Gemini, GPT), with two versions per family, allowing us to assess both cross-model and within-family (version) effects. The DeepSeek Chat v3.0324 and v3.1 models were chosen to examine how incremental fine-tuning within the same family influences output consistency and reasoning, as DeepSeek’s updates often involve modifications to attention routing and optimization objectives rather than scale. The Gemini 2.0 Flash and Gemini 2.5 Pro models provide a contrast between a latency-optimized, lightweight configuration and a larger, reasoning-oriented variant within Google’s multimodal architecture, thereby revealing the trade-offs between efficiency and conceptual fidelity. Finally, GPT-4o and GPT-5 represent OpenAI’s flagship general-purpose models across major generations, allowing us to assess how architectural and training innovations in the most widely adopted LLM line affect reproducibility and variability in conceptual modeling tasks. Since this set of experiments evaluates the combination of conceptual modeling under commonly available proprietary services, we used OpenAI’s text-embedding-3-large as embedding for Algorithm 2. This embedding is OpenAI’s most capable embedding model for English and non-English text similarity tasks [72]. The API access windows for these models are reported in Table 3.

**Table 3:** API access windows for the six proprietary models. Timestamps are in UTC and correspond to the first and last logged generation.

Model	First API timestamp (UTC)	Last API timestamp (UTC)
DeepSeek Chat v3.0324	2025-06-14 06:45:54.723	2025-08-27 19:40:48.966
DeepSeek Chat v3.1	2025-08-23 07:29:28.070	2025-10-12 12:29:58.469
Gemini 2.0 Flash	2025-06-15 07:42:18.375	2025-08-30 13:42:38.325
Gemini 2.5 Pro	2025-08-23 06:08:25.853	2025-08-27 17:45:45.680
GPT-4o	2025-08-23 07:46:15.536	2025-08-27 16:46:16.289
GPT-5	2025-08-23 07:36:59.735	2025-08-31 06:14:58.993

Finally, to contextualize the use of LLMs, we assess whether their performance provides value beyond simpler deterministic approaches. We include two non-LLM baselines to determine whether observed results can be achieved through lexical or random matching rather than semantic inference. We first implement a WordNet-based lexical baseline, which expands node labels using WordNet, computes lexical-semantic overlap between candidate node pairs across sub-graphs, ranks admissible pairs, and returns the top *k* edges. To ensure comparability with LLM outputs, *k* is matched to the number of edges produced in each run, so that evaluation focuses on the quality of selected edges rather than differences in output size. We additionally include a random-*k* baseline, which selects *k* edges uniformly at random, providing a lower-bound reference for chance-level performance.

### Decoding control and cross-domain generalization: Qwen and ministral

The first set of experiments aforementioned seeks to identify substantial residual variability that cannot be fully explained by prompt design or model choice. The key follow-up question is whether this residual reflects intrinsic stochasticity of the models, or whether it arises from specific mechanisms of inference that can be controlled. Well-known models such as OpenAI’s GPT limit the ability to isolate how outputs are generated as they mostly expose sampling-based decoding. Using open-weight LLMs that let us control decoding mechanisms, we thus seek to refine the analysis of variability by introducing explicit control over the decoding process, and to determine whether variability previously attributed to the LLM can be explained by differences in decoding mechanisms. For the convenience of the reader, supplemental Fig S2 summarizes the design of these experiments.

To address this question, we use two open-weight LLMs: Qwen3.5-9B (released Mar 2026) and Ministral-3-8B-Instruct-2512 (released Dec 2025). Both models are compatible with Transformers, which gives us access to different decoding methods. Because the open-weight experiments are intended to isolate decoding mechanisms under local control, using a proprietary embedding API in Algorithm 2 would have reintroduced an external black-box component into the experiments designed to reduce such dependencies. We thus used Qwen3-Embedding-0.6B for Algorithm 2, as it has a strong public benchmark performance [73], and its suitability for efficient local deployment as a lightweight semantic embedding has been noted in other recent studies [74]. For example, Niu *et al.* used OpenAI’s text-embedding-3-large as the proprietary solution and Qwen3-Embedding-0.6B as the open-source solution [75].

The design of experiments described in the previous section is expanded: in addition to the prompt factors, we introduce decoding (described in section Potential Sources of Variability in LLM Outputs) as an explicit experimental factor. Specifically, we compare greedy decoding, beam search with multiple beam widths, and contrastive decoding with different penalty parameters. Each experimental condition is repeated five times to capture variability across runs. Our experiments first extend the factorial decomposition of variance by adding decoding as factors. Consequently, the analysis quantifies the contribution of prompt elements, decoding strategies, and their interactions to performance variability across accuracy, precision, and recall. Variability previously attributed to the LLM as a whole can thus be decomposed more precisely into identifiable components associated with decoding choices.

Having established the role of decoding, we then examine whether these effects are specific to a given application domain or reflect more general properties of conceptual modeling with LLMs. To this end, we extend the experiments to conceptual models from a second domain, suicide prevention. Multiple maps are selected to represent diverse yet plausible conceptualizations of this domain, ensuring variation in both structure and semantics. This extension enables us to assess whether the effects of decoding and prompt design are consistent across domains with different levels of conceptual ambiguity and heterogeneity.

Finally, we use the Confidence Interval (CI) method to determine the desired number of replications. That is, for each experimental condition (e.g., with/without examples in the prompt, beam search vs contrastive decoding) and each performance metric (e.g., precision, recall), we estimate the number of runs required to achieve a target relative CI of 90% or 95% using the observed mean and variance. This analysis allows us to characterize the distribution of variability across experimental conditions and to identify the specific settings in which an LLM exhibits substantial variance.

## Results

### Overview

As described in the previous section, we used the three model-combining algorithms (representing the range of strategies available to modelers) with a factorial design (exemplified in Fig 3). The first set examines how much of the variation in performance can be explained by prompt design, model family, and version, and whether residual variability reflects noise or structured differences in outputs. The second set uses open-weight models to introduce explicit control over decoding, assess its role in variability, and evaluate the generality of these effects across domains. Our results are organized on these two sets of experiments accordingly. Note that variability is never an artifact of invalid outputs in our case: across all raw generations, we observed neither malformed nor empty responses. This section ends with a comparison of results from LLMs with two non-LLM baselines.

### Variance across prompts and architectures

We performed a $2^{k}r$ factorial design with replications for each of the three algorithms. We compare two analyses of the resulting data: one that correctly includes an error term to isolate the effect of stochastic variation, and one that attributes all observed variation to controllable prompt elements. This comparison reveals how ignoring inherent variability can lead to an overestimation of factor effects and create an illusion of control. Before going through the decomposition of variance in statistical tables, Fig 7 summarizes the means and distributions across metrics (accuracy, precision, recall), conceptual combination algorithms, and LLMs. We see that accuracy is generally high and in a narrow range across LLMs for Algorithms 1 and 2, whereas precision and recall have a broader distribution that indicates wider differences in the links that were generated by the combination algorithms. Algorithm 3 stands out with a low recall. To analyze these patterns, the following tables examine each algorithm in turns.

**Fig 7:**
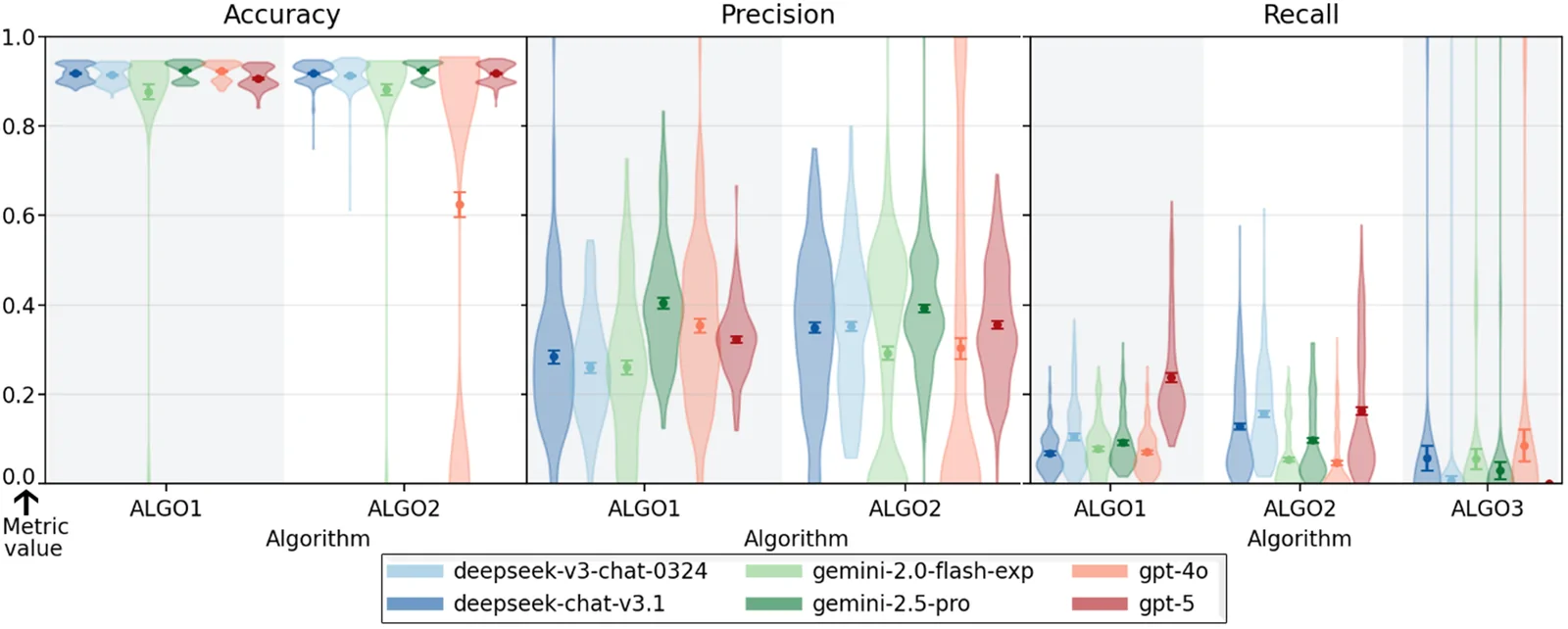
Results from the first set of experiments across combination of algorithms, LLMs, and metric. Each violin plot shows the empirical distribution of a performance metric for one algorithm and LLM. The marker and error bar indicate the mean and its 95% confidence interval. Narrow plots correspond to stable results across repetitions, whereas wider distributions indicate greater heterogeneity in outcomes. Algorithm 3 is only shown for recall because this is the only metric considered for that algorithm.

Algorithm 1 captures the simplest approach to combining models by direct combination. In this case, modelers have some control over the output: across all LLMs, performances are primarily driven by prompt elements (Table 4). In particular, the format (array or list, tags or adjacency) and the use of examples are important contributors. However, the impact is mediated by the LLM and the version. Both versions of DeepSeek show that formatting has a strong effect either directly (e.g., almost 24% of recall is attributable to the use of arrays vs. list in DeepSeek 3.1) or through interactions (e.g., array vs. list and tags vs. adjacency are involved in 47% of precision for DeepSeek 3 and 55% for DeepSeek 3.1). However, differences across the two versions of DeepSeek show that architectural improvements can alter the reactions to these prompt elements. DeepSeek 3 reacts to the choice of array vs. list through interactive effects whereas DeepSeek 3.1 is directly sensitive to this choice. We have similar observations from Gemini. In Gemini, the choice of array vs. list representations was a high contributor to recall, and more so in the latest version of the model than its predecessor. In a similar manner, the representation as tags or adjacency matrix strongly shaped the accuracy in the earlier model, but it was only captured through interactive effects in the newer version. While some of the variance in results from Gemini 2 was attributable to stochastic noise, this was limited to 1.24% to 3.79% and it did not change the conclusions about the importance of individual factors or interactions. Lastly, GPT behaves differently as its recall is always highly sensitive to the use of examples (21.88% for GPT-4o and 34.96% for GPT-5), and it is also much more reactive to task explanations and counterexamples. While the earlier GPT-4o reacted to data format, the newer GPT-5 only reacts to examples, explanations, and counterexamples. Overall, the analysis from Algorithm 1 demonstrates that prompt composition exerts measurable effects, but these effects depend on the LLM and its version. This provides *empirical evidence that model evolution alone can change experimental outcomes.*

**Table 4:** Factors that account for at least 10% of the variance in results across any of the measured outcomes in Algorithm 1, for the base or latest version of each of the three LLMs. When the inclusion of the error term modifies the breakdown of variance, we show the term *without* error vs. the term accounting for errors.

		*Base (DeepSeek 3, Gemini 2, GPT-4o)*			*Latest (DeepSeek 3.1, Gemini 2.5, GPT-5)*		
LLM	Feature	accuracy	recall	precision	accuracy	recall	precision
DeepSeek	Array/List				6.25	23.73	12.32
	Array/List&Tag/Adjacency	10.58	15.55	30.29	10.02	17.61	19.70
	Example	8.41	15.63	7.93	19.82	11.55	3.33
	Example&Array/List				7.42	9.43	12.88
	Example&Counterexample				5.54	10.57	9.08
	Example&Tag/Adjacency				8.95	3.66	10.36
	Tag/Adjacency	18.13	2.94	8.57			
	Counterexample&Array/List	8.12	10.62	7.27			
	Explanation&Array/List	11.35	6.34	9.44	5.11	4.22	12.06
	Error term	0.00	0.00	0.00	0.00	0.00	0.00
Gemini	Array/List	3.45 vs 3.54	11.71 vs 11.93	7.79 vs 8.11	11.86	15.50	16.04
	Example	17.79 vs 18.12	0.65 vs 0.69	12.96 vs 13.11	20.37	16.15	9.59
	Example&Counterexample	4.48 vs 4.49	8.56 vs 9.05	10.14 vs 10.48			
	Example&Tag/Adjacency	8.93	6.42	10.34			
	Explanation	12.49	2.69	15.96			
	Explanation&Tag/Adjacency	10.40 vs 10.48	17.59 vs 18.51	9.31 vs 9.69			
	Tag/Adjacency	20.13 vs 20.57	17.41 vs 18.32	10.80 vs 11.11			
	Error term	1.24	3.79	2.70	0.00	0.00	0.00
GPT	Array/List&Tag/Adjacency	14.19	6.91	10.75 vs 10.77			
	Counterexample	1.78	10.03	5.40			
	Example	8.21	21.88	2.77	20.20	34.96	5.41
	Array/List	10.14	8.82	11.16			
	Explanation	11.69	5.61	6.72			
	Tag/Adjacency	6.30	9.81	17.84			
	Example&Array/List	8.52	3.72	10.75			
	Explanation&Counterexample	5.93	10.00	11.33	12.51	6.65	22.88
	Explanation&Tag/Adjacency	14.60	4.22	11.21			
	Error term	0.01	0.00	0.05	0.00	0.00	0.00

Algorithm 2, which relies on model expansion guided by convergence criteria, yields different patterns in the decomposition of variance (Table 5). Its unique ‘convergence’ parameter has a strong effect across the LLMs, although its importance once again depends on both the LLM and the version. This pattern is consistent with Fig 7, where Algorithm 2 retains high accuracy overall but exhibits a visibly broader spread on precision and recall than Algorithm 1. While convergence shapes precision to a similar extent between versions (e.g., 12.04% to 12.10% on DeepSeek, 21.88% to 22.40% on Gemini), there are noticeable fluctuations in recall (e.g., from 11.88% to 4.85% on DeepSeek, 30.10% to 7.44% on Gemini). As in Algorithm 1, we see that representation (array vs. list, tags vs. adjacency) influences the outcomes for DeepSeek and for Gemini, but results are different for GPT. In this case, the earlier GPT-4o did *not* react strongly to data format, but the newer GPT-5 does. *A hypothesis solely from the experimental results of Algorithm 1 could have been that GPT is able to understand the data regardless of how it is presented, but results from Algorithm 2 counter this hypothesis as the representation is a strong driver of recall* (over 25%). Noticeably, the latest GPT-5 has a large randomness effect, unlike its previous version or the other two LLMs. For example, randomness accounts for 16.59% of the variance in precision. Ignoring the effect of randomness would have given the illusion that representation plays a role in accuracy, whereas correctly accounting for randomness reveals that this role is much lower (8.54% without randomness vs. 1.02%). *This demonstrates that omitting randomness can artificially inflate the apparent impact of controllable prompt features.* In addition, it reveals that *prompt design and randomness interact*: the same prompt configuration can yield different attributions of importance depending on whether variability is properly accounted for.

**Table 5:** Factors that account for at least 10% of the variance in results across any of the measured outcomes in Algorithm 2, for the base or latest version of each of the three LLMs. When the inclusion of the error term modifies the breakdown of variance, we show the term *without* error vs. the term accounting for errors.

		*Base version*			*Latest version*		
LLM	Feature	accuracy	recall	precision	accuracy	recall	precision
DeepSeek	Array/List&Tag/Adjacency	2.43	11.88	12.04	7.19	4.85	12.10
	Convergence	12.42	23.74	8.91	7.75	10.11	2.11
	Example	20.60	8.33	37.98	23.29	8.81	18.76
	Example&Tag/Adjacency				8.06	10.19	10.58
	Tag/Adjacency				7.36	7.33	10.03
	Error term	0.00	0.00	0.00	0.00	0.00	0.00
Gemini	Convergence	10.31	30.10	21.88	20.97	7.44	22.40
	Counterexample	10.30	0.34	9.12			
	Explanation				13.49	0.87	9.84
	Array/List&Tag/Adjacency				0.35	13.90	0.07
	Example&Convergence				5.70	1.91	14.11
	Counterexample&Array/List	5.73	11.85	7.21			
	Explanation&Counterexample	17.23	1.83	4.27			
	Error term	0.00	0.00	0.00	0.00	0.00	0.00
GPT	Convergence	25.49	15.09	14.55	22.34 vs 27.54	5.58 vs 9.95	15.58 vs 21.09
	Example	28.14	19.77	24.84			
	Array/List&Tag/Adjacency				8.54 vs 1.02	25.35 vs 27.42	8.55 vs 9.01
	Explanation&Array/List				10.83 vs 14.05	3.29 vs 3.32	13.72 vs 15.46
	Explanation&Convergence	3.30	8.23	12.19			
	Error term	0.00	0.00	0.00	8.32	14.43	16.59

Finally, Algorithm 3 using an indirect or tree-based combination exhibits the clearest dominance of stochastic effects (Table 6). Here, recall variance is almost exclusively explained by the error term for nearly all LLMs and versions. Interestingly, the effect of randomness is increasing as we use the LLMs’ latest versions: the error term went from 84% to almost 96% of variance from DeepSeek v3 to v3.1, and from about 73% to over 81% between Gemini 2 and 2.5. Results were not available from GPT-4o as it failed the combination task. This analysis strongly demonstrates the benefits of accounting for randomness. When the error term is ignored, secondary features such as Depth, Number of Words, or interactions like Example and Counter-Example appear spuriously large, sometimes exceeding 20–30%. But when we control for randomness, these inflated values vanish and we realize that the prompt design and main algorithm parameter (the depth) provide very little, or almost no control. The finding that randomness plays such a strong role in Algorithm 3 but not in Algorithm 1 or Algorithm 2 may be due to the different design of Algorithm 3, that is, the multi-step reasoning. It is possible that this multi-step reasoning *amplifies* the intrinsic variability of LLMs, as *small stochastic deviations in early generations cascade through subsequent steps*, making outcomes highly irreproducible without extensive replication. This difference is also apparent in Fig 7, where the recall distributions for Algorithm 3 are concentrated near zero across frontier models but often display elongated upper tails, indicating that successful recoveries of true links occur only sporadically rather than at a stable moderate level.

**Table 6:** Factors that account for at least 10% of the variance in recall in Algorithm 3, for the base or latest version of each of the three LLMs.

			With error term	Without error term
DeepSeek	Base	Error term	84.28	
		Counter-Example		16.49
		Counter-Example&Depth		16.49
		Counter-Example&Number of Words		16.49
		Depth		16.49
		Number of Words		16.49
		Number of Words&Depth		16.49
	Latest	Error term	95.88	
		Counter-Example&Number of Words		11.77
		Depth		49.45
		Number of Words		27.73
Gemini	Base	Error term	73.16	
		Counter-Example&Depth		20.88
		Number of Words		30.15
	Latest	Error term	81.21	
		Counter-Example		10.05
		Counter-Example&Number of Words		10.05
		Depth		12.81
		Example		16.73
		Example&Counter-Example		29.36
		Example&Depth		10.05
GPT	Latest	Error term	53.94	
		Depth	29.42	63.87
		Example		15.43

The factorial decompositions above tell us how much variation is attributable to controllable prompt and model factors, and how much remains in the residual term. We next address two follow-up questions: whether the controllable factors have consistent directional effects on performance, and whether the remaining variability reflects unstructured noise or structured differences in the outputs.

The paired factor-level tests show that prompt elements can have systematic effects, but these effects are not equivalent to straightforward improvements (Table 7). For Algorithms 1 and 2, several factors retain many significant comparisons after Benjamini–Hochberg correction, which controls the false discovery rate when many related tests are performed. However, the signs of the mean differences show that these effects often involve tradeoffs across metrics. For example, the strongest factors for Algorithm 1 improve some metrics while reducing others, and Algorithm 2 shows a particularly strong effect of the Convergence factor but not of all prompt elements. Thus, prompt design can exert measurable control, but its effect depends on both the algorithm and the performance metric. Algorithm 3 differs sharply from the first two algorithms. Only Depth has any corrected significant comparisons, while Example, Counter-Example, and Number of Words show none (Table 7). This supports the conclusion from the factorial decomposition: Algorithm 3 offers little stable prompt-level control and is instead dominated by residual variability, with depth providing only a limited directional effect on recall.

**Table 7:** Aggregated paired factor-level tests by algorithm and factor. We report significant tests, share, accuracy, precision, and recall. Mean differences are shown for each metric, and significance counts report Benjamini-Hochberg corrected tests.

Alg.	Factor	Tests	Share	ΔAcc.	ΔPrec.	ΔRec.	Acc.	Prec.	Rec.
1	Array/List	29/54	53.7%	−.0013	−.0206	.0050	8/18	10/18	11/18
	Counterexample	18/54	33.3%	.0013	.0068	−.0006	4/18	6/18	8/18
	Example	37/54	68.5%	−.0023	.0319	−.0276	15/18	9/18	13/18
	Explanation	25/54	46.3%	.0087	.0207	−.0114	8/18	9/18	8/18
	Tag/Adjacency	35/54	64.8%	.0048	−.0168	.0062	10/18	12/18	13/18
2	Array/List	14/54	25.9%	−.0061	−.0158	−.0002	8/18	5/18	1/18
	Convergence	44/54	81.5%	.0324	−.0194	.0122	15/18	14/18	15/18
	Counterexample	23/54	42.6%	−.0104	−.0178	−.0065	9/18	7/18	7/18
	Example	31/54	57.4%	−.0469	−.0211	−.0083	10/18	11/18	10/18
	Explanation	21/54	38.9%	.0002	−.0055	−.0028	6/18	6/18	9/18
	Tag/Adjacency	23/54	42.6%	.0178	.0080	−.0015	9/18	7/18	7/18
3	Counterexample	0/6	.0%	N/A	N/A	−.0054	N/A	N/A	0/6
	Depth	2/6	33.3%	N/A	N/A	.0508	N/A	N/A	2/6
	Example	0/6	.0%	N/A	N/A	.0102	N/A	N/A	0/6
	#Words	0/6	.0%	N/A	N/A	.0160	N/A	N/A	0/6

We then examined whether the residual variability corresponds to small fluctuations around stable outputs or to qualitatively different generated structures. At the performance level, Algorithms 1 and 2 are almost perfectly stable under repetition: Algorithm 1 shows non-zero spread in only 2 of 576 accuracy conditions, 3 of 576 precision conditions, and 2 of 576 recall conditions, while Algorithm 2 varies in only 1 of 1152 conditions per metric. Algorithm 3 is different, with 53 of 288 recall conditions showing non-zero dispersion. The mean coefficient of variation is approximately $2.4\times 10^{-5}$ for Algorithm 1 accuracy and $1.5\times 10^{-5}$ for Algorithm 2 accuracy, but 1.9 for Algorithm 3 recall. The same pattern appears in the raw predicted edge sets: Algorithms 1 and 2 have mean pairwise Jaccard similarities of 0.9981 and 0.9996, exact-match pair rates of 0.9979 and 0.9991, and breadth-expansion ratios of 1.0039 and 1.0005, respectively. Algorithm 3 has a mean Jaccard similarity of only 0.0770, an exact-match rate of 0.0010, and a breadth-expansion ratio of 4.1321, indicating that repeated runs explore substantially different edge sets rather than minor perturbations of the same output.

The estimated replication budget was model-dependent. For Algorithm 3 Recall, 14 of 48 DeepSeek Chat v3.1 conditions varied across five runs, with a median estimated total of 7,684 runs among non-zero cells; for GPT-4o, 9 of 48 conditions varied, with a corresponding median of 5,283 runs. Across all six models, 53 of 288 conditions varied, so five repetitions should be treated as an initial screening budget rather than a universal standard.

### Variance across decoding controls and generalizability

The second set of experiments expands on the factorial analysis since we now vary the content of the prompts and the decoding approach. For example, in Algorithm 1, we had three pairs of subgraphs, 2^5^ prompt conditions, and 5 replications. When accounting for the 5 decoding conditions and the two open-weight LLMs, this produces $5\times 3\times 2^{5}\times 5\times 2=4,800$ runs. Across the three algorithms and their different prompt conditions, we thus performed 16,800 experiments to examine the implications of decoding methods and prompting.

Table 8 demonstrates that *decoding is not a neutral implementation detail*: it has a substantial effect on performance. However, there is no ‘universally best’ decoding as it depends on both the LLM and the algorithm. For Algorithm 1, Qwen performs best with beam search using two beams, whereas Ministral performs best with greedy decoding and is nearly perfect across all metrics. For Algorithm 2, the pattern reverses: Qwen performs best with greedy decoding, whereas Ministral performs best with beam search using two beams. Contrastive decoding with a high penalty (penalty_alpha=0.8) performs poorly across both LLMs and the first two algorithms, indicating that excessive pressure for diversity can severely degrade the generated links. Algorithm 3 remains difficult for both models: recall is low overall, although Qwen improves modestly with a beam search of six beams.

**Table 8:** Mean scores (%) by decoding condition, LLMs, and combination algorithms. Greedy has no parameter; beam search has num_beams (2 or 6); contrastive decoding (‘Ctras.’) has a penalty_alpha (0.2 or 0.8). Best results are bolded. The Runs column applies to both LLMs (e.g., they *each* had 480 runs for Alg 1 under greedy decoding).

Alg.	Decoding	Runs	Qwen				Ministral			
			Acc.	Prec.	Rec.	F1 score	Acc.	Prec.	Rec.	F1 score
1	Greedy	480	83.74	94.79	83.74	87.19	**95.31**	**100**	**95.31**	**97.31**
	Beams (2)	480	**88.45**	**96.25**	**88.45**	**90.69**	91.67	**100**	91.67	94.94
	Beams (6)	480	81.67	95.42	81.67	84.77	91.11	**100**	91.11	94.46
	Ctras. (.2)	480	84.56	94.79	84.56	87.70	92.52	96.88	92.52	94.17
	Ctras. (.8)	480	20.22	27.08	20.22	21.93	8.85	13.54	8.85	10.20
2	Greedy	960	**70.98**	**84.38**	**70.98**	**75.33**	70.89	85.94	70.89	76.22
	Beams (2)	960	62.29	79.69	62.29	67.73	**79.07**	**92.19**	**79.07**	**84.06**
	Beams (6)	960	57.77	68.75	57.77	60.90	67.23	84.38	67.23	73.02
	Ctras. (.2)	960	60.21	73.23	60.21	63.99	61.89	77.60	61.89	66.94
	Ctras. (.8)	960	5.78	6.77	5.78	6.04	0.43	1.04	0.43	0.60
3	Greedy	240	N/A	N/A	4.83	N/A	N/A	N/A	**1.89**	N/A
	Beams (2)	240	N/A	N/A	6.34	N/A	N/A	N/A	0.95	N/A
	Beams (6)	240	N/A	N/A	**8.33**	N/A	N/A	N/A	0.95	N/A
	Ctras. (.2)	240	N/A	N/A	0.00	N/A	N/A	N/A	0.95	N/A
	Ctras. (.8)	240	N/A	N/A	2.08	N/A	N/A	N/A	0.00	N/A

The variance decomposition in Table 9 explains why these mean differences should not be interpreted as complete control over the outputs. When the error term is retained, *residual variability is the largest contributor in most settings*, particularly for Algorithm 2 and for Algorithm 3, where the error term exceeds 65% for Qwen and 78% for Ministral recall. Decoding-related factors still matter: contrastive penalty and the beam-versus-contrastive distinction explain substantial variance for Algorithms 1 and 2. However, the comparison with the decomposition that omits the error term shows the same illusion of control observed in the previous set of experiments. Once residual variability is ignored, the apparent contribution of controllable decoding factors is inflated, and additional prompt factors appear above the reporting threshold for Algorithm 3. Thus, *even with open-weight models and explicit decoding control, variability cannot be fully attributed to user-controlled settings*. The error term remains central to interpreting LLM behavior.

**Table 9:** Percentage of variance explained by individual terms and contributions across metrics, LLMs, and algorithms. The error column indicates whether the decomposition appropriately retained the residual error term or ignored it. Only terms contributing at least 10% in variance are displayed here, while the full decomposition is provided in Supplementary Table S2.

Error	Alg.	Feature	Qwen			Ministral		
			Prec.	Acc.	Rec.	Prec.	Acc.	Rec.
Yes	1	Beam vs Contrastive	16.27	13.05	13.05	27.28	22.94	22.94
	1	Contrastive Penalty	30.62	25.33	25.33	47.22	48.47	48.47
	1	Error	45.89	54.81	54.81	17.32	19.86	19.86
	2	Beam vs Contrastive	–	–	–	22.11	19.80	19.80
	2	Contrastive Penalty	18.86	14.90	14.90	27.04	21.21	21.21
	2	Error	55.19	62.35	62.35	38.49	44.96	44.96
	3	Error	N/A	N/A	65.03	N/A	N/A	78.78
No	1	Beam vs Contrastive	30.06	28.89	28.89	33.00	28.63	28.63
	1	Contrastive Penalty	56.59	56.04	56.04	57.11	60.49	60.49
	2	Beam vs Contrastive	22.31	19.53	19.53	35.95	35.97	35.97
	2	Contrastive Penalty	42.08	39.58	39.58	43.96	38.53	38.53
	2	Greedy vs Beam/Contrastive	11.33	12.80	12.80	–	–	–
	3	Depth	N/A	N/A	–	N/A	N/A	20.49
	3	Example	N/A	N/A	18.57	N/A	N/A	20.49
	3	Example & Depth	N/A	N/A	–	N/A	N/A	20.49
	3	Example & Number of Words	N/A	N/A	12.63	N/A	N/A	–
	3	Number of Words	N/A	N/A	12.63	N/A	N/A	–

Because Algorithm 3 exhibited the largest residual variability in both sets of experiments, we use it as the focal case for testing whether our findings generalize beyond the obesity map. To avoid selecting a narrow second case, we drew from an open-access set of 15 suicide-prevention maps produced by subject matter experts [76] and selected three maps that maximize the coverage of distinct perspectives. We proceeded in four steps. First, we needed each selected map to be divisible into three usable subgraphs, since the experimental design compares links across subgraphs. We therefore analyzed how each map decomposed into three clusters through commonly used community detection algorithms (Girvan-Newman, spectral clustering, recursive Kernighan-Lin partitioning). Second, we assessed the clusters to ensure that they are roughly balanced, as having a very small cluster could bias some of our metrics. We thus retained only maps in the top quartile of balance entropy (a measure of how evenly nodes are distributed across the three clusters). Third, picking the ‘top three’ maps could have yielded models with very similar perspectives, thus missing an occasion to examine the breadth of mental models that can exist for a given application domain. We thus maximized coverage by embedding the node labels of each map (using tf-idf) and we computed the cosine distances between maps. As a result, we have the distance for every pair of candidate maps, such as *d*(Babs, Clarice) = 0.719463. In the last step, we consider every possible group of three maps and we compute the sum of their distances. The triplet with the largest total score is selected because it contains three maps that are, collectively, as far apart from one another as possible.

We used the confidence interval (CI) method [77, pp. 182–193] to assess whether five replications were sufficient for these open-weight experiments on the obesity map. For each experimental condition and each metric, we used the mean and variability observed across the five completed runs to estimate how many replications would be needed to achieve a target confidence interval of either 90% or 95%. The results, reported in supplementary Table S3, show that only 65 of 12,000 combinations of metrics and experimental conditions (0.5%) would require more than five replications for a 90% confidence interval. Since a single additional run produces all metrics for a condition, these 65 cases boil down to 21 unique experimental setups. Fully powering them would require approximately 5,699 additional runs, because the required sample size increases sharply when the mean is small relative to the observed variance. This skew is informative because it reveals which cases have very high variability. All such cases come from Algorithms 1 and 2. While it may seem counter-intuitive (given the high variability of Algorithm 3 as shown in Table 9), it clarifies that the large variability of Algorithm 3 is structural rather than a consequence of insufficient replication.

We then applied Algorithm 3 to the three selected suicide-prevention maps. We kept both open-weight LLMs (Qwen and Ministral) and varied the causal map, the directed source–target subgraph pair, the presence or absence of an example, the number of generated words per source label (3 vs. 5), and the maximum depth of the expansion tree (1 vs. 2). In this follow-up experiment, we fixed factors that contributed to less than 5% of the variance: counter-examples (set to absent), greedy vs beam search (fixed to beam search with a value of 6 as it yields the best recall). This setting also avoids contrastive decoding, which performed poorly for Qwen on Algorithm 3. The final design therefore combined three suicide-prevention maps, two LLMs, three directed source–target subgraph pairs per map, two levels of the Example factor, two values for the number of generated words, two depth values, and five replications, yielding 3×2×3×2×2×2×5=720 runs.

The experiments on the three suicide prevention maps confirm that Algorithm 3 remains difficult outside the obesity case. Across the 72 experimental combinations, mean recall is low for both Qwen (15.3%) and Ministral (13.9%), and only 17 rows yield non-zero recall for at least one LLM. Performance is concentrated in specific maps and source-target directions rather than spread evenly across the design. One map yielded zero recall for both LLMs and was thus excluded from the variance decomposition, a second map yielded non-zero recall only for Qwen (so values are listed as N/A in Table 10 for Ministral), and the third yielded non-zero recall for both Qwen and Ministral. Prompt factors have smaller effects: examples generally reduce recall, using five generated words is modestly better than three, and greater depth only slightly helps Qwen. These results extend the earlier finding that Algorithm 3 is highly sensitive to the structure of the input maps and *operates in an underconstrained regime where success depends strongly on the source and target subgraphs.*

**Table 10:** Variance decomposition for Algorithm 3 on the suicide-prevention maps, excluding the map with no recall. Values are percentages. The Error column indicates whether the residual error term was retained in the decomposition. Rows are included when at least one LLM has a value of at least 10%. For Map 1, Ministral had zero recall.

Error	Map	Feature	Qwen Rec.	Ministral Rec.
Yes	1	Error	20.63	N/A
		Example	14.29	
		Example × Number of Words	14.29	
		Number of Words	14.29	
	2	Error	25.00	5.71
		Example	12.50	2.86
		Example × Number of Words	12.50	2.86
		Source–target pair	25.00	74.29
		Source–target pair × Example	25.00	5.71
No	1	Example	18.00	N/A
		Example × Number of Words	18.00	
		Number of Words	18.00	
		Source–target pair	12.00	
		Source–target pair × Example	12.00	
		Source–target pair × Number of Words	12.00	
	2	Example	16.67	3.03
		Example × Number of Words	16.67	3.03
		Source–target pair	33.33	78.79
		Source–target pair × Example	33.33	6.06

### Comparison with non-LLM baselines

We compared the LLM outputs with the two non-LLM baselines defined in the Materials and Methods section: WordNet-*k* and Random-*k*. We emphasize that the baselines do not perform the same modeling *process* as the LLM algorithms. Baselines do not generate intermediate concepts, perform chain-of-verification, or expand a conceptual model through prompting. Rather, the baselines provide reference points for interpreting the scores: WordNet-*k* asks how much performance can be obtained from lexical similarity between existing node labels, whereas Random-*k* asks how much can be obtained by chance when the same number of evaluated links is allowed. For fairness, both baselines are matched to the number *k* of evaluated cross-map links counted from that LLM output.

Table 11 reports the comparison using the primary metric for each algorithm. Algorithms 1 and 2 are summarized using accuracy, since these algorithms are evaluated as direct link-selection methods over a defined candidate space. Algorithm 3 is summarized using recall, because it is an exploratory tree-expansion method whose purpose is to recover possible bridges rather than to optimize precision or accuracy. This metric-specific presentation avoids comparing algorithms to baselines on measures that do not reflect their intended role.

**Table 11:** Comparison between LLMs and matched-*k* baselines on the primary metric for each algorithm. The “mean” columns average over all LLMs in the corresponding case. The “best LLM” columns show the strongest LLM and the baselines matched to that same LLM output. WordNet-*k* and Random-*k* use the same number of evaluated links as the corresponding LLM output. Suicide maps use the same numbering as in Table 10.

Alg.	Case	Metric	LLM mean	Baseline mean	Best LLM	Best LLM score	Word Net-*k*	Ran dom-*k*
1	Obesity	Accuracy	.9106	.9931	Gemini 2.5 Pro	.9249	1.00	1.00
2	Obesity	Accuracy	.8634	.9375	Gemini 2.5 Pro	.9254	1.00	1.00
3	Obesity	Recall	.0398	.0549	GPT-4o	.0866	.0875	.0875
3	Suicide #1	Recall	.0625	.0625	Qwen	.1250	.1250	.1250
3	Suicide #2	Recall	.3750	.3750	Ministral	.4167	.4167	.4167

The results show that the matched-*k* baselines can obtain high scores, but the interpretation is not that these baselines reproduce the LLM algorithms. Rather, a key reason is that the corrected value of *k* can be large (e.g., from 472 to 618 on average for Alg. 1 depending on the LLM): the LLM output may induce many evaluated links, particularly because they operate by creating intermediate nodes and filter later on. Once the baselines are allowed to return the same number of evaluated links, they may cover a large share of the admissible links between the two input maps. In such cases, *high baseline performance reflects the size of the evaluated link set* and the structure of the candidate space as much as the quality of the baseline method itself. This explains why the baselines can appear highly competitive in Table 11. For Algorithms 1 and 2, the high baseline accuracy indicates that, under the matched-*k* evaluation, simple direct selection can cover much of the relevant candidate space. For Algorithm 3, the obesity case has low recall for both LLMs and baselines, whereas the suicide-prevention maps show that LLM recall is closely matched by the baselines when the same number of evaluated links is allowed. The added value of the emerging practice of using LLMs for conceptual modeling should not be reduced to aggregate scores. Rather, the value proposition of LLMs should be contextualized with respect to their ability of generating intermediate structures that go beyond direct lexical or random coverage of the candidate space.

## Discussion

The emerging literature on using LLMs for conceptual modeling either uses a default temperature or sets it to a minimal value of zero with the intent of supporting replicability. Our empirical study questions this approach, which was established with prior LLM architectures but may lack validity given the variability inherent to models such as GPT-5, Gemini 2.5 Pro, DeepSeek v3.1, Qwen 3.5, or Ministral 3. Across our three algorithms to combine conceptual models and eight LLMs, results varied even when temperature was minimized and the prompts were fixed, indicating that elements beyond (or unknown to) a modeler’s control are driving the results. Even when a modeler exerts more control than usual by choosing the decoding strategy (which is possible with open-weight LLMs), our experiments demonstrated that variability can be present. Our $2^{k}r$ factorial analyses showed that some model combination approaches may amplify the inherent variability of LLMs, leading to situations in which randomness often dominates results by accounting for up to 96% of the variance in outcomes. We demonstrated that ignoring differences across repeated executions of a prompt by performing a simpler $2^{k}$ analysis would provide misleading results, wrongly attributing fluctuations in outcomes to controllable aspects such as prompt design (Tables 4–6) or decoding methods (Table 9). As a result, modelers may vastly overestimate the impact of controllable aspects.

Given these results, the modeling community ought to conduct experiments many times so that reported outcomes represent the *distribution* of possible outputs rather than risking being a result of chance. At present, across tasks in conceptual modeling, it appears that studies involving LLMs often conduct a single experiment or just a handful, and at most a few dozen experiments (Table 1). Our findings thus call for a significant change in practice: we need to perform replications and report not only mean results but also their dispersion. Our confidence interval analysis shows that this change does not necessarily call for thousands of repeats that could exceed available resources: most experimental conditions require only a handful of repeats, but some algorithms and settings have high variance. Our call for more, yet selected runs, is now feasible because the cost of running proprietary LLMs has decreased substantially, while a growing infrastructure supports the deployment of local LLMs. For instance, GPT-4 (2023 release) had a cost of 30$ per million input tokens, which became 5$ with GPT-4o (2024 release), 1.25$ with GPT-5, 0.55$ with DeepSeek-R1 (2025 release), and 0.20$ for GPT 5.4 Nano (2026 release). The growing affordability of LLMs thus opens the way to more thorough and statistically grounded evaluations of their use in conceptual modeling [21].

Our study has two main limitations. First, we considered five LLM families spanning both API-based models and locally executed open-weight models. The API models covered three LLM families with two versions each, covering contrasts between lightweight, latency-optimized models (Gemini 2.0 Flash) and reasoning-oriented ones (Gemini 2.5 Pro), as well as updates in widely used models (GPT-4 to GPT-5). The open-weight experiments added Qwen3.5-9B, a compact member of Alibaba’s Qwen family positioned for cost-efficient workloads, and Ministral 3 8B Instruct 2512, an instruction-tuned model from Mistral that is designed for efficient local deployment. While this is one of the most comprehensive use of LLMs in conceptual modeling and it suffices to reveal the presence of randomness and the mediating effect of the LLM, it cannot capture the full diversity of existing LLMs, including emerging architectures or specialized domain models. In our related work on transforming text into causal maps, we found that LLMs made different mistakes: Grok 3 struggled with ambiguous concept names, Claude 3.7 produced inconsistent terminology within a model (including a concept and its antonyms in the same causal map), and Llama 4 occasionally connected unrelated concepts [78]. Establishing the empirical behavior of a broader range of LLMs with known architectures would help attribute how architectural choices affect performance and, in turn, provide modelers with expectations about the behavior of newer LLMs. Second, our evaluation focused on the conceptual modeling task of model combination, so our findings may not generalize to other tasks such as model creation (e.g., generating a model from text) or model verification (e.g., comparing a model against a corpus). However, our open-source repository supports such extensions, since the analytical portion of our work (configuring and interpreting factorial designs of experiments across LLMs) can be directly reused.

Despite these limitations, the findings have broader implications for reproducibility in LLM-driven modeling tasks. We found that differences across model families and even across versions of the same family substantially alter which prompt elements appear most important. For example, features such as ‘providing examples’ or ‘representing data as array or list structures’ dominated some analyses with Gemini 2.0 Flash but lost influence in Gemini 2.5 Pro, while the same prompt combination produced different results between GPT-4o and GPT-5. This reinforces recent findings that model behavior can change across versions even under identical conditions [79], suggesting that model evolution alone can alter experimental conclusions. Consequently, results obtained with a given LLM version may not generalize to newer releases, and reproducibility across studies requires not only fixing temperature but also reporting the exact model version.

Our findings are consistent with reports that the effect of temperature varies by task and LLM [80,81]. Even when temperature is minimized, current models still exhibit stochasticity that interacts with prompt design. This observation aligns with prior evidence that LLM outputs can vary unpredictably across seemingly equivalent runs [7,8]. The persistence of randomness even at low temperatures indicates that architectural innovations, such as mixture-of-experts routing (in Gemini 2.5 Pro) or advanced sampling strategies (in DeepSeek v3.1), may contribute to intrinsic variability beyond what earlier transformer-only models exhibited.

Finally, while our study focused on combining conceptual models, the implications extend to other modeling activities such as text-to-model generation or model-to-text explanation [82,83]. Researchers and practitioners could build on our openly available experimental scripts to examine how randomness and model evolution affect other phases of the modeling life cycle. Future extensions may also integrate these analyses into modeling platforms based on Retrieval-Augmented Generation (RAG) [84] or frameworks like PRODIGY [85], equipping modelers with diagnostic tools that estimate expected variability and recommend the appropriate number of repeats for robust evaluation.

## Conclusion

Despite the dominant practice of evaluating a prompt for conceptual modeling only a few times, or setting the temperature to 0 to ‘support replicability’, our study finds that randomness can play a large role when combining conceptual models across a variety of techniques. In addition, even when pursuing the same goal of combining conceptual models, different algorithms react differently across LLMs, such that a prompting technique that was essential in one case (e.g., providing examples, giving inputs as lists of tags) may not be important in another setting. Our open-weight experiments further show that decoding strategy is not a neutral implementation detail: it can substantially affect performance, yet no single decoding strategy is uniformly best across algorithms and LLMs. These findings suggest that (i) replications should be used to identify where variability is concentrated, since most conditions may be adequately sampled while a small number of high-variance conditions may require many additional runs, and (ii) the design of a prompt would have to be re-optimized when going from one LLM version to the next. These suggestions are feasible in practice as the cost of LLMs continues to drop and our reusable workflow can be used to support local deployment. To best guide practitioners going forward, complementary investigations could relate architectural features of an LLM and decoding mechanisms (when known) to expectations for conceptual modeling tasks, which could save practitioners from repeatedly optimizing their solutions as the LLM ecosystem regularly changes.

## Supporting information

S1 Appendix Step-by-Step Algorithm Examples. For each of the three algorithms, we provide a succinct step-by-step description and illustration.
(PDF)

S1 Fig Visual summary of the first set of experiments. These experiments evaluate three algorithms to combine conceptual models across a factorial prompt design with replication, using six LLMs from the DeepSeek, Gemini, and GPT families. The figure summarizes the research questions, the experimental design to generate the data, the analyses applied to the results, and the interpretation targeted by each analysis. *The figure was generated by AI and edited manually.*
(PNG)

S2 Fig Visual summary of the second set of experiments. These experiments use Qwen and Ministral to examine variability under controlled decoding. The open-weight models allow decoding strategy to be treated as an explicit experimental factor. *The figure was generated by AI and edited manually.*
(PNG)

S3 Fig Visual summary of the process to select varied causal maps on suicide prevention. To demonstrate the multiplicity of viewpoints in conceptual modeling and cover several domains, our study uses maps from obesity and suicide prevention. The suicide prevention maps are selected through a systematic process in four steps. *The figure was generated by AI and edited manually.*
(PNG)

S1 Table Complete results for closed-weight models. Factorial analyses showing factors that contribute to at least 10% for at least one outcome (accuracy, precision, and recall) across three closed-weight models (DeepSeek, Gemini, GPT) and different versions, for all three algorithms, with and without accounting for the error term. This combines the data from Tables 4, 5, and 6 in a single table.
(CSV)

S2 Table Results from the second set of experiments (open weight LLMs) on obesity and suicide prevention. Variance is decomposed as a function of prompt elements and decoding methods, with/without considering the error term, for one causal map regarding obesity and three causal maps on suicide prevention.
(XLSX)

S3 Table Number of experiments needed for 90% and 95% Confidence Intervals. Across 12,000 condition–metric cells (i.e., combinations of experimental conditions and evaluation metrics), 65 (0.5%) would require more than five replications to achieve a 90% confidence interval. Because each replication yields all metrics simultaneously, this corresponds to 21 unique experimental conditions that are underpowered. Fully powering these conditions would require approximately 5,699 additional runs. Algorithms 1 and 2 are affected, whereas Algorithm 3 is not.
(CSV)

S4 Guide A brief guide to methods and tools for the Design of Experiments (DoE). Our paper uses DoE to identify sources of variation along with their interactions. To support the research community in reusing this approach with their own LLMs, a brief guide explains the main steps of DoE and points to tools that can readily support these tasks. More detailed guides can be found in [48,49].
(PDF)

## References

[1] Reitemeyer B, Fill HG. Applying large language models in knowledge graph-based enterprise modeling: challenges and opportunities. 2025. doi: 10.48550/arXiv.250103566

[2] Gutschmidt A, Nast B. Assessing Model Quality Using Large Language Models. Lecture Notes in Business Information Processing. Springer Nature Switzerland. 2024. p. 105–22. doi: 10.1007/978-3-031-77908-4_7

[3] Teubner T, Flath CM, Weinhardt C, van der Aalst W, Hinz O. Welcome to the era of chatgpt *et al*. the prospects of large language models. Business & Information Systems Engineering. 2023;65(2):95–101.

[4] Zhou L, Schellaert W, Martínez-Plumed F, Moros-Daval Y, Ferri C, Hernández-Orallo J. Larger and more instructable language models become less reliable. Nature. 2024;634(8032):61–8. doi: 10.1038/s41586-024-07930-y 39322679 PMC11446866

[5] Ye Q, Axmed M, Pryzant R, Khani F. Prompt engineering a prompt engineer. arXiv preprint. 2023. doi: arXiv:231105661

[6] Memmert L, Cvetkovic I, Bittner E. The more is not the merrier: Effects of prompt engineering on the quality of ideas generated by GPT-3. In: Proc. 57th Hawaii Int. Conf. on System Sciences, 2024. 7520–9.

[7] Cámara J, Troya J, Burgueño L, Vallecillo A. On the assessment of generative AI in modeling tasks: an experience report with ChatGPT and UML. Softw Syst Model. 2023;22(3):781–93. doi: 10.1007/s10270-023-01105-5

[8] Muff F, Fill HG. Limitations of ChatGPT in Conceptual Modeling: Insights from Experiments in Metamodeling. In: Modellierung 2024 Satellite Events, 2024. 10–18420.

[9] Peeperkorn M, Kouwenhoven T, Brown D, Jordanous A. Is temperature the creativity parameter of large language models?. 2024. https://arxiv.org/abs/240500492

[10] Hertling S, Paulheim H. OLaLa: Ontology Matching with Large Language Models. In: Proceedings of the 12th Knowledge Capture Conference 2023, 2023. 131–9. 10.1145/3587259.3627571

[11] Renze M, Guven E. The effect of sampling temperature on problem solving in large language models. 2024. doi: arXiv:240205201

[12] Hu M, Eidex Z, Wang S, Safari M, Li Q, Yang X. Benchmarking GPT-5 for Zero-Shot Multimodal Medical Reasoning in Radiology and Radiation Oncology. arXiv preprint. 2025. doi: arXiv:250813192

[13] Zhong S, Japkowicz N, Giabbanelli P. In: 2025. 512–21.

[14] Puigcerver J, Riquelme C, Mustafa B, Houlsby N. From sparse to soft mixtures of experts. In: 2023. https://arxiv.org/abs/230800951

[15] Saeedizade MJ, Blomqvist E. Navigating Ontology Development with Large Language Models. In: European Semantic Web Conf. Springer; 2024. p. 143–61.

[16] Jalali MS, Akhavan A. Integrating AI language models in qualitative research: Replicating interview data analysis with ChatGPT. System Dynamics Review. 2024;40(3):e1772.

[17] Snijder LL, Smit QTS, De Boer MHT. Advancing Ontology Alignment in the Labor Market: Combining Large Language Models with Domain Knowledge. AAAI-SS. 2024;3(1):253–62. doi: 10.1609/aaaiss.v3i1.31208

[18] Amini R, Norouzi SS, Hitzler P, Amini R. Towards complex ontology alignment using large language models. 2024. doi: arXiv:240410329

[19] Giglou HB, D’Souza J, Auer S. LLMs4OM: Matching Ontologies with Large Language Models. In: 2024. https://doi.org/arXiv:240410317

[20] Zhao Y, Vetter N, Aryan K. Using large language models for OntoClean-based ontology refinement. In: 2024. https://doi.org/arXiv:240315864

[21] He Y, Chen J, Dong H, Horrocks I. Exploring large language models for ontology alignment. 2023. https://arxiv.org/abs/230907172

[22] De Deyne S, et al. Can GPT-4 recover latent semantic relational information from word associations? A detailed analysis of agreement with human-annotated semantic ontologies. In: Proc. Wksh. Cognitive Aspects of the Lexicon, 2024. 68–78.

[23] Klievtsova N, Mangler J, Kampik T, Benzin JV, Rinderle-Ma S. How can generative AI empower domain experts in creating process models?. Wirtschaftsinformatik. 2024.

[24] Nivon Q, Salaün G. Automated Generation of BPMN Processes from Textual Requirements. Service-Oriented Computing. Singapore: Springer Nature Singapore; 2025. p. 185–201.

[25] Recker J, Green PF. How do Individuals Interpret Multiple Conceptual Models? A Theory of Combined Ontological Completeness and Overlap. JAIS. 2019;:1210–41. doi: 10.17705/1jais.00565

[26] Kim J, Hahn J, Hahn H. How Do We Understand a System with (So) Many Diagrams? Cognitive Integration Processes in Diagrammatic Reasoning. Information Systems Research. 2000;11(3):284–303. doi: 10.1287/isre.11.3.284.12206

[27] Voinov A, Jenni K, Gray S, Kolagani N, Glynn PD, Bommel P, et al. Tools and methods in participatory modeling: Selecting the right tool for the job. Environmental Modelling & Software. 2018;109:232–55. doi: 10.1016/j.envsoft.2018.08.028

[28] Schuerkamp R, Giabbanelli PJ, Grandi U, Doutre S. How to combine models? Principles and mechanisms to aggregate fuzzy cognitive maps. In: 2023 Winter Simulation Conference (WSC), 2023. 2518–29. 10.1109/wsc60868.2023.10408326

[29] Freixa J, Fernández-Silva S. Terminological variation and the unsaturability of concepts. Multiple perspectives on terminological variation. 2017;18:155.

[30] Freund AJ, Giabbanelli PJ. Automatically Combining Conceptual Models Using Semantic and Structural Information. In: 2021 Annual Modeling and Simulation Conference (ANNSIM), 2021. 1–12. 10.23919/annsim52504.2021.9552157

[31] Jean-Mary YR, Shironoshita EP, Kabuka MR. Ontology Matching with Semantic Verification. Web Semant. 2009;7(3):235–51. doi: 10.1016/j.websem.2009.04.001 20186256 PMC2825706

[32] Giabbanelli PJ, Tawfik AA. Overcoming the PBL Assessment Challenge: Design and Development of the Incremental Thesaurus for Assessing Causal Maps (ITACM). Tech Know Learn. 2017;24(2):161–8. doi: 10.1007/s10758-017-9338-8

[33] Giabelli A, Malandri L, Mercorio F, Mezzanzanica M. WETA: Automatic taxonomy alignment via word embeddings. Computers in Industry. 2022;138:103626. doi: 10.1016/j.compind.2022.103626

[34] Tounsi Dhouib M, Faron C, Tettamanzi AGB. Measuring Clusters of Labels in an Embedding Space to Refine Relations in Ontology Alignment. J Data Semant. 2021;10(3–4):399–408. doi: 10.1007/s13740-021-00137-8

[35] Voinov A, Shugart HH. ‘Integronsters’, integral and integrated modeling. Environmental Modelling & Software. 2013;39:149–58. doi: 10.1016/j.envsoft.2012.05.014

[36] Wang S, Petridis S, Kwon T, Ma X, Chilton LB. PopBlends: Strategies for Conceptual Blending with Large Language Models. In: Proceedings of the 2023 CHI Conference on Human Factors in Computing Systems, 2023. 1–19. 10.1145/3544548.3580948

[37] Osman I, Ben Yahia S, Diallo G. Ontology Integration: Approaches and Challenging Issues. Information Fusion. 2021;71:38–63. doi: 10.1016/j.inffus.2021.01.007

[38] White J, Fu Q, Hays S. A prompt pattern catalog to enhance prompt engineering with chatgpt. 2023. doi: arXiv:230211382

[39] Sahoo P, Singh AK, Saha S. A systematic survey of prompt engineering in large language models: techniques and applications. 2024. https://arxiv.org/abs/240207927

[40] Liu X, Wang J, Sun J. Prompting frameworks for large language models: a survey. 2023. https://arxiv.org/abs/2311.12785

[41] Macilenti G, Stellato A, Fiorelli M. Prompting is not all you need Evaluating GPT-4 performance on a real-world ontology alignment use case. Procedia Computer Science. 2024;246:1289–98. doi: 10.1016/j.procs.2024.09.557

[42] Hasan MA. Zero-and few-shot prompting with llms: A comparative study with fine-tuned models for bangla sentiment analysis. 2023. doi: arXiv:230810783

[43] Teehan R, Lake B, Ren M. CoLLEGe: Concept Embedding Generation for Large Language Models. In: First Conference on Language Modeling; 2024.

[44] Chen L, Xiao S, Chen Y, Sun L, Childs PRN, Han J. An artificial intelligence approach for interpreting creative combinational designs. Journal of Engineering Design. 2024;36(5–6):920–47. doi: 10.1080/09544828.2024.2377068

[45] Jang J, Ye S, Seo M. In: Transfer learning for natural language processing workshop, 2023. 52–62.

[46] Feng Z, Zhou H, Zhu Z, Qian J, Mao K. Unveiling and manipulating prompt influence in large language models. 2024. https://arxiv.org/abs/2405.11891

[47] Fatemi B, Halcrow J, Perozzi B. Talk like a graph: Encoding graphs for large language models. 2023. doi: arXiv:231004560\

[48] Sanchez SM, Sanchez PJ, Wan H. Work Smarter, Not Harder: A Tutorial on Designing and Conducting Simulation Experiments. In: 2020 Winter Simulation Conference (WSC), 2020. 1128–42. 10.1109/wsc48552.2020.9384057

[49] Sanchez SM. Simulation experiments: Better data, not just big data. In: 2015 Winter Simulation Conference (WSC), 2015. 800–11. 10.1109/wsc.2015.7408217

[50] Fisher RA. The design of experiments. Macmillan. 1935.

[51] Giabbanelli PJ. A guide to large language models in modeling and simulation: From core techniques to critical challenges. In: 2026. https://doi.org/arXiv:260205883

[52] Astekin M, Hort M, Moonen L. An Exploratory Study on How Non-Determinism in Large Language Models Affects Log Parsing. In: Proceedings of the ACM/IEEE 2nd International Workshop on Interpretability, Robustness, and Benchmarking in Neural Software Engineering, 2024. 13–8. 10.1145/3643661.3643952

[53] Ouyang S, Zhang JM, Harman M, Wang M. An Empirical Study of the Non-Determinism of ChatGPT in Code Generation. ACM Trans Softw Eng Methodol. 2025;34(2):1–28. doi: 10.1145/3697010

[54] Bender EM, Costello E, Lee K, Farrow R, Ferreira G. Unsafe AI for Education: A Conversation on Stochastic Parrots and Other Learning Metaphors ⚠. Journal of Interactive Media in Education. 2025;2025(1). doi: 10.5334/jime.1079

[55] Madabushi HT, Torgbi M, Bonial C. Neither stochastic parroting nor agi: Llms solve tasks through context-directed extrapolation from training data priors. arXiv preprint. 2025. doi: arXiv:250523323 https://arxiv.org/abs/250523323

[56] Joshi T, Aggarwal S, Saha A, Pandey A, Dhoot S, Rai V. Stochastic CHAOS: Why deterministic inference kills, and distributional variability is the heartbeat of artificial cognition. In: 2026. https://arxiv.org/abs/260107239

[57] Zhang Z, Ding X, Yuan J, Liu R, Mao H, Xing J. Deterministic inference across tensor parallel sizes that eliminates training-inference mismatch. In: 2025. https://arxiv.org/abs/251117826

[58] Ulakci E, Göbel JP, Huff M. Towards a psychology of machines: large language models predict human memory. PeerJ Computer Science. 2025;11:e3324. doi: 10.7717/peerj-cs.3324

[59] Huff M, Ulakci E. Judgments of learning distinguish humans from large language models in predicting memory. Sci Rep. 2025;15(1):35030. doi: 10.1038/s41598-025-22290-x 41057661 PMC12504528

[60] Kasai J, Sakaguchi K, Le Bras R, Radev D, Choi Y, Smith NA. A Call for Clarity in Beam Search: How It Works and When It Stops. In: Proceedings of the Language Resources and Evaluation Conference, 2024. 77–90. 10.63317/2c235hpjhnk8

[61] Li XL, Holtzman A, Fried D, Liang P, Eisner J, Hashimoto TB. Contrastive decoding: Open-ended text generation as optimization. In: Proceedings of the 61st Annual Meeting of the Association for Computational Linguistics (Volume 1: Long Papers), 2023. 12286–312.

[62] Frisoni G, Ragazzi L, Cohen D, Moro G, Carbonaro A, Sartori C. Abstractive summarization through the prism of decoding strategies. Neural Netw. 2026;196:108249. doi: 10.1016/j.neunet.2025.108249 41391353

[63] Presacan O, Nik A, Thambawita V, Ionescu B, Riegler M. A Comparative Study of Decoding Strategies in Medical Text Generation. In: Int. Conf. on Multimedia Modeling. Springer; 2026. p. 1–15.

[64] Nik A, Riegler MA, Halvorsen P. Impact of decoding strategies on GPU energy usage in large language model text generation. Sci Rep. 2025;16(1):2168. doi: 10.1038/s41598-025-31896-0 41381855 PMC12808663

[65] Giabbanelli PJ, Daumas C, Flandre NY, Pitkar A, Vazquez-Estrada J. Promoting empathy in decision-making by turning agent-based models into stories using large-language models. Journal of Simulation. 2025;:1–21. doi: 10.1080/17477778.2025.2536663

[66] Flandre NY, Giabbanelli PJ. Distilling the Complexity of Agent-Based Simulations into Textual Explanations via Large Language Models. BDCC. 2026;10(4):121. doi: 10.3390/bdcc10040121

[67] Dhuliawala S, Komeili M, Xu J, Raileanu R, Li X, Celikyilmaz A. Chain-of-verification reduces hallucination in large language models. 2023. https://arxiv.org/abs/2309.11495

[68] Schulhoff S, Ilie M, Balepur N, Kahadze K, Liu A, Si C. The Prompt Report: A Systematic Survey of Prompting Techniques. arXiv preprint. 2024. doi: 10.48550/arXiv.2406.06608

[69] Huang Z, Quan K, Chan J, MacNeil S. CausalMapper: Challenging designers to think in systems with Causal Maps and Large Language Model. In: Proceedings of the 15th Conference on Creativity and Cognition, 2023. 325–9.

[70] Castelo N, Katona Z, Li P, Sarvary M. How AI outperforms humans at creative idea generation. In: 2024. https://ssrn.com/abstract=4751779

[71] Giabbanelli PJ, MacEwan G. Leveraging Artificial Intelligence and Participatory Modeling to Support Paradigm Shifts in Public Health: An Application to Obesity and Evidence-Based Policymaking. Information. 2024;15(2):115. doi: 10.3390/info15020115

[72] OpenAI. Text-embedding-3-large model documentation. https://developers.openai.com/api/docs/models/text-embedding-3-large 2026.

[73] Zhang Y, Li M, Long D, Zhang X, Lin H, Yang B. Qwen3 embedding: Advancing text embedding and reranking through foundation models. arXiv preprint. 2025. doi: arXiv:250605176

[74] Dao J, Li Z, Su Y, Tang X, Zhou Q, Lan X, et al. Qwen3-BiLSTM: Lightweight Clinical NER with Embedding-Sequence Synergy. In: 2025 6th International Conference on Artificial Intelligence and Electromechanical Automation (AIEA), 2025. 01–4. 10.1109/aiea66061.2025.11160548

[75] Niu H, Bryan J, Li X, Li D. Incorporating LLM embeddings for variation across the human genome. In: 2025. https://doi.org/arXiv:250920702

[76] Giabbanelli PJ, Rice KL, Galgoczy MC, Nataraj N. Pathways to suicide or collections of vicious cycles? Understanding the complexity of suicide through causal mapping. Social Network Analysis and Mining. 2022;12(1):60. 10.1007/s13278-022-00886-9 PMC9285107 35845751

[77] Robinson S. Simulation: The Practice of Model Development and Use. 2nd ed. Basingstoke, UK: Palgrave Macmillan. 2014.

[78] Sakib SK, Liddle SW, Lynch CJ, Agrawal A, Giabbanelli PJ. Rethinking Learning: The Role of Unlearning in Generative AI-Based Conceptual Modeling. In: Proc. 44th Int. Conf. on Conceptual Modeling (ER 2025). Poitiers / Futuroscope, France: Springer; 2025.

[79] Davis J, Van Bulck L, Durieux BN, Lindvall C. The Temperature Feature of ChatGPT: Modifying Creativity for Clinical Research. JMIR Hum Factors. 2024;11:e53559. doi: 10.2196/53559 38457221 PMC10960206

[80] Zack T, Lehman E, Suzgun M, Rodriguez JA, Celi LA, Gichoya J, et al. Assessing the potential of GPT-4 to perpetuate racial and gender biases in health care: a model evaluation study. Lancet Digit Health. 2024;6(1):e12–22. doi: 10.1016/S2589-7500(23)00225-X 38123252

[81] Akamine A, Hayashi D, Tomizawa A, Nagasaki Y. Effects of temperature settings on information quality of ChatGPT-3.5 responses: A prospective, single-blind, observational cohort study. medRxiv. 2024;:2024–06.

[82] Fill HG, Fettke P, Köpke J. Conceptual modeling and large language models: impressions from first experiments with ChatGPT. Enterprise Modelling and Information Systems Architectures (EMISAJ). 2023;18:1–15.

[83] Giabbanelli PJ, Padilla JJ, Agrawal A. Broadening Access to Simulations for End-Users via Large Language Models: Challenges and Opportunities. In: 2024 Winter Simulation Conference (WSC), 2024. 2535–46. 10.1109/wsc63780.2024.10838754

[84] Minor M, Kaucher E. Retrieval augmented generation with LLMs for explaining business process models. In: Int. Conf. on Case-Based Reasoning. Springer; 2024. p. 175–90.

[85] Ziche C, Apruzzese G. LLM4PM: A case study on using Large Language Models for Process Modeling in Enterprise Organizations. In: Int. Conf. on Business Process Management. Springer; 2024. p. 472–83.

