# Supplementary material for: On the variability of generative artificial intelligence methods in conceptual modeling: an experimental evaluation on combining causal maps
Source: PLoS One. 2026 Oct 27;21(10):e0361281. doi: 10.1371/journal.pone.0361281 (PMC13712584; doi:10.1371/journal.pone.0361281)
Supplement: Supplementary file 1 [file pone.0361281.s001.pdf]

Step-by-Step Algorithm Examples

Method 1: Direct Combination with Chain-of-Verification

Example Input Maps

- **Map A (Psychology):** Stress → Burnout
- **Map B (Nutrition):** Healthy Eating → Obesity

Step-by-Step Process

1. **Initial Prompt:** Request edges linking Map A and Map B.
2. **LLM Proposal:** Suggests intermediate node ”Dietary Choices” with edges:
   - Stress → Dietary Choices
   - Dietary Choices → Obesity
3. **Verification Prompt:** Validate proposed edges.
4. **Verified Output:** Confirms valid edges after verification.

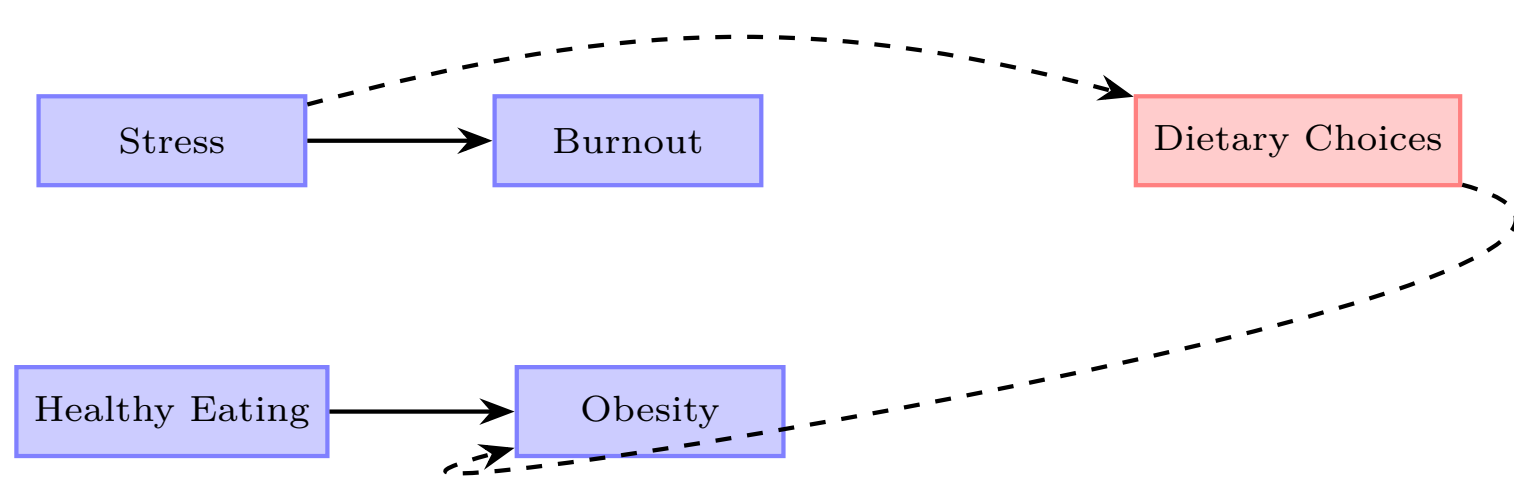

Figure 1: Method 1: Direct combination with intermediate node proposal (dashed lines). Verified edges become solid after validation.

Method 2: Growing Links via Word Embeddings

Example Input Concepts

- **Source Terms:** Stress, Burnout
- **Target Terms:** Healthy Eating, Obesity

Step-by-Step Process

1. **Expand Concepts:** Generate related terms for ”Stress”:
   - Anxiety, Cortisol, Sleep Quality
2. **Compute Embeddings:** Calculate cosine similarity between expanded terms and target map.
3. **Stabilization Check:** Iterate until similarity changes <1%.
4. **Final Links:** Match ”Cortisol” (source) → ”Obesity” (target).

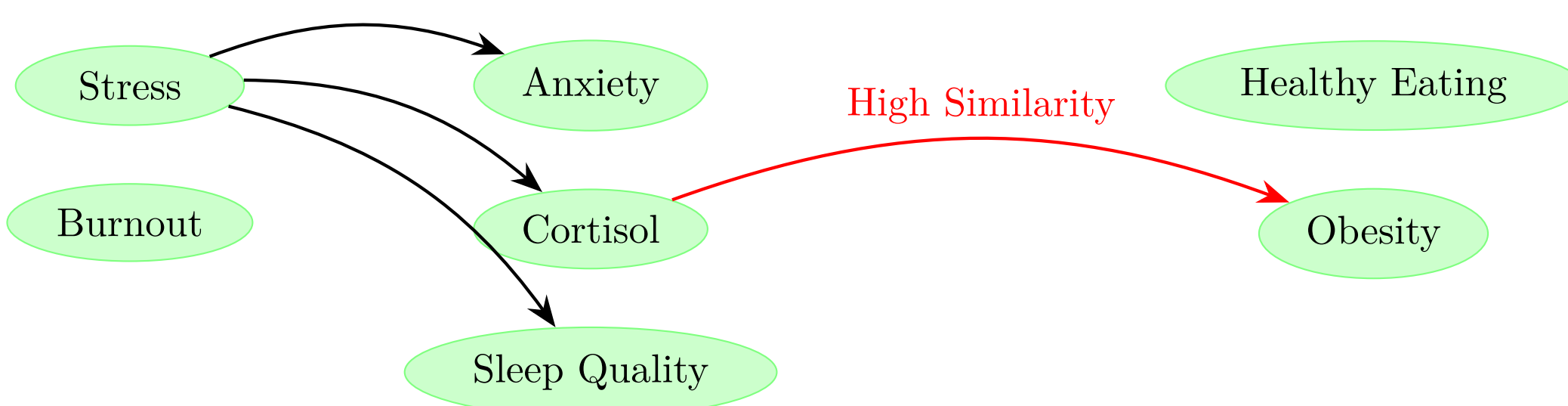

Figure 2: Method 2: Semantic expansion and embedding-based matching. Red arrow indicates validated link.

Method 3: Tree-Based Indirect Matching

Example Input

- **Source Node:** Stress (from Map A)
- **Target Map:** Healthy Eating → Obesity (Map B)

Step-by-Step Process

1. **Depth 1 Expansion:** Generate related terms for ”Stress”:
   - Anxiety, Fatigue
2. **No Direct Match:** Check against target map terms.
3. **Depth 2 Expansion:** Expand ”Anxiety” → ”Sleep Disorders”.
4. **Indirect Match:** Link ”Sleep Disorders” → ”Obesity”.

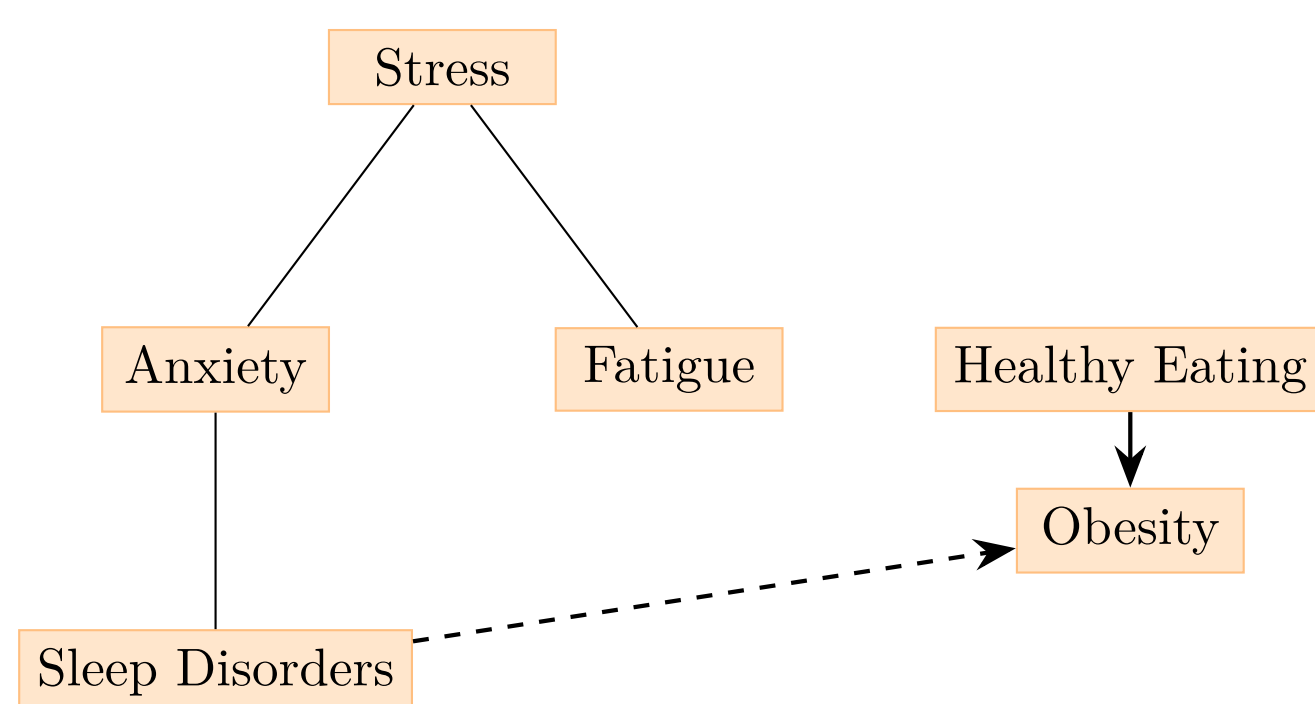

Figure 3: Method 3: Hierarchical expansion to bridge concepts across maps. Dashed line shows indirect match.
