# Supplementary material for: On the variability of generative artificial intelligence methods in conceptual modeling: an experimental evaluation on combining causal maps
Source: PLoS One. 2026 Oct 27;21(10):e0361281. doi: 10.1371/journal.pone.0361281 (PMC13712584; doi:10.1371/journal.pone.0361281)
Supplement: Supplementary file 2 [file pone.0361281.s008.pdf]

A Short Guide to Design of Experiments

For stochastic model and prompt studies

23 August 2026

Abstract

Design of experiments (DoE) is a practical way to plan runs so that several inputs can be studied together. This guide explains which design to choose, which tools can generate and analyse it, and how to report noisy results in a way that others can check.

1 What DoE is for

In a one-factor-at-a-time (OFAT) study, one input changes while the others stay fixed. This is easy to understand, but it can miss an interaction: the effect of one input may depend on the value of another. A factorial DoE varies inputs together in a planned pattern, so main effects and selected interactions can be estimated from the same study [1, 4].

For example, if a prompt has two optional elements, a two-level full factorial design runs all four combinations. With k two-level factors, a full design has 2^k settings. This is informative when k is small, but it can become expensive quickly. A fractional design can use fewer settings, at the cost of some effects being aliased (confounded) with others.

2 A small, repeatable workflow

1. **State the question.** Are you screening many possible inputs, estimating interactions, or looking for a good setting?
2. **Choose the response.** Define the outcome before running the experiment. Include its direction: higher, lower, or closer to a target.
3. **Name the factors and levels.** Record the exact values that will be used, along with constraints and settings that must not change.
4. **Choose and inspect the design.** Check that every planned effect is estimable and that the number of runs is affordable.
5. **Randomize, block, and repeat.** Randomize run order when possible. Use blocks for known sources of drift, such as model version, batch, map, or day. Repeat each condition enough to see stochastic variation.
6. **Fit, check, and confirm.** Fit the pre-specified model, inspect residuals and aliasing, report the result distribution, and confirm an important finding on fresh runs or held-out seeds.

Keep the design table and the raw row-level results. Do not keep only the average or the best output. Randomization, blocking, replication, and checking are standard parts of a defensible experiment [2, 3].

3 Choose a design

Question	Useful first choice	Main caution
Few factors; interactions matter	Full factorial	The number of settings grows as 2^k for k two-level factors.
Many candidate factors; first screen	Fractional factorial or Plackett–Burman	Aliasing can hide or rename effects. Inspect the alias structure and do not claim an interaction that the design cannot separate.
Numerical factors; curvature or an optimum matters	Response-surface design, such as a central composite or Box–Behnken design	Choose sensible ranges and include centre points when they help detect curvature.
Broad coverage for a simulation or parameter search	Latin hypercube or Sobol sequence	These are space-filling designs. They are useful for coverage, but are not automatically a factorial test of causal interactions.
Runs span known batches or time periods	Randomized block design	Randomize within each block and keep the block variable in the analysis.

A fractional design is a shortcut, not a free reduction in complexity. Its alias structure says which effects cannot be distinguished with the available runs. Response-surface designs are intended for modelling curvature and optimization, while randomized blocks protect comparisons from known nuisance variation [1, 3, 6].

4 Tools that do the arithmetic

The best choice is usually the tool that the team can run, inspect, and keep with the study. Start with the question and constraints; do not ask a package to choose a scientifically meaningful response or factor range for you.

Route	Good for	What it provides
JMP	Little code and a visual workflow	Full and fractional factorial designs, screening, response surfaces, blocking, and design evaluation [5].
R: `DoE.base` + `FrF2`	Open, scriptable factorial work	Full factorial and orthogonal-array utilities; two-level fractional designs, plots, and readable alias information [7, 8].
R: `rsm`	Response-surface analysis	First- and second-order models, surface plots, steepest ascent, and canonical analysis [9].
Python: `PyDOE`	Generating design tables in code	Factorial, screening, response-surface, blocking, space-filling, and optimal-design utilities [6].
Python: SciPy QMC	Space-filling numerical inputs	Latin hypercube, Sobol, Halton, and discrepancy tools. Sobol designs have preferred sample sizes, usually powers of two [10].
Python: statsmodels	Fitting and checking models	Formula-based linear models and ANOVA tables, including interaction terms [11].

Use a GUI when it reduces setup mistakes. Use scripts when the design must be regenerated, reviewed, or attached to a reproducible pipeline. In either case, save the generated design matrix, the package version, and the final analysis script.

5 A shortcut for stochastic runs

For a prompt or model experiment, the following small recipe is often enough to get started:

1. Make one design row for each combination of factor levels. Give each row a stable condition identifier.
2. Add a replicate identifier and an execution seed when the system supports seeds. If provider randomness is not fully controlled, record the model version, request settings, and run identifier instead.
3. Freeze the table before running. Randomize its execution order and add a block column for unavoidable changes such as model, dataset, or day.
4. Run every row repeatedly. Store one row per execution, including failures, latency, cost, and the response used for analysis.
5. Fit the main effects and only the interactions that the design can estimate. Check residuals, sparse cells, aliasing, and sensitivity to reasonable analysis choices.
6. Report each condition as a distribution: sample size, centre, spread, and useful quantiles or intervals. Treat a promising setting as a hypothesis until fresh runs support it.

This workflow makes stochastic noise visible instead of hiding it inside one summary score. It also keeps the design, execution, and analysis separate, so a reader can reproduce the study or replace one tool with another.

6 Minimum reporting checklist

Report:

- the question, response definition, factor names, levels, and constraints;
- the design type, number of planned settings, replication count, and any blocking or randomization rule;
- the model formula, interaction terms, alias or resolution information, and any exclusions;
- model, provider, prompt, dataset, software, and execution settings that could affect the response;
- distributions or uncertainty for each important condition, not only a ranking of averages; and
- the design table, raw results, analysis code, and package versions.

References

[1] National Institute of Standards and Technology, "Modeling," NIST/SEMATECH e-Handbook of Statistical Methods. online

[2] National Institute of Standards and Technology, "Choosing an Experimental Design," NIST/SEMATECH e-Handbook of Statistical Methods. online

[3] National Institute of Standards and Technology, "Blocking in Factorial Designs," NIST/SEMATECH e-Handbook of Statistical Methods. online

[4] JMP, "Design of Experiments Workflow." online

[5] JMP, "Design of Experiments Capabilities." online

[6] PyDOE, "PyDOE: An Experimental Design Package for Python." online

[7] CRAN, "DoE.base: Full Factorials, Orthogonal Arrays and Base Utilities for DoE Packages." online

[8] CRAN, "FrF2: Fractional Factorial Designs with 2-Level Factors." online

[9] CRAN, "rsm: Response-Surface Analysis." online

[10] SciPy, "Quasi-Monte Carlo submodule." online

[11] Statsmodels, "`statsmodels.stats.anova.anova_lm`." online
