# Supplementary material for: On the variability of generative artificial intelligence methods in conceptual modeling: an experimental evaluation on combining causal maps
Source: PLoS One. 2026 Oct 27;21(10):e0361281. doi: 10.1371/journal.pone.0361281 (PMC13712584; doi:10.1371/journal.pone.0361281)
Supplement: Supplementary file 3 [file pone.0361281.s011.pdf]

VMASC
OLD DOMINION UNIVERSITY

ODU
Office of Enterprise Research and Innovation

Tjeerd V. olde Scheper, Ph.D.
Academic Editor
PLOS One

October 7, 2025

Dear Dr. Tjeerd V. olde Scheper and reviewers,

We appreciate the detailed and actionable feedback provided by the reviewers. We have significantly revised our manuscript through extensive additional experiments thanks to this guidance. This letter responds to the points raised by the academic editor and reviewers, where our response is shown in blue below each original point. We look forward to hearing from you and we appreciate your time.

Journal Requirements:

When submitting your revision, we need you to address these additional requirements.

1. Please ensure that your manuscript meets PLOS ONE's style requirements, including those for file naming. The PLOS ONE style templates can be found at https://journals.plos.org/plosone/s/file?id=wjVg/PLOSOne_formatting_sample_main_body.pdf and https://journals.plos.org/plosone/s/file?id=ba62/PLOSOne_formatting_sample_title_authors_affiliations.pdf

>> Thank you for this reminder. We have used the LaTeX style files and followed the template. Naturally, we are available to make any change and work with the editing office if any discrepancy is observed during the editorial workflow.

2. Please note that PLOS One has specific guidelines on code sharing for submissions in which author-generated code underpins the findings in the manuscript. In these cases, we expect all author-generated code to be made available without restrictions upon publication of the work. Please review our guidelines at https://journals.plos.org/plosone/s/materials-and-software-sharing#loc-sharing-code and ensure that your code is shared in a way that follows best practice and facilitates reproducibility and reuse.

>> We fully support these practices, which we perform across journals. Our revised code is now hosted on the Open Science Framework (a PLOS-approved repository) with a permanent DOI. The license is clearly stated as MIT license. We have provided a workflow to support future users in re-running our experiments, and we have also provided input and output files accordingly. Our Data Availability Statement was amended to the following in the online submission form:

The full dataset is accessible at the Open Science Framework (OSF) under the permanent DOI **https://doi.org/10.17605/OSF.IO/KGDRU**, as referenced in the manuscript. This public repository requires no registration for access and includes the code used in this study under the MIT License, all input files, raw LLM outputs, and the complete analysis workflow and results.

3. Thank you for uploading your study's underlying data set. Unfortunately, the repository you have noted in your Data Availability statement does not qualify as an acceptable data repository according to PLOS's standards.
At this time, please upload the minimal data set necessary to replicate your study's findings to a stable, public repository (such as figshare or Dryad) and provide us with the relevant URLs, DOIs, or accession numbers that may be used to access these data. For a list of recommended repositories and additional information on PLOS standards for data deposition, please see https://journals.plos.org/plosone/s/recommended-repositories.

>> While we were using the PLOS-approved Open Science Framework (OSF) as repository, we did not create a permanent DOI for our initial submission. We have now remedied this shortcoming for the revision so that it adheres to PLOS's standards.

4. If the reviewer comments include a recommendation to cite specific previously published works, please review and evaluate these publications to determine whether they are relevant and should be cited. There is no requirement to cite these works unless the editor has indicated otherwise.

>> We read and evaluated the recommendations from reviewers. We ultimately found them reasonable and included them in our revised bibliography.

Additional Editor Comments:
Kindly address the comments from both reviewers, especially with respect to validation and include in the revision a focus on the requested improvements on evidential support of the claims made in your manuscript.

>> Thank you for emphasizing the importance of validation. We have addressed all comments (as detailed below). Our revision made the following main changes:

- The revised manuscript now includes a second experimental suite using open-weight models, Qwen and Mistral, to study decoding strategy as an explicit source of variability rather than treating generation as a fixed API behavior. The new experiments compare greedy decoding, beam search, and contrastive decoding with different parameter settings.
- We added a new background subsection on sources of variability in LLM outputs.
- The revised manuscript now compares LLM outputs with two non-LLM baselines.
- We added extensive statistical analyses (paired factor-level tests, confidence intervals, t-statistics, raw p-values, and Benjamini–Hochberg adjusted q-values) to assess whether prompt factors have consistent directional effects.
- We now estimate how many replications would be needed to achieve 90% and 95% confidence intervals.

- We generalized the finding by adding another domain (suicide prevention) using three causal maps selected through a systematic process to maximize variety.

Please ensure that your abstract and conclusion reflect the improvements you will make on the basis of these reviews.

>> As recommended, we have updated the abstract and the conclusion to reflect the (very) extensive changes that we made. Differences have been marked in track changes (red font).

Reviewer #1:

The manuscript examines the extent to which variability in contemporary large language models shapes outcomes in conceptual modeling, using the task of combining causal maps as an empirical testbed. The authors motivate their work from the rapidly expanding literature on LLM-assisted conceptual modeling and correctly identify a methodological blind spot: most prior studies either rely on default temperature settings or set temperature to zero while implicitly assuming quasi-deterministic behavior. Building on this observation, the paper derives its central hypotheses from work on prompt engineering, model architecture, and stochastic decoding, and operationalizes them through a carefully constructed full factorial Design of Experiments with replication. Three representative model-combination algorithms are implemented, spanning direct edge generation, creative expansion via embeddings, and multi-step tree-based reasoning, and are evaluated across three LLM families and two successive versions per family. The experimental design is rigorous, transparent, and well documented, and the discussion convincingly demonstrates that variability can dominate performance outcomes, even at minimal temperature settings, thereby challenging common assumptions about reproducibility in LLM-based modeling studies. By emphasizing distributional outcomes rather than single-run results, the manuscript makes a valuable methodological contribution to the conceptual modeling community.

>> Thank you for this thorough and very fair summary of our work!

A major point of criticism concerns the interpretation of variability. Throughout the manuscript, large error terms in the factorial analyses are primarily framed as stochastic noise that undermines experimental control. However, variability may itself be structured and informative rather than purely random.

>> This was an excellent point. We agree that variability should not automatically be interpreted as undifferentiated stochastic noise. Part of the residual variation may instead be structured and *informative*, revealing method-specific instability, differences in the types of outputs generated across repetitions, or sensitivity to the inference procedure itself. We have therefore revised the manuscript to make this point explicit and to investigate it directly. In the revised paper, the new analyses now argue that this residual variability can mean different things depending on the method. First, the variability is method-specific, not generic. Algorithms 1 and 2 are almost perfectly stable under repetition, whereas Algorithm 3 is not. That already reveals that the residual is related to the design of the method, not just to unavoidable random fluctuation in all LLM calls.

Second, our new investigations into the variability for Algorithm 3 shows that the repeated runs are exploring substantially different edge sets, not just adding or dropping one or two edges around a stable backbone. In other words, the variability has a recognizable pattern: the method behaves like an under-constrained branching search, not like a nearly deterministic extractor. This provides further evidence for the reviewer's suggestion about prompts as (insufficient) constraints. We also added additional test to rule out malformed outputs or trivial parsing failures, thus confirming that Algorithm 3 operates in a different regime than the other algorithms.

For example, Huff and Ulakci's study in Scientific Reports shows that LLMs systematically fail to replicate human metacognitive judgments of learning (JOLs) even when surface-level performance aligns with humans, revealing a structured divergence in how models interpret and predict memory outcomes. This suggests that variability across runs and models may reflect underlying representational ambiguity or limitations in how prompts constrain the model's internal hypothesis space, rather than mere stochastic noise. Integrating this perspective would help reframe the authors' key finding: instead of concluding that prompt elements "matter very little" when randomness dominates, one could argue that prompts fail to sufficiently constrain conceptual inference, leading to systematic differences in latent reasoning trajectories.

>> We agree that our study lacked a clear overview of how variability was produced and what it can mean, particularly in the context of conceptual modeling. We have added a new comprehensive subsection to our background, named "Potential Sources of Variability in LLM outputs". It contains the references kindly provided by the reviewer as well as many other references to clearly indicate that there are multiple source of variability, and that prompts play a role in constraining variability alongside decoding methods.

A related point of critique is that the discussion remains largely descriptive with respect to architectural differences. While variations across LLM families and versions are documented, the manuscript stops short of linking these differences to specific computational mechanisms. For instance, work by Ulakci, Göbel, and Huff in PeerJ Computer Science dissects how LLM outputs can align with human cognitive data on some dimensions (e.g., relatedness and memorability ratings) yet diverge in others, indicating that variability often has interpretable structure connected to context dependency rather than being irreducible noise . This implies that treating "the LLM" as a single factor may obscure meaningful sub-mechanisms such as decoding strategy differences, latent uncertainty, or multi-step amplification effects. Explicitly engaging with these ideas would strengthen the explanatory power of the manuscript's factorial analysis.

>> We agree that the previous version of the manuscript treated variability largely descriptively and did not sufficiently connect it to underlying computational mechanisms. To address this point, we have substantially revised the manuscript by introducing a new subsection ("Potential Sources of Variability in LLM Outputs"), in which we explicitly distinguish between multiple sources of variability: inference non-determinism, model-identity instability, and distributional bias. This clarification allows us to move beyond treating "the LLM" as a single factor and instead frame variability as arising from distinct mechanisms acting at different levels of the system. In particular, we now emphasize the role of the decoding process as a key mechanism linking model behavior

to observable variability. While prior experiments relied on proprietary LLMs with largely sampling-based decoding interfaces, we complement them with new experiments using open-weight models (Qwen and Mistral), which allow us to explicitly control decoding strategies. We systematically compare greedy decoding, beam search (with different beam widths), and contrastive decoding (with different penalties), thereby isolating how different decoding mechanisms affect both performance and the structure of outputs.

Another substantive issue is the limited connection to broader theory in conceptual modeling and cognition. Although the manuscript cites work on integronsters and model integration, the implications of the findings for how conceptual models function as cognitive or epistemic artifacts are not fully developed. The evaluation framework assumes a single ground-truth causal map and treats deviations as errors, yet conceptual modeling literature emphasizes that combining partial perspectives can legitimately yield emergent, and sometimes desirable, structures. A more explicit discussion of this tension would clarify whether variability reflects genuine model ambiguity, evaluation limitations, or systematic divergence due to underconstrained inference. Relatedly, reliance on a single substantive domain, obesity, limits claims about generality across domains with different semantic structures or contextual complexities.

>> In hindsight, we agree that only working on obesity was limiting. We have thus added the topic of suicide prevention. We also strongly believe that it is normal and even expected that causal maps (when used as mental models for individual perspectives) will have deviation, so there is no single ground-truth. Fortunately, our dataset for suicide prevention provides several maps, so we were able to study the effect of these differences. We selected three maps on suicide prevention through a systematic procedure designed to maximize diversity of perspectives: we first identified structurally balanced partitions (as the algorithm needs to link sub-groups that should not differ widely in sizes to avoid biasing estimates), then retained maps with high balance entropy, and finally selected triplets that maximize pairwise semantic distance using Earth Mover's Distance over TF-IDF embeddings of node labels. This process ensures that the selected maps represent substantively different yet plausible conceptualizations of the domain.
We have revised the subsection “Combining conceptual models” to explicitly acknowledge that conceptual modeling does not necessarily admit a single ground-truth representation, and that is why we usually solicit different perspectives before combining them. That is, the very existence of the combination task is motivated by the absence of a single ground truth, so we elicit different perspectives and then combine them in a controlled manner.

On more minor points, the introduction could be tightened to foreground the research questions earlier and reduce some repetition in the literature review. Some terminology alternates between "knowledge maps", "conceptual models", and "causal maps" without consistent definitions, which may confuse readers outside the immediate community.

>> The reviewer is correct that the introduction included some of the literature review and that it took a long time to get to the research questions. We thus streamlined the introduction significantly; the first three paragraphs were reduced by about half. We also clarified that causal maps are conceptual models, so that our study is motivated by (and contributes to) LLM for

conceptual modeling. We also edited our manuscript for concision by streamlining the first subsection of the background.

The presentation of results, especially the large factorial tables, would benefit from additional interpretive guidance or visual summaries to support readers less familiar with Design of Experiments.

>> We agree with this suggestion. Although we attempted to support readers through a section on Factorial Design of Experiments (DoE) that shows how results are computed, we now realize that visual summaries were missing. We added Figure 7 before going into the large factorial table, and we also added supplemental figures to illustrate the complete design of our experiments. Rather than add many other tables in the manuscript, we have provided secondary ones as supplementary documents.

From an open science perspective, the authors should be commended for sharing data and scripts via OSF, but the manuscript would be strengthened by an explicit discussion of preregistration and by a principled justification of the number of replications, for example via power or precision considerations rather than feasibility alone. Reporting confidence intervals or distributional plots alongside mean performance would further reinforce the paper’s core message about variability.

>> Thank you for this excellent idea. We have reported distributional plots (Figure 7) and performed an analysis using the Confidence Interval method to determine the number of replications, which is discussed in our results and reported in supplementary table S3. We acknowledge that the study was not preregistered and our understanding is that a restrospective preregistration would not be the right process. We hope that our extensive disclosure of scripts, inputs, outputs, and analyses on OSF will address some of these concerns.

Relevant literature that could strengthen the manuscript includes work on metacognitive differences between humans and LLMs, such as Huff & Ulakci’s study demonstrating structured divergence in predictive judgment tasks; analyses showing how different architectural and decoding strategies contribute to output behavior; and broader cognitive modeling perspectives on how emergent structures and underdetermination affect conceptual inference. Engaging more directly with these sources would help position variability not only as a methodological challenge but also as a theoretically informative phenomenon that can advance our understanding of both LLM behavior and conceptual modeling practices.

>> We have expanded our references accordingly. While some were deleted by following the reviewer’s suggestion to streamline the introduction, many other references were added, particularly in a new dedicated background subsection on decoding strategies. We have cited the two references provided on under-determination, and we look forward to learning more about cognitive modeling perspective as some aspects may be beyond our expertise.

I attempted to review the data and materials at the OSF repository linked in the manuscript (https://osf.io/kgdru/), but the project page did not load or reveal any accessible files at the time of review, suggesting that the repository may be private, restricted, or otherwise unavailable for public inspection (I checked it on different browsers). Because OSF projects can be set to public or private at the discretion of the authors, and because successful reuse of data depends on discoverability and access, this prevented me from independently evaluating the shared data and scripts in support of the manuscript's claims.

>> We apologize for this mistake. We indeed left the repository to be private (by default). We have now addressed this oversight by making it public and also creating a permanent DOI. The shared data and scripts should now be accessible. For the convenience of the reviewers and future users, we have also provided the scripts and a guide in a Github project that can quickly be cloned and deployed, while the large volumes of outputs are stored in Hugging Face's buckets (links are provided on OSF within the readme file).

Reviewer #2:

General Assessment

The paper presents a significant issue in the conceptual modeling with the use of LLM, proving that randomness may take over even in the lowest temperature conditions. A methodological strength is the use of the factorial design. Nevertheless, the paper needs to be revised on some of its critical areas before publication. My particular areas of concern are as follows:

>> We appreciate the practical recommendations formulated by the reviewer. They were helpful in expanded the scope and methodology of this paper.

Major Concerns

1. Limited Experimental Scope

The article uses only one data of obesity causal maps, which seriously restricts the generalizability arguments. It is not enough to test only health-related models to make the conclusion that the results can be generalized to conceptual modeling tasks. The semantic structures in different domains (business processes, software engineering, environmental systems) are radically different and can have different interactions with the capabilities of LLM. Add at least one more domain with other features to reinforce arguments that it is cross-domain.

>> This is a very valid point. We added a second domain of suicide prevention and used three causal maps (selected by a procedure that maximizes diversity). Suicide prevention touches on domains at very different scales (e.g., individuals, their communities, society) and on different themes (e.g., mental health, economic policies, healthcare system, social norms).

2. Insufficient Statistical Rigor

The article does not have basic statistical hypothesis testing. No ANOVA F-tests, p-values, and confidence intervals are provided and it is impossible to differentiate between meaningful effects and sampling noise. The decision to use 5 replications does not seem to be justified by power analysis. Add formal statistical testing with multiple comparison adjustments, provide confidence

intervals of all performance measures, and rationalize replication figures by demonstrating power or convergence.

>> We thank the reviewer for pointing out the need for more statistical analyses. We have completed a series of analyses accordingly.

Using the Confidence Interval (CI) method, we have added an assessment on how many runs are sufficient. For each experimental condition and each performance metric (e.g., accuracy, precision, recall, F1), we use the observed mean and variability across the initial runs to estimate how many replications would be required to achieve a target relative confidence interval (set to either 90% or 95%). This approach directly evaluates whether variability is small enough for reliable estimation. Our results show that 99.5% of cases are sufficiently powered for a 90% CI. The remaining 0.5% of cases (i.e., 65 combinations of conditions and metrics out of 12,000 cases) would require an additional 5,699 additional runs, which would be a double digit increase in the experimental budget. This situation happens because the required sample size increases sharply when the mean is small relative to the variance. *This skew is itself an important additional finding: it reveals that variability is highly concentrated in a small subset of conditions, rather than uniformly distributed across the design*. We have added this finding to the manuscript and provided the additional table with the 90% and 95% CI as an additional supplementary material.

We also added factor-level statistical tests to distinguish meaningful effects from sampling noise. For each prompt factor, we compare paired settings across the factorial design (e.g., with vs. without examples), and we now report mean differences, 95% confidence intervals, paired t-statistics, raw p-values, and Benjamini-Hochberg adjusted q-values to control the false discovery rate across multiple related tests. These analyses are reported for the relevant performance metrics and show that some factors do have systematic effects, but that these effects are often metric-dependent tradeoffs rather than uniformly beneficial improvements. For example, some prompt elements increase precision while decreasing recall, which would be obscured by reporting only aggregate means.

We also revised the results to make clearer how these inferential tests relate to the factorial variance decompositions. The factorial analyses identify how much variation is attributable to prompt elements, decoding settings, LLM choice, interactions, and residual variability. The paired tests then assess whether specific factor effects are directional and statistically reliable. In this way, the revised analysis separates two questions that were previously conflated: whether a factor explains variance, and whether changing that factor reliably improves or worsens a given performance metric.

Finally, we added structural variability analyses to complement the statistical testing. Because a large residual term may reflect either unstructured noise or meaningful variation in the generated outputs, we now examine replication stability and the overlap between raw predicted edge sets across repeated runs. These analyses show that Algorithms 1 and 2 are nearly deterministic under repetition, whereas Algorithm 3 produces substantially different edge sets across runs. This additional analysis helps interpret the residual term rather than simply treating it as unexplained noise.

3. Missing Failure Analysis

The article states that GPT-4o failed Algorithm 3 but does not give any information about such a crucial result. It is important to know the reasons behind the failures as much as successful runs. Record the types of particular errors, the deterministic or stochastic nature of failures, and the systematic failure of particular combinations of the LLM-algorithm. This knowledge is critical in helping practitioners to prevent such problems.

>> It's true, this called for further investigations. We revised the paper to clarify what 'failure' meant. The additional output-validity analyses show that the issue was not a parser-visible failure: in the audited corpus, we did not observe malformed or empty outputs (we now say it explicitly in the paper). Thus, this wasn't a failure in the sense of refusing the task, returning invalid syntax, or producing outputs that could not be processed. Instead, the failures in Algorithm 3 are substantive failures of recovery: the outputs were structurally valid, but they often failed to recover the expected cross-map links. Algorithm 3 is a tree-based exploratory procedure, so small differences in generated candidate concepts can compound across depth and lead to different edge sets. The problem is therefore not primarily invalid generation, but unstable output breadth and edge selection.

4. Lack of Mechanistic Explanation

The article shows that there is randomness but it is not really telling how and why this happens and how one can reduce it. The brief reference to mixture-of-experts routing is not enough. Compare randomness levels with measurable properties, e.g. prompt complexity, semantic ambiguity, or model uncertainty measures. Although it may not be possible to determine causal mechanisms, correlations would give practical information on reducing variability.

>> We thank the reviewer for this helpful suggestion. We added a new background subsection on potential sources of variability in LLM outputs. This subsection now distinguishes inference non-determinism, instability in the identity of the model being served, distributional bias, prompt-based constraint, and decoding-based control. So this goes beyond mentioning routing.
We agree with the reviewer that our analyses could not identify the internal causal mechanism inside proprietary LLMs. We therefore avoid claiming that a specific architectural mechanism is responsible for each residual term. We added experiments to directly manipulate decoding with open-weight LLMs, which would be the next strategy to try reducing variability after prompt engineering. Note that this combination already goes much beyond typical practices in LLM for conceptual modeling, as designed experiments for prompt engineering are rare in this context, and we have not seen controlled decoding in this space yet. The new experiments show that decoding is a practical lever for changing variability and performance, but they also show that there is no universal decoding choice that simply reduces variability while improving results. This is an important practical finding: reducing variability is not achieved by a single default setting, but requires treating decoding strategy as part of the experimental design.

5. No Baseline Comparisons

The paper evaluates LLM-based approaches without comparing them to traditional methods or human performance. Without baselines, readers cannot assess whether the variability-performance tradeoff is worthwhile. Include at least one non-LLM baseline method (such as

WordNet-based ontology matching or edit distance approaches) to contextualize the value proposition of using LLMs despite their inherent variability.

>> We added two baseline methods, one of which being the WordNet matching proposed by the reviewer. A dedicated subsection at the end of the results compares the LLM with non-LLM baselines, and the two methods are defined (both intuitively and formally) in our methods.

On behalf of the authors,

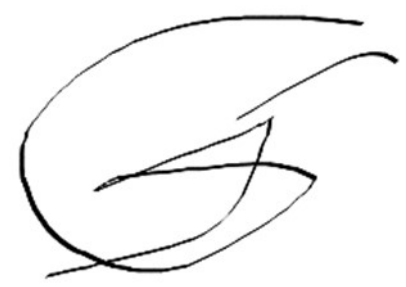

Philippe J. Giabbanelli
Full Professor
Virginia Modeling, Analysis, and Simulation Center (VMASC), Old Dominion University, USA
