# Supplementary material for: On the variability of generative artificial intelligence methods in conceptual modeling: an experimental evaluation on combining causal maps
Source: PLoS One. 2026 Oct 27;21(10):e0361281. doi: 10.1371/journal.pone.0361281 (PMC13712584; doi:10.1371/journal.pone.0361281)
Supplement: Supplementary file 4 [file pone.0361281.s012.pdf]

Reviewer #1: Having reviewed an earlier version of this manuscript, I am satisfied that the authors have addressed the principal concerns raised in that round. The addition of a second application domain, the strengthened statistical analyses, and the new background subsection on sources of variability collectively represent a substantial and genuine revision.

>> We appreciate the kind words of the reviewer, and the appreciation for the significant revision that we previously performed.

Reviewer #3: This is a really timely piece of work. However, I have a few points to raise:

- First off, as we both know, the LLM landscape (with things like GPT-5 and Gemini 2.5) moves at a breakneck pace. If a future researcher struggles to replicate your results, they will need to know if the failure is down to a change in the model's underlying routing or sampling. Without the exact API access dates and timestamps for your experiments, they will not be able to figure that out. I would therefore highly recommend adding these timestamps to make sure others can actually reproduce and build on what you have done.

>> As recommended, the new Table 3 provides the first and last access dates for each of the APIs used in our work, in support of replicability.

- Secondly, you are quite right that running a prompt just once usually leads to a fluke result, so your recommendation to run multiple repeats to see the full range of answers is spot on. But I wonder, what is the magic number of runs? You have done 5 repeats in your study, but that might not be quite enough to reach a stable conclusion, especially if random noise is causing most of the variation. So it would be brilliant if you could run a quick stability analysis to find the tipping point where adding more repeats stops changing the average. For instance, if the results are wildly different at 5 and 10 runs, but look practically identical at 50 and 60, then 50 runs becomes your recommended standard.

>> The reviewer is correct that our first submission focuses on 5 repeats, while noting that our previous revision showed how to estimate the total number of repetitions needed for a 95% confidence interval with a 5% relative half-width. We have now expanded the manuscript to better attract attention to this matter and the notion of a standard. Our results find that there is no (universal) standard since the replication budget is model-dependent. So rather than recommending a set number as a standard, we would recommend the use of statistical methods to identify what number of repeats is sufficient. The added text can be found in lines 589-594, 822-827.

- On another note, conceptual modelling is obviously a multi-step process (creation, verification, combination, and so on). If a user is just using an LLM to generate a single causal map from a transcript, is that 96% noise factor you mentioned still a threat? Or is that strictly down to the iterative, tree-based growth of Algorithm 3? So I would really want you to clarify which parts of the wider modelling lifecycle are high-risk for noise, and which are relatively stable.

>> We agree with the reviewer about modeling being an iterative process, which is exactly how we have taught and practiced it. However, this paper focuses on combining causal maps. We would not have results or other forms of evidence that allow us to generalize our findings to the wider modelling lifecycle. Our results suggest that, in the specific case of combining causal maps, some algorithms will have a low amount of noise and in others it will become the dominating factor, as the iterative tree-based growth is the culprit (as summarized by the reviewer). Beyond the scope of this paper, we believe that the modeling community would need studies at each point of the lifecycle to examine the amount of noise and, most importantly, whether it changes the modeling artifacts that we produce (i.e., what counts as a 'threat').

- Also, your shift from the old OFAT approach to a full DoE is a very sensible move. It is excellent for catching those tricky interaction effects between prompt elements. That said, DoE can be a bit of a mathematical headache for the average modeller who is not a statistician. I would therefore suggest recommending a few stat-lite tools or automated packages that can handle the heavy lifting for them. It might also be worth a quick discussion on how current platforms, perhaps utilising RAG or the PRODIGY framework, could build these analyses in as automated diagnostic tools. If you could offer a practical, shortcut version of your method, it would make managing all that stochastic noise much easier. In my opinion, that could help make reporting result distributions a standard, everyday practice in the field, which would be a fantastic contribution.

>> We appreciate this idea, as we also believe that it is important to keep the paper self-contained and accessible to the broad readership of the journal. Since recommending tools to manage stochastic noise would have made the paper longer (which is already at 40 pages), we have created a new supplementary online document to this effect consisting of three pages + short references. As recommended, the document directs individuals to tools that help to conduct a DoE. This new supplementary material S4 is referred to in the main document.

- The methodology looks fine, but I think you need to explain in more detail how the data was prepared for analysis, including any statistical methods. You mentioned the libraries used in Table 2, but I would recommend structuring and organising this section better to make it easier to follow.

>> As recommended, we have restructured and paper and expanded on these themes through two subsections titled "Data preparation and response variable" and "Statistical analysis". THey are both located under "Case study and Analyses".

- There is an inconsistency in how the F-1 score is written. In the Fig.6 caption, you use F-1 score, but in the figure's image, it is written as F1 score. Please make sure you use a consistent spelling for this throughout the entire paper.

>> Thank you for bringing this inconsistency to our attention. We now only say "F1 score".

- Regarding the Supporting Information, the step-by-step algorithm examples need a bit more

explanation. Right now, they are just listed without much detail, which makes them hard to follow.

>> We apologize for the difficulty in following the examples in our supplementary material S1. We expanded this material by providing more information as recommended.

Also, I could not access the S1 Table - Complete results file, so I am not able to review it.

>> We have checked again by downloading the file on a different computer, where it opens correctly. So the file is not corrupted and the most likely issue could be that the reviewer uses an older version of Excel. To avoid running into such issues, we converted the file into the more universal CSV format.

- Although they are mentioned in the main body of the paper, there are also 6 pages of figures at the very end of the manuscript (after the references) with no clear captions, which make it hard to tell what they show or how they fit into the paper. Please double check.

>> The editorial office kindly informed us that figures must be removed from the body of the paper and provided separately at the very end of the manuscript. The paper can only refer to them as Figures 1, 2, etc., but the captions remain in the paper while the figure files only provide the visuals. We renamed the figures to ensure that they can be accessed in the correct order, but we understand that having to go back-and-forth between figures and the papers is not a convenient workflow for reviews.

- The English is generally good, but please avoid using the first-person perspective (..we use precision..). Also, make sure the flow and coherence of the paper are consistent throughout.

>> Thank you for emphasizing the quality of writing. We have made several edits to ensure coherence and improve the flow, as shown in blue in the paper (examples include lines 57-59 and 1019-1020). We prefer using the first person perspective in our writing, as it clarifies the subject of the action, and we usually teach academic writing in this manner. However, we understand that this is a stylistic preference and that other writers may have different styles, so we respect that researchers bring their voices to their work.
