# Supplementary material for: On the variability of generative artificial intelligence methods in conceptual modeling: an experimental evaluation on combining causal maps
Source: PLoS One. 2026 Oct 27;21(10):e0361281. doi: 10.1371/journal.pone.0361281 (PMC13712584; doi:10.1371/journal.pone.0361281)
Supplement: Supplementary file 5 [file pone.0361281.s009.docx]

# Review Report: On the variability of generative artificial intelligence methods in conceptual modeling

**Manuscript ID:** PONE-D-25-54419
**Journal:** PLOS ONE

## General Assessment

The paper presents a significant issue in the conceptual modeling with the use of LLM, proving that randomness may take over even in the lowest temperature conditions. A methodological strength is the use of the factorial design. Nevertheless, the paper needs to be revised on some of its critical areas before publication. My particular areas of concern are as follows:

## Major Concerns

### 1. ****Limited Experimental Scope****

The article uses only one data of obesity causal maps, which seriously restricts the generalizability arguments. It is not enough to test only health-related models to make the conclusion that the results can be generalized to conceptual modeling tasks. The semantic structures in different domains (business processes, software engineering, environmental systems) are radically different and can have different interactions with the capabilities of LLM. Add at least one more domain with other features to reinforce arguments that it is cross-domain.

### 2. ****Insufficient Statistical Rigor****

The article does not have basic statistical hypothesis testing. No ANOVA F-tests, p-values, and confidence intervals are provided and it is impossible to differentiate between meaningful effects and sampling noise. The decision to use 5 replications does not seem to be justified by power analysis. Add formal statistical testing with multiple comparison adjustments, provide confidence intervals of all performance measures, and rationalize replication figures by demonstrating power or convergence.

### 3. ****Missing Failure Analysis****

The article states that GPT-4o failed Algorithm 3 but does not give any information about such a crucial result. It is important to know the reasons behind the failures as much as successful runs. Record the types of particular errors, the deterministic or stochastic nature of failures, and the systematic failure of particular combinations of the LLM-algorithm. This knowledge is critical in helping practitioners to prevent such problems.

### 4. ****Lack of Mechanistic Explanation****

The article shows that there is randomness but it is not really telling how and why this happens and how one can reduce it. The brief reference to mixture-of-experts routing is not enough. Compare randomness levels with measurable properties, e.g. prompt complexity, semantic ambiguity, or model uncertainty measures. Although it may not be possible to determine causal mechanisms, correlations would give practical information on reducing variability.

### 5. ****No Baseline Comparisons****

The paper evaluates LLM-based approaches without comparing them to traditional methods or human performance. Without baselines, readers cannot assess whether the variability-performance tradeoff is worthwhile. Include at least one non-LLM baseline method (such as WordNet-based ontology matching or edit distance approaches) to contextualize the value proposition of using LLMs despite their inherent variability.

**Regards**
