# Supplementary material for: On the variability of generative artificial intelligence methods in conceptual modeling: an experimental evaluation on combining causal maps
Source: PLoS One. 2026 Oct 27;21(10):e0361281. doi: 10.1371/journal.pone.0361281 (PMC13712584; doi:10.1371/journal.pone.0361281)
Supplement: Peer review history

# Peer review: Decision Letter 0

**DOI:** 10.1371/journal.pone.0361281.r001

**Authors:** Tjeerd olde Scheper

2 Mar 2026

On the variability of generative artificial intelligence methods in conceptual modeling: an experimental evaluation on combining causal maps

PLOS One

Dear Dr. Giabbanelli,

Thank you for submitting your manuscript to PLOS ONE. After careful consideration, we feel that it has merit but does not fully meet PLOS ONE’s publication criteria as it currently stands. Therefore, we invite you to submit a revised version of the manuscript that addresses the points raised during the review process.

Please submit your revised manuscript by Apr 16 2026 11:59PM. If you will need more time than this to complete your revisions, please reply to this message or contact the journal office at plosone@plos.org. When you're ready to submit your revision, log on to https://www.editorialmanager.com/pone/ and select the 'Submissions Needing Revision' folder to locate your manuscript file.

Please include the following items when submitting your revised manuscript:

- A letter that responds to each point raised by the academic editor and reviewer(s). You should upload this letter as a separate file labeled 'Response to Reviewers'.
- A marked-up copy of your manuscript that highlights changes made to the original version. You should upload this as a separate file labeled 'Revised Manuscript with Track Changes'.
- An unmarked version of your revised paper without tracked changes. You should upload this as a separate file labeled 'Manuscript'.

If you would like to make changes to your financial disclosure, please include your updated statement in your cover letter. Guidelines for resubmitting your figure files are available below the reviewer comments at the end of this letter.

If applicable, we recommend that you deposit your laboratory protocols in protocols.io to enhance the reproducibility of your results. Protocols.io assigns your protocol its own identifier (DOI) so that it can be cited independently in the future. For instructions see: https://journals.plos.org/plosone/s/submission-guidelines#loc-laboratory-protocols. Additionally, PLOS ONE offers an option for publishing peer-reviewed Lab Protocol articles, which describe protocols hosted on protocols.io. Read more information on sharing protocols at https://plos.org/protocols?utm_medium=editorial-email&utm_source=authorletters&utm_campaign=protocols.

We look forward to receiving your revised manuscript.

Kind regards,

Tjeerd V. olde Scheper, Ph.D.

Academic Editor

PLOS One

Journal Requirements:

When submitting your revision, we need you to address these additional requirements.

1. Please ensure that your manuscript meets PLOS ONE's style requirements, including those for file naming. The PLOS ONE style templates can be found at

https://journals.plos.org/plosone/s/file?id=wjVg/PLOSOne_formatting_sample_main_body.pdf and

https://journals.plos.org/plosone/s/file?id=ba62/PLOSOne_formatting_sample_title_authors_affiliations.pdf

2. Please note that PLOS One has specific guidelines on code sharing for submissions in which author-generated code underpins the findings in the manuscript. In these cases, we expect all author-generated code to be made available without restrictions upon publication of the work. Please review our guidelines at https://journals.plos.org/plosone/s/materials-and-software-sharing#loc-sharing-code and ensure that your code is shared in a way that follows best practice and facilitates reproducibility and reuse.

3. Thank you for uploading your study's underlying data set. Unfortunately, the repository you have noted in your Data Availability statement does not qualify as an acceptable data repository according to PLOS's standards.

At this time, please upload the minimal data set necessary to replicate your study's findings to a stable, public repository (such as figshare or Dryad) and provide us with the relevant URLs, DOIs, or accession numbers that may be used to access these data. For a list of recommended repositories and additional information on PLOS standards for data deposition, please see https://journals.plos.org/plosone/s/recommended-repositories.

4.If the reviewer comments include a recommendation to cite specific previously published works, please review and evaluate these publications to determine whether they are relevant and should be cited. There is no requirement to cite these works unless the editor has indicated otherwise.

Additional Editor Comments:

Kindly address the comments from both reviewers, especially with respect to validation and include in the revision a focus on the requested improvements on evidential support of the claims made in your manuscript.

Please ensure that your abstract and conclusion reflect the improvements you will make on the basis of these reviews.

[Note: HTML markup is below. Please do not edit.]

Reviewer's Responses to Questions

**Comments to the Author**

1. Is the manuscript technically sound, and do the data support the conclusions?

Reviewer #1: Partly

Reviewer #2: Yes

**********

2. Has the statistical analysis been performed appropriately and rigorously?

Reviewer #1: Yes

Reviewer #2: No

**********

3. Have the authors made all data underlying the findings in their manuscript fully available?

The PLOS Data policy

Reviewer #1: No

Reviewer #2: Yes

**********

4. Is the manuscript presented in an intelligible fashion and written in standard English?

Reviewer #1: Yes

Reviewer #2: Yes

**********

Reviewer #1: The manuscript examines the extent to which variability in contemporary large language models shapes outcomes in conceptual modeling, using the task of combining causal maps as an empirical testbed. The authors motivate their work from the rapidly expanding literature on LLM-assisted conceptual modeling and correctly identify a methodological blind spot: most prior studies either rely on default temperature settings or set temperature to zero while implicitly assuming quasi-deterministic behavior. Building on this observation, the paper derives its central hypotheses from work on prompt engineering, model architecture, and stochastic decoding, and operationalizes them through a carefully constructed full factorial Design of Experiments with replication. Three representative model-combination algorithms are implemented, spanning direct edge generation, creative expansion via embeddings, and multi-step tree-based reasoning, and are evaluated across three LLM families and two successive versions per family. The experimental design is rigorous, transparent, and well documented, and the discussion convincingly demonstrates that variability can dominate performance outcomes, even at minimal temperature settings, thereby challenging common assumptions about reproducibility in LLM-based modeling studies. By emphasizing distributional outcomes rather than single-run results, the manuscript makes a valuable methodological contribution to the conceptual modeling community ￼.

A major point of criticism concerns the interpretation of variability. Throughout the manuscript, large error terms in the factorial analyses are primarily framed as stochastic noise that undermines experimental control. However, variability may itself be structured and informative rather than purely random. For example, Huff and Ulakci’s study in Scientific Reports shows that LLMs systematically fail to replicate human metacognitive judgments of learning (JOLs) even when surface-level performance aligns with humans, revealing a structured divergence in how models interpret and predict memory outcomes ￼. This suggests that variability across runs and models may reflect underlying representational ambiguity or limitations in how prompts constrain the model’s internal hypothesis space, rather than mere stochastic noise. Integrating this perspective would help reframe the authors’ key finding: instead of concluding that prompt elements “matter very little” when randomness dominates, one could argue that prompts fail to sufficiently constrain conceptual inference, leading to systematic differences in latent reasoning trajectories.

A related point of critique is that the discussion remains largely descriptive with respect to architectural differences. While variations across LLM families and versions are documented, the manuscript stops short of linking these differences to specific computational mechanisms. For instance, work by Ulakci, Göbel, and Huff in PeerJ Computer Science dissects how LLM outputs can align with human cognitive data on some dimensions (e.g., relatedness and memorability ratings) yet diverge in others, indicating that variability often has interpretable structure connected to context dependency rather than being irreducible noise ￼. This implies that treating "the LLM" as a single factor may obscure meaningful sub-mechanisms such as decoding strategy differences, latent uncertainty, or multi-step amplification effects. Explicitly engaging with these ideas would strengthen the explanatory power of the manuscript’s factorial analysis.

Another substantive issue is the limited connection to broader theory in conceptual modeling and cognition. Although the manuscript cites work on integronsters and model integration, the implications of the findings for how conceptual models function as cognitive or epistemic artifacts are not fully developed. The evaluation framework assumes a single ground-truth causal map and treats deviations as errors, yet conceptual modeling literature emphasizes that combining partial perspectives can legitimately yield emergent, and sometimes desirable, structures. A more explicit discussion of this tension would clarify whether variability reflects genuine model ambiguity, evaluation limitations, or systematic divergence due to underconstrained inference. Relatedly, reliance on a single substantive domain, obesity, limits claims about generality across domains with different semantic structures or contextual complexities.

On more minor points, the introduction could be tightened to foreground the research questions earlier and reduce some repetition in the literature review. Some terminology alternates between "knowledge maps", "conceptual models", and "causal maps" without consistent definitions, which may confuse readers outside the immediate community. The presentation of results, especially the large factorial tables, would benefit from additional interpretive guidance or visual summaries to support readers less familiar with Design of Experiments. From an open science perspective, the authors should be commended for sharing data and scripts via OSF, but the manuscript would be strengthened by an explicit discussion of preregistration and by a principled justification of the number of replications, for example via power or precision considerations rather than feasibility alone. Reporting confidence intervals or distributional plots alongside mean performance would further reinforce the paper’s core message about variability.

Relevant literature that could strengthen the manuscript includes work on metacognitive differences between humans and LLMs, such as Huff & Ulakci’s study demonstrating structured divergence in predictive judgment tasks; analyses showing how different architectural and decoding strategies contribute to output behavior; and broader cognitive modeling perspectives on how emergent structures and underdetermination affect conceptual inference. Engaging more directly with these sources would help position variability not only as a methodological challenge but also as a theoretically informative phenomenon that can advance our understanding of both LLM behavior and conceptual modeling practices.

I attempted to review the data and materials at the OSF repository linked in the manuscript (https://osf.io/kgdru/), but the project page did not load or reveal any accessible files at the time of review, suggesting that the repository may be private, restricted, or otherwise unavailable for public inspection (I checked it on different browsers). Because OSF projects can be set to public or private at the discretion of the authors, and because successful reuse of data depends on discoverability and access, this prevented me from independently evaluating the shared data and scripts in support of the manuscript’s claims.

References:

Huff, M., & Ulakci, E. (2025). Judgments of learning distinguish humans from large language models in predicting memory. Scientific Reports, 15(1), 35030. https://doi.org/10.1038/s41598-025-22290-x

Ulakci, E., Göbel, J. P., & Huff, M. (2025). Towards a psychology of machines: large language models predict human memory. PeerJ Computer Science, 11, e3324. https://doi.org/10.7717/peerj-cs.3324

Reviewer #2: All the recommendations are written in detail in the attached file. The attached file contains main points of concerns that need to be assessed before the publication of this paper. Although this paper is sound and makes great impact in the research of Gen AI, it still needs to be done in better way.

**********

what does this mean?). If published, this will include your full peer review and any attached files.

If you choose “no”, your identity will remain anonymous but your review may still be made public.

**Do you want your identity to be public for this peer review?**  For information about this choice, including consent withdrawal, please see our Privacy Policy

Reviewer #1: No

Reviewer #2: No

**********

[NOTE: If reviewer comments were submitted as an attachment file, they will be attached to this email and accessible via the submission site. Please log into your account, locate the manuscript record, and check for the action link "View Attachments". If this link does not appear, there are no attachment files.]

To ensure your figures meet our technical requirements, please review our figure guidelines: https://journals.plos.org/plosone/s/figures

You may also use PLOS’s free figure tool, NAAS, to help you prepare publication quality figures: https://journals.plos.org/plosone/s/figures#loc-tools-for-figure-preparation.

NAAS will assess whether your figures meet our technical requirements by comparing each figure against our figure specifications.

Attachment Submitted filename: Variability of generative artificial intelligence methods.docx

# Author comment: Author response to Decision Letter 1

**DOI:** 10.1371/journal.pone.0361281.r002

30 Apr 2026

Please see our detailed response letter attached. For the convenience of the reviewers and editors, our responses are clearly formatted in blue and the original points are shown in black.

Attachment Submitted filename: Response letter.pdf

# Peer review: Decision Letter 1

**DOI:** 10.1371/journal.pone.0361281.r003

**Authors:** Tjeerd olde Scheper

21 Aug 2026

Dear Dr. Giabbanelli,

Please submit your revised manuscript by Oct 05 2026 11:59PM. If you will need more time than this to complete your revisions, please reply to this message or contact the journal office at plosone@plos.org. When you're ready to submit your revision, log on to https://www.editorialmanager.com/pone/ and select the 'Submissions Needing Revision' folder to locate your manuscript file.

- A letter that responds to each point raised by the academic editor and reviewer(s). You should upload this letter as a separate file labeled 'Response to Reviewers'.
- A marked-up copy of your manuscript that highlights changes made to the original version. You should upload this as a separate file labeled 'Revised Manuscript with Track Changes'.
- An unmarked version of your revised paper without tracked changes. You should upload this as a separate file labeled 'Manuscript'.

If you would like to make changes to your financial disclosure, please include your updated statement in your cover letter. Guidelines for resubmitting your figure files are available below the reviewer comments at the end of this letter.

If applicable, we recommend that you deposit your laboratory protocols in protocols.io to enhance the reproducibility of your results. Protocols.io assigns your protocol its own identifier (DOI) so that it can be cited independently in the future. For instructions see: https://journals.plos.org/plosone/s/submission-guidelines#loc-laboratory-protocols. Additionally, PLOS One offers an option for publishing peer-reviewed Lab Protocol articles, which describe protocols hosted on protocols.io. Read more information on sharing protocols at https://plos.org/protocols?utm_medium=editorial-email&utm_source=authorletters&utm_campaign=protocols.

As the corresponding author, your ORCID iD is verified in the submission system and will appear in the published article. PLOS supports the use of ORCID, and we encourage all coauthors to register for an ORCID iD and use it as well. Please encourage your coauthors to verify their ORCID iD within the submission system before final acceptance, as unverified ORCID iDs will not appear in the published article. *Only*  the individual author can complete the verification step; PLOS staff *cannot*  verify ORCID iDs on behalf of authors.

We look forward to receiving your revised manuscript.

Kind regards,

Tjeerd V. olde Scheper, Ph.D.

Academic Editor

PLOS One

Journal Requirements:

If the reviewer comments include a recommendation to cite specific previously published works, please review and evaluate these publications to determine whether they are relevant and should be cited. There is no requirement to cite these works unless the editor has indicated otherwise.

[Note: HTML markup is below. Please do not edit.]

Reviewers' comments:

Reviewer's Responses to Questions

**Comments to the Author**

Reviewer #1: All comments have been addressed

Reviewer #3: (No Response)

**********

2. Is the manuscript technically sound, and do the data support the conclusions?

Reviewer #1: Yes

Reviewer #3: Partly

**********

3. Has the statistical analysis been performed appropriately and rigorously?

Reviewer #1: Yes

Reviewer #3: No

**********

4. Have the authors made all data underlying the findings in their manuscript fully available?

The PLOS Data policy

Reviewer #1: Yes

Reviewer #3: No

**********

5. Is the manuscript presented in an intelligible fashion and written in standard English?

Reviewer #1: Yes

Reviewer #3: Yes

**********

Reviewer #1: Having reviewed an earlier version of this manuscript, I am satisfied that the authors have addressed the principal concerns raised in that round. The addition of a second application domain, the strengthened statistical analyses, and the new background subsection on sources of variability collectively represent a substantial and genuine revision.

Reviewer #3: This is a really timely piece of work. However, I have a few points to raise:

- First off, as we both know, the LLM landscape (with things like GPT-5 and Gemini 2.5) moves at a breakneck pace. If a future researcher struggles to replicate your results, they will need to know if the failure is down to a change in the model's underlying routing or sampling. Without the exact API access dates and timestamps for your experiments, they will not be able to figure that out. I would therefore highly recommend adding these timestamps to make sure others can actually reproduce and build on what you have done.

- Secondly, you are quite right that running a prompt just once usually leads to a fluke result, so your recommendation to run multiple repeats to see the full range of answers is spot on. But I wonder, what is the magic number of runs? You have done 5 repeats in your study, but that might not be quite enough to reach a stable conclusion, especially if random noise is causing most of the variation. So it would be brilliant if you could run a quick stability analysis to find the tipping point where adding more repeats stops changing the average. For instance, if the results are wildly different at 5 and 10 runs, but look practically identical at 50 and 60, then 50 runs becomes your recommended standard.

- On another note, conceptual modelling is obviously a multi-step process (creation, verification, combination, and so on). If a user is just using an LLM to generate a single causal map from a transcript, is that 96% noise factor you mentioned still a threat? Or is that strictly down to the iterative, tree-based growth of Algorithm 3? So I would really want you to clarify which parts of the wider modelling lifecycle are high-risk for noise, and which are relatively stable.

- Also, your shift from the old OFAT approach to a full DoE is a very sensible move. It is excellent for catching those tricky interaction effects between prompt elements. That said, DoE can be a bit of a mathematical headache for the average modeller who is not a statistician. I would therefore suggest recommending a few stat-lite tools or automated packages that can handle the heavy lifting for them. It might also be worth a quick discussion on how current platforms, perhaps utilising RAG or the PRODIGY framework, could build these analyses in as automated diagnostic tools. If you could offer a practical, shortcut version of your method, it would make managing all that stochastic noise much easier. In my opinion, that could help make reporting result distributions a standard, everyday practice in the field, which would be a fantastic contribution.

- The methodology looks fine, but I think you need to explain in more detail how the data was prepared for analysis, including any statistical methods. You mentioned the libraries used in Table 2, but I would recommend structuring and organising this section better to make it easier to follow.

- There is an inconsistency in how the F-1 score is written. In the Fig.6 caption, you use F-1 score, but in the figure's image, it is written as F1 score. Please make sure you use a consistent spelling for this throughout the entire paper.

- Regarding the Supporting Information, the step-by-step algorithm examples need a bit more explanation. Right now, they are just listed without much detail, which makes them hard to follow. Also, I could not access the S1 Table - Complete results file, so I am not able to review it.

- Although they are mentioned in the main body of the paper, there are also 6 pages of figures at the very end of the manuscript (after the references) with no clear captions, which make it hard to tell what they show or how they fit into the paper. Please double check.

- The English is generally good, but please avoid using the first-person perspective (..we use precision..). Also, make sure the flow and coherence of the paper are consistent throughout.

**********

what does this mean?). If published, this will include your full peer review and any attached files.

If you choose “no”, your identity will remain anonymous but your review may still be made public.

**Do you want your identity to be public for this peer review?**  For information about this choice, including consent withdrawal, please see our Privacy Policy

Reviewer #1: No

Reviewer #3: No

**********

[NOTE: If reviewer comments were submitted as an attachment file, they will be attached to this email and accessible via the submission site. Please log into your account, locate the manuscript record, and check for the action link "View Attachments". If this link does not appear, there are no attachment files.]

To ensure your figures meet our technical requirements, please review our figure guidelines: https://journals.plos.org/plosone/s/figures

You may also use PLOS’s free figure tool, NAAS, to help you prepare publication quality figures: https://journals.plos.org/plosone/s/figures#loc-tools-for-figure-preparation.

NAAS will assess whether your figures meet our technical requirements by comparing each figure against our figure specifications.

# Author comment: Author response to Decision Letter 2

**DOI:** 10.1371/journal.pone.0361281.r004

16 Sep 2026

Please see our detailed response letter to the reviewers. Thank you.

Attachment Submitted filename: Response to Reviewers.pdf

# Peer review: Decision Letter 2

**DOI:** 10.1371/journal.pone.0361281.r005

**Authors:** Tjeerd olde Scheper

9 Oct 2026

On the variability of generative artificial intelligence methods in conceptual modeling: an experimental evaluation on combining causal maps

PONE-D-25-54419R2

Dear Dr. Giabbanelli,

We’re pleased to inform you that your manuscript has been judged scientifically suitable for publication and will be formally accepted for publication once it meets all outstanding technical requirements.

Within one week, you’ll receive an e-mail detailing the required amendments. When these have been addressed, you’ll receive a formal acceptance letter and your manuscript will be scheduled for publication.

An invoice will be generated when your article is formally accepted. Please note, if your institution has a publishing partnership with PLOS and your article meets the relevant criteria, all or part of your publication costs will be covered. Please make sure your user information is up-to-date by logging into Editorial Manager at Editorial Manager® and clicking the ‘Update My Information' link at the top of the page. For questions related to billing, please contact billing support.

If your institution or institutions have a press office, please notify them about your upcoming paper to help maximize its impact. If they’ll be preparing press materials, please inform our press team as soon as possible -- no later than 48 hours after receiving the formal acceptance. Your manuscript will remain under strict press embargo until 2 pm Eastern Time on the date of publication. For more information, please contact onepress@plos.org.

Kind regards,

Tjeerd V. olde Scheper, Ph.D.

Academic Editor

PLOS One

Additional Editor Comments (optional):

Reviewers' comments:

Reviewer's Responses to Questions

**Comments to the Author**

Reviewer #3: All comments have been addressed

**********

2. Is the manuscript technically sound, and do the data support the conclusions?

Reviewer #3: Yes

**********

3. Has the statistical analysis been performed appropriately and rigorously?

Reviewer #3: Yes

**********

4. Have the authors made all data underlying the findings in their manuscript fully available?

The PLOS Data policy

Reviewer #3: Yes

**********

5. Is the manuscript presented in an intelligible fashion and written in standard English?

Reviewer #3: Yes

**********

Reviewer #3: I am satisfied with the revisions made and recommend the manuscript for publication. Please note, however, that Tables 8 and 11 appear cut off at the margins; I recommend correcting this alignment issue prior to final publication.

**********

what does this mean?). If published, this will include your full peer review and any attached files.

If you choose “no”, your identity will remain anonymous but your review may still be made public.

**Do you want your identity to be public for this peer review?**  For information about this choice, including consent withdrawal, please see our Privacy Policy

Reviewer #3: No

**********

# Editor report: Acceptance letter

**DOI:** 10.1371/journal.pone.0361281.r006

**Authors:** Tjeerd olde Scheper

PONE-D-25-54419R2

PLOS One

Dear Dr. Giabbanelli,

I'm pleased to inform you that your manuscript has been deemed suitable for publication in PLOS One. Congratulations! Your manuscript is now being handed over to our production team.

At this stage, our production department will prepare your paper for publication. This includes ensuring the following:

* All references, tables, and figures are properly cited

* All relevant supporting information is included in the manuscript submission,

* There are no issues that prevent the paper from being properly typeset

You will receive further instructions from the production team, including instructions on how to review your proof when it is ready. Please keep in mind that we are working through a large volume of accepted articles, so please give us a few days to review your paper and let you know the next and final steps.

Lastly, if your institution or institutions have a press office, please let them know about your upcoming paper now to help maximize its impact. If they'll be preparing press materials, please inform our press team within the next 48 hours. Your manuscript will remain under strict press embargo until 2 pm Eastern Time on the date of publication. For more information, please contact onepress@plos.org.

You will receive an invoice from PLOS for your publication fee after your manuscript has reached the completed accept phase. If you receive an email requesting payment before acceptance or for any other service, this may be a phishing scheme. Learn how to identify phishing emails and protect your accounts at https://explore.plos.org/phishing.

If we can help with anything else, please email us at customercare@plos.org.

Thank you for submitting your work to PLOS One and supporting open access.

Kind regards,

PLOS One Editorial Office Staff

on behalf of

Dr. Tjeerd V. olde Scheper

Academic Editor

PLOS One
